# Effectiveness of Mobile Health–Based Self-Management Programs on Health-Related Outcomes in Patients With Chronic Obstructive Pulmonary Disease: Systematic Review and Meta-Analysis

**DOI:** 10.2196/74967

**Published:** 2025-12-29

**Authors:** Galuh Nawang Prawesti, Pinyi Lo, Made Ary Sarasmita, Hsiang Yin Chen

**Affiliations:** 1 Department of Clinical Pharmacy, School of Pharmacy, College of Pharmacy, Taipei Medical University, No.301, Yuantong Road, Zhonghe District, Taipei, 235, Taiwan, 886 2-2736-1661 ext 6175; 2 Department of Clinical and Community Pharmacy, Faculty of Pharmacy, Widya Mandala Surabaya Catholic University, Surabaya, Indonesia; 3 Department of Pharmacy, Shuang Ho Hospital, Taipei Medical University, Taipei, Taiwan; 4 Program Study of Pharmacy, Faculty of Mathematics and Science, Udayana University, Badung, Indonesia; 5 Department of Pharmacy, Wan Fang Hospital, Taipei Medical University, Taipei, Taiwan

**Keywords:** self-management, chronic disease, COPD, chronic obstructive pulmonary disease, dyspnea, mHealth, mobile health

## Abstract

**Background:**

The progression of chronic obstructive pulmonary disease (COPD) leads to increased morbidity and mortality, emphasizing the need for effective self-management. Challenges such as accessibility, cost, and patient engagement hinder self-management efforts, underscoring the need for evidence-based mobile health (mHealth) interventions.

**Objective:**

This meta-analysis evaluated randomized controlled trials (RCTs) on the effectiveness of mHealth self-management programs for COPD, focusing on the modified Medical Research Council (mMRC) dyspnea scale, the 6-minute walking test (6MWT), and the St. George’s Respiratory Questionnaire (SGRQ) score. The secondary outcomes include quality-adjusted life years and costs as economic outcomes; exacerbation, hospitalization, and emergency room and clinic visits as clinical outcomes; and self-efficacy as a humanistic outcome.

**Methods:**

The inclusion criteria encompassed RCTs involving patients with COPD aged 18 years and older, comparing mHealth-based self-management programs to non-mHealth interventions, with outcomes measured using the mMRC dyspnea scale, 6MWT, and SGRQ score. Exclusion criteria included observational studies, reviews, qualitative research, protocols, and non-English publications. A comprehensive search was conducted across PubMed, Embase, CINAHL, Web of Science, Cochrane, and Scopus using predefined keywords and MeSH terms for studies published between January 2015 and September 2024. The risk of bias was assessed using the Cochrane Risk-of-Bias 2 tool. Data extraction encompassed study characteristics, interventions, comparators, and outcomes. Meta-analyses were performed for outcomes reported in at least 3 RCTs using R software (version 4.2.2; R Foundation for Statistical Computing).

**Results:**

This systematic review included 36 RCTs from diverse geographical regions, encompassing 5606 patients. The meta-analysis revealed significant improvements in the mMRC dyspnea scale (mean difference −0.65, 95% CI −1.14 to −0.16; *P*=.02) and 6MWT (mean difference 25.96 m, 95% CI 10.05 m to 41.87 m; *P*=.004) in the mHealth intervention group compared to controls. However, no statistical significance was observed in the SGRQ total score (mean difference −3.56, 95% CI −7.39 to 0.27; *P*=.07). A total of 2 studies reported economic results, with a possible statistically significant decrease in the mean cost per patient (€3547 vs €4831 [US $4118.4 vs US $5609.24]; *P*=.01), but no statistically significant difference in quality-adjusted life years (0.485 vs 0.491; *P*=.73). A total of 5 studies reported substantial reductions in hospital admissions. Additionally, 1 study each reported significant improvements in time to first readmission for COPD exacerbations, clinic visits, mortality rates, and exacerbation frequencies. A single study reported a significant improvement in self-efficacy, as measured by the Pulmonary Rehabilitation Adapted Index of Self-Efficacy scores.

**Conclusions:**

This review supports the Global Initiative for Chronic Obstructive Lung Disease 2025 recommendations, highlighting mHealth as a supplementary clinical tool requiring patient education, ethical compliance, and informed consent. Further large-scale studies are needed to refine mHealth tools, ensuring accessibility, long-term safety, and effectiveness across diverse populations and outcome domains.

## Introduction

Increasing attention to the potential approach for self-management has highlighted the role of digital intervention for people with chronic obstructive pulmonary disease (COPD) in recent years. The COPD severity and progression cause morbidity and mortality in patients. Adequate self-care and self-management play essential roles in a patient’s disease progression [1]. Delivering self-management programs to reduce the burden of COPD remains challenging due to competing demands, time constraints, distance, and costs [2]. Encouraging patients with COPD to engage in self-management programs actively has proven difficult [3], due to limited willingness, experiencing barriers to self-management [4], a lack of literacy, and low understanding of treatment [5]. A well-designed strategy for delivering self-management interventions in patients with COPD is essential to enhance economic, clinical, and humanistic outcomes (ECHOs) [6,7]. The application of mHealth self-management is recommended and encouraged by the World Health Organization (WHO) and the 2025 Global Initiative for Chronic Obstructive Lung Disease (GOLD) guidelines [8,9].

Applying mobile health (mHealth) technologies to patients with COPD involves real-time monitoring of vital signs and clinical symptoms [10], as well as patient education, physical exercise, and pulmonary rehabilitation (PR) [11]. mHealth self-management interventions can be implemented through a range of digital tools, including websites, smartphone apps, telecommunication systems, and wearable devices [3,4,12]. It offers advantages in reducing transportation barriers to physical activities, promoting available access to communicate with health care providers, and gaining interest among older adults [13]. mHealth interventions have shown potential to improve health-related quality of life (QoL) in the short term (<6 months) [14]. It may promote daily lifestyle changes, physical activity, and exercise capacity [1,15,16], and improve dyspnea symptoms with lower costs [10,14,17]. However, other studies found that the pooled effect sizes for physical function, dyspnea symptoms, and QoL were not significant [18-20]. Many studies have insufficient results to establish their longer-term effectiveness [15,21,22]. It is still unclear how effectively mHealth can improve QoL and exercise capacity in patients with COPD.

Technologies are rapidly increasing, and thus it is essential to identify effective current interventions to help promote mHealth for COPD self-management. Meta-analyses to date have used various eligibility criteria, including only trials with smartphones [18,23,24], excluding trials that involved health care providers [1], and including trials with various study designs and sample characteristics [14,23]. The effectiveness of mHealth in COPD still needs to be rigorously assessed. In this systematic review, we aimed to update existing evidence on the effectiveness of mHealth interventions in delivering self-management programs compared to non-mHealth approaches for patients with COPD, focusing on health-related outcomes, particularly dyspnea symptoms, exercise capacity, and QoL.

## Methods

### Study Design and Search Strategy

This study was performed according to the PRISMA (Preferred Reporting Items for Systematic Reviews and Meta-analyses) 2020 guidelines [25]. It examined RCTs that compared the effectiveness of mHealth-based self-management programs with non-mHealth programs for patients with COPD. The protocol for drafting the systematic reviews method was registered in the PROSPERO (International Prospective Register of Systematic Reviews; CRD42020181157).

The research questions of this study were derived according to the PICOS (population, interventions, comparators, outcomes, and study designs) framework [26]. Studies targeting adults aged 18 years and older with COPD were included in the analysis with no restriction on race, ethnicity, geography, or sex to ensure an extensive population. The intervention had to include mHealth for COPD self-management programs. The study comparators contained non-mHealth interventions, including usual, conventional, routine, or standard care, written materials, or face-to-face programs. The desired outcomes for this study were the modified Medical Research Council (mMRC) Dyspnea Scale, exercise capacity according to the 6-minute walking test (6MWT), and health-related QoL by St. George’s Respiratory Questionnaire (SGRQ) score in RCT designs. Only studies in English were included. Observational studies, reviews, qualitative research, and protocols were excluded.

A bibliographic search was conducted to identify relevant original articles using electronic database systems, including PubMed, Ovid Medline, Embase, CINAHL, Web of Science Collection, Cochrane Database, and Scopus. The search strategy was designed, and a database search was performed in October 2024 (see Checklist 1for the PRISMA [Preferred Reporting Items for Systematic Reviews and Meta-Analyses] 2020 checklist). The terms included in the search strategy used Medical Subject Headings (MeSH) terms, Boolean operators, and a filter publication type “randomized controlled trials,” with the main keywords in the query box of “chronic obstructive pulmonary disease (COPD),” “mobile health,” and “self-management.” For full search strategies, refer to Multimedia Appendix 1. The search covered all data from January 2015 to September 30, 2024. mHealth, as defined by the WHO, is a medical and public health practice supported by mobile devices, such as mobile phones, patient monitoring devices, personal digital assistants, and other wireless devices [27].

### Screening Process of the Studies

A standardized identification and screening process was applied to ensure that all eligible RCTs were included. Three authors (GNP, PL, and MAS) independently screened relevant studies based on their titles and abstracts and assessed full-text articles to ensure they met the eligibility criteria. Any unclear or missing information was sought through contacting the corresponding authors via email. The results of each screening round were compared and reviewed until a consensus was reached. The studies were categorized based on their characteristics, such as the geographic distribution, types of mHealth intervention, involvement of health care professionals, etc. The corresponding author was involved in the discussion of any discrepancy until agreement was reached.

### Risk-of-Bias and Publication Bias Assessments

The quality of the included studies was assessed using the Cochrane Collaboration’s Risk-of-Bias tool for RCTs (version 2.0) [28] to evaluate bias arising from the randomization process, deviations from intended interventions, missing outcome data, measurement of the outcome, or selection of the reported results. Each domain was rated as “low risk of bias,” “some concerns,” or “high risk of bias” as reviewed by the authors. Publication bias across studies was assessed using a funnel plot and an Egger test for outcomes with a minimum of 10 studies included [29].

### Outcome Definition and Data Synthesis

A template was developed to extract relevant data from the original studies. The authors independently extracted the data, including the authors’ information, clinical setting, intervention, comparator, sample size, demographics, and types of study outcomes. The ECHO model was applied to categorize outcomes. The interconnection of health dimensions may lead to some overlap and ambiguity, as some tools (eg, Patient Health Questionnaire-9 and COPD Assessment Test) inform both clinical decisions and patient perceptions [30-32]. This paper classified the economic outcomes referred to as the total medical care costs of treatment options, typically evaluated in the context of clinical or humanistic results (eg, cost, quality-adjusted life year [QALY], and cost-utility analysis). Clinical outcomes in this study represented health events arising from the disease or its treatment (eg, hospital admission, emergency room [ER] visit, and exacerbation), while humanistic outcomes captured functional status or quality of life assessed through self-reported measures (eg, 36-Item Short Form Health Survey and EQ-5D [EuroQol 5-Dimensions]) [7].

Studies reporting dyspnea symptoms using the mMRC Dyspnea Scale, exercise capacity using the 6MWT, and the total score from reported QoL using the COPD-specific questionnaire of the SGRQ as primary outcomes were pooled using a random effects model. Economic (QALYs and costs), clinical (exacerbation, hospitalization, ER and clinic visits, and mortality), and other humanistic outcomes (self-efficacy) as the secondary outcomes were descriptively reported.

A meta-analysis was conducted for any of the outcomes that were reported in 3 or more RCTs [6]. Results of the expected outcomes were converted to effect sizes, and point estimates were reported, such as mean, SD, SE, and mean difference in both the intervention and control groups. Statistical significance was defined as *P*<.05, and sensitivity analysis was conducted to assess the robustness of the primary outcomes. Heterogeneity was identified by *I*^2^ statistics, with high heterogeneity interpreted for those studies with values exceeding 50% [33]. Hedges *g*, a variation of Cohen *d* for correcting possible bias, was used as the standardized effect size [34]. Subgroups based on mHealth type, sample size, duration of intervention, geographic distribution, comparator, setting, and sex proportion were subjected to parallel meta-regression in order to identify the sources of heterogeneity in the patient primary outcomes. Subgroup cutoffs for sample size and sex proportion were defined using the median values: 106 (IQR 102; range 72.5-174.5) patients and 62.75% (IQR 20.63; range 57.15-77.76) male. Analyses were performed with R software (version 4.2.2; R Foundation for Statistical Computing) along with the packages *meta* (version 7.0.0) and *metafor* (version 4.6.0) [35-37].

## Results

### Search Results

**Figure 1.**
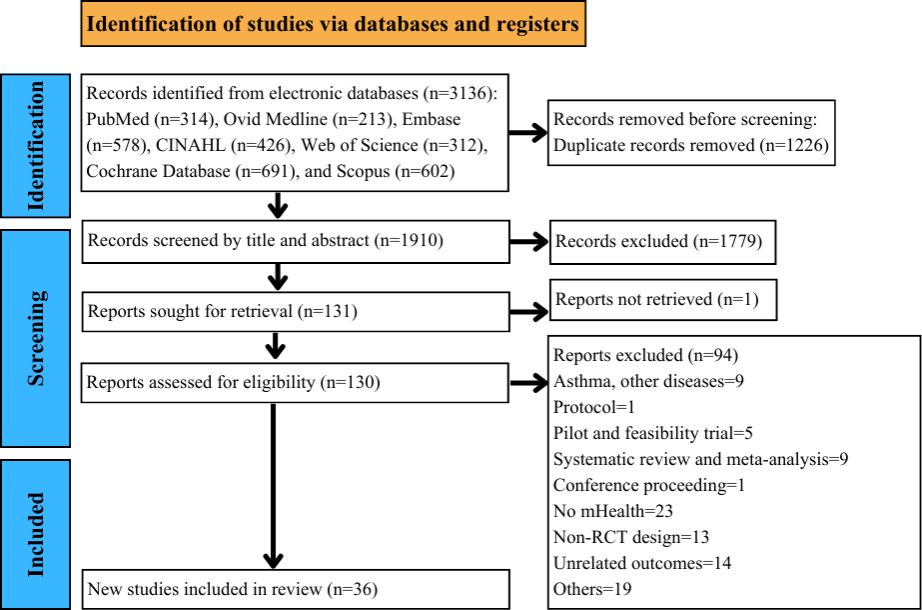
PRISMA (Preferred Reporting Items for Systematic Reviews and Meta-Analyses) 2020 flow diagram. mHealth: mobile health; RCT: randomized controlled trial.

The initial search of the electronic databases yielded 3136 articles. After deleting duplicates and screening the titles and abstracts, 130 full-text articles were assessed, resulting in 36 studies [38-73] deemed eligible to be included in the review (see Figure 1).

### Study Characteristics

**Figure 2.**
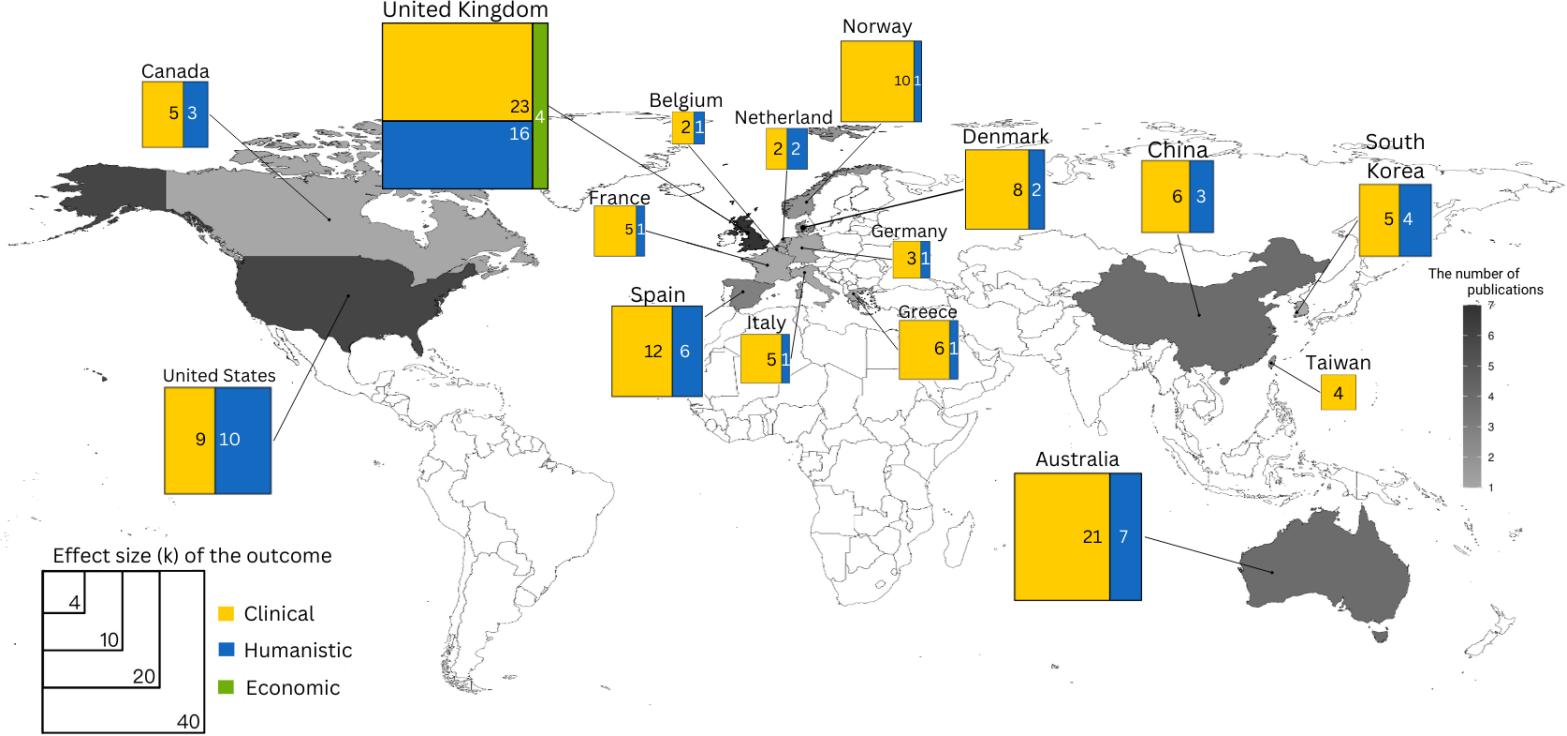
Distribution of chronic obstructive pulmonary disease mobile health studies based on the region and reported outcomes.

This systematic review included 36 trials. Figure 2 shows the geographic distribution of the studies, with 19 studies from Europe [38-56], 10 studies from Asia and Australia [57-66], and 7 studies from the United States and Canada [67-73]. The most significant number of studies was published in 2017 (8 studies [42-44,55,59,64,65,68]), and the least was in 2019 (1 study [41]). There were 5606 patients involved in these studies, 3774 (67%) of whom were men. Most RCTs were conducted on a relatively small number of patients, with 26 studies [39-42,47,48,50,51,53-59,61-66,68-70,72,73] recruiting fewer than 100 patients in each group. Mainly, the experiment settings were programmed as home-based services encompassing 31/36 studies (86%; see Figure S1 in Multimedia Appendix 2).

### Type of mHealth Interventions

Types of mHealth interventions are calculated in Figure S1 in Multimedia Appendix 2. From 36 studies, the majority (22/36, 61%) of studies used web-based and computer-based interventions, such as websites, video conferences, social media, and electronic diaries, which were connected to a television or tablet [38,41-45,49-52,55,57-59,64,67-73]. The clinical devices used included a pulse oximeter, pedometer, spirometer, pulse wave monitor, and a biometric sensor equipped with an alert system or electronic health records. Most studies were categorized as mHealth-based self-management with educational and motivational materials, physical activities, rehabilitation programs, symptom recording, feedback, and support. A detailed summary of intervention components (eg, education, monitoring, and feedback) for each study is available in the Table S1 in Multimedia Appendix 2 [38-64,66-73]. Most studies (31/36, 86%) used control groups that received usual or conventional care [38,40-43,70,71,73], with slight differences in whether a study applied PR or not (see Figure S1 in Multimedia Appendix 2).

### Involvement of Health Care Professionals

Health care providers played roles in delivering, assessing, and evaluating the self-management programs. Overall, 13 of 36 studies (36%) reviewed were delivered by physiotherapists [38-40,42,50,51,53,55,56,59,61,64-66], while proportions of treatment delivered by physicians [43-45,52,67-69,71,73] and nurses [41,46,54,57,58,60,62,70] were almost the same at 9/36 studies (25%; Figure S1 in Multimedia Appendix 2). Only 6 studies involved health care providers’ in-home visits or monitoring [43,45,48,52,59,65], while 9 studies included smoking cessation programs [42,43,46,47,59,62,63,72,73] and 9 studies focused on breathing techniques [42,43,49,54,57,59,61,70,73]. Most studies were categorized as PR and consisted of physical exercises and walking tests, inhaler use, health coaching, and education. A total of 29 trials provided patients the ability to enter data related to COPD symptoms, clinical signs, and amount of exercise activities by themselves or receive feedback from health care professionals or educators [39-41,43-49,51-53,55,58-61,63-73]. Detailed characteristics of the included studies are listed in Table 1.

**Table 1.** Characteristics of included randomized controlled trials (n=36).

Author, year	Country	Intervention	Duration (intervention; study)	Sample size, n		Study outcomes (ECHO^a^)
				IG^b^	CG^c^	
Web-based and computer-based programs						
Arbillaga-Etxarri et al [38], 2018	Spain	Web-based exercise via phone call and text message.	12 months; 12 months	132	148	Exercise capacity, exacerbation, QoL^d^, and dyspnea.
Benzo et al [70], 2021	The United States	Web-based PR^e^ via tablet, activity monitor, and pulse oximeter.	2 months; 6 months	72	74	Adherence, QoL, and self-management.
Benzo et al [71], 2022	The United States	Web-based PR via tablet, activity monitor, and pulse oximeter.	3 months; 3 months	188	187	QoL, self-management, daily physical activity, anxiety, and dyspnea.
Boer et al [41], 2019	The Netherlands	Web-based program with a touchscreen mobile phone.	12 months; 12 months	43	44	Exacerbation, QoL, and self-efficacy.
Bourne et al [42], 2017	The United Kingdom	Web-based PR.	1.5 months; 1.5 months	64	26	Exercise capacity, QoL, and dyspnea.
Chan et al [57], 2016	Taiwan	Computer-based breathing technique education.	3 months; 3 months	36	35	Self-efficacy and QoL.
Farmer et al [43], 2017	The United Kingdom	Computer-based program with Bluetooth-enabled pulse oximeter and videos.	12 months; 12 months	110	56	QoL, hospitalization, death, exacerbation, and cost.
Ho et al [58], 2016	Taiwan	Telemonitoring program with clinical devices and an online diary.	2 months; 6 months	53	53	Rehospitalization
Kessler et al [49], 2018	France	Web-based program and telephone.	12 months; 12 months	157	162	Hospitalization, exacerbation, exercise capacity, and QoL.
Moy et al [67], 2016	The United States	Web-based walking program with an automated pedometer.	4 months; 12 months	154	84	QoL, daily steps, and dyspnea.
Rixon et al [44], 2017	The United Kingdom	Home-based telemonitoring with clinical devices.	4 months; 12 months	334	244	QoL
Robinson et al [72], 2021	The United States	Web-based self-management and a pedometer.	6 months; 6 months	75	78	Exercise capacity, physical activity, QoL, dyspnea, and knowledge.
Saleh et al [50], 2023	Norway	Telemedicine video consultation via tablet with a web camera and microphone.	2 weeks; 12 months	57	57	Readmission, QoL, anxiety, and depression.
Stamenova et al [73], 2020	Canada	Computer-based self-monitoring program with emails, calls, and clinical devices.	6 months; 6 months	41	40	Self-management, QoL, knowledge, exacerbation, and hospitalization.
Tsai et al [64], 2017	Australia	Home-based real-time telerehabilitation using videoconferencing software.	2 months; 2 months	19	17	Lung function, exercise capacity, QoL, and dyspnea.
Vasilopoulou et al [55], 2017	Greece	Tablet and web-based platform.	2 months; 14 months	47	50	Lung function and functional capacity assessment, physical activity, QoL, and adherence.
Vianello et al [52], 2016	Italy	Home-based telehealth with alarm, website, and phone calls.	12 months; 12 months	230	104	QoL, hospitalization, and death.
Walker et al [45], 2018	The United Kingdom	Computer-based telemonitoring and an electronic diary.	9 months; 9 months	154	158	Hospitalization, exacerbation, and QoL.
Wan et al [68], 2017	The United States	Web-based educational program, online community forum, and pedometers	3 months; 3 months	57	52	Daily steps, exercise capacity, exercise self-efficacy, QoL, and dyspnea knowledge.
Wan et al [69], 2020	The United States	Web-based educational program, online community forum, and pedometers.	3 months; 15 months	57	52	Acute exacerbations, daily steps, exercise capacity, exercise self-efficacy, and QoL.
Wang et al [59], 2017	China	Web-based coaching program with electronic health records and messages.	12 months; 12 months	55	65	Lung function, QoL, dyspnea, and exercise capacity.
Zanaboni et al [51], 2023	Norway, Denmark, and Australia	Computer-based exercise training at home with videoconference supervision.	24 months; 24 months	40	40	Hospitalization and ED^f^ presentation, exercise capacity, dyspnea, QoL, anxiety and depression, and self-efficacy
Telephone-based program						
Holland et al [65], 2017	Australia	Home-based PR with structured phone calls.	2 months; 12 months	80	86	Exercise capacity, QoL, and dyspnea.
Jolly et al [66], 2018	The United Kingdom	Telephone-based coaching session with written materials, a pedometer, and a diary.	12 months; 12 months	289	288	QoL, dyspnea, self-efficacy, and hospitalization.
Varas et al [39], 2018	Spain	Telephone-based exercise program with a pedometer and a diary.	2 months; 12 months	21	19	Daily steps, exercise capacity, QoL, dyspnea, and number of exacerbations.
Wootton et al [66], 2018	Australia	Telephone-based walking program, pedometers, and a diary.	2 months; 14 months	49	46	QoL and exercise capacity.
Smartphone app–based program						
Bi [60], 2021	China	Instant communication platform with education material and voice conference	3 months; 3 months	100	100	Exercise frequency, QoL, and FEV_1_^i^%
Cerdán-De-las-Heras et al [53], 2022	Denmark	Mobile app with biometric sensor, video, e-learning packages, and physical training regimens.	2 months; 8 months	27	27	Exercise capacity, QoL, anxiety, FEV_1_, and FVC^h^.
Crooks et al [47], 2020	The United Kingdom	Online application with education, self-monitoring, and self-management functions.	3 months; 3 months	29	31	QoL, self-efficacy, and exacerbation
Jiang et al [61], 2020	China	Mobile app-based PR with modules and chat features.	3 months; 6 months	53	53	QoL, self-efficacy, and dyspnea.
Jimenez-Reguera [40], 2020	Spain	Mobile app-based program with education and online support aid.	12 months; 12 months	17	19	Treatment adherence, QoL, and exercise capacity.
Loeckx et al [56], 2023	Belgium	Semiautomated coaching application and step counter.	6 months; 12 months	37	36	Physical activity, dyspnea, and QoL.
North et al [48], 2020	The United Kingdom	Digital application with education, PR program, video, and environmental alerts.	3 months; 3 months	20	21	QoL, anxiety and depression, hospitalization, and dyspnea.
Park et al [63], 2020	South Korea	A smartphone app-based program with a pedometer, recorder, and video clips.	6 months; 6 months	22	20	Dyspnea, exercise capacity, QoL, self-efficacy, and hospitalization.
Spielmanns et al [54], 2023	Germany and Switzerland	App-based exercise training program and regular telephone calls	6 months; 6 months	33	34	Daily steps, exercise capacity, QoL, health status, and exacerbation.
Wang et al [62], 2021	China	Mobile app-based program with modules	12 months; 12 months	39	39	QoL and self-management behavior.

a ECHO: economic-clinical-humanistic outcome

b IG: intervention group.

c CG: control group.

d QoL: quality of life.

e PR: pulmonary rehabilitation.

f ED: emergency department.

g FEV1: forced expiratory volume in 1 second.

h FVC: forced vital capacity.

**Figure 3.**
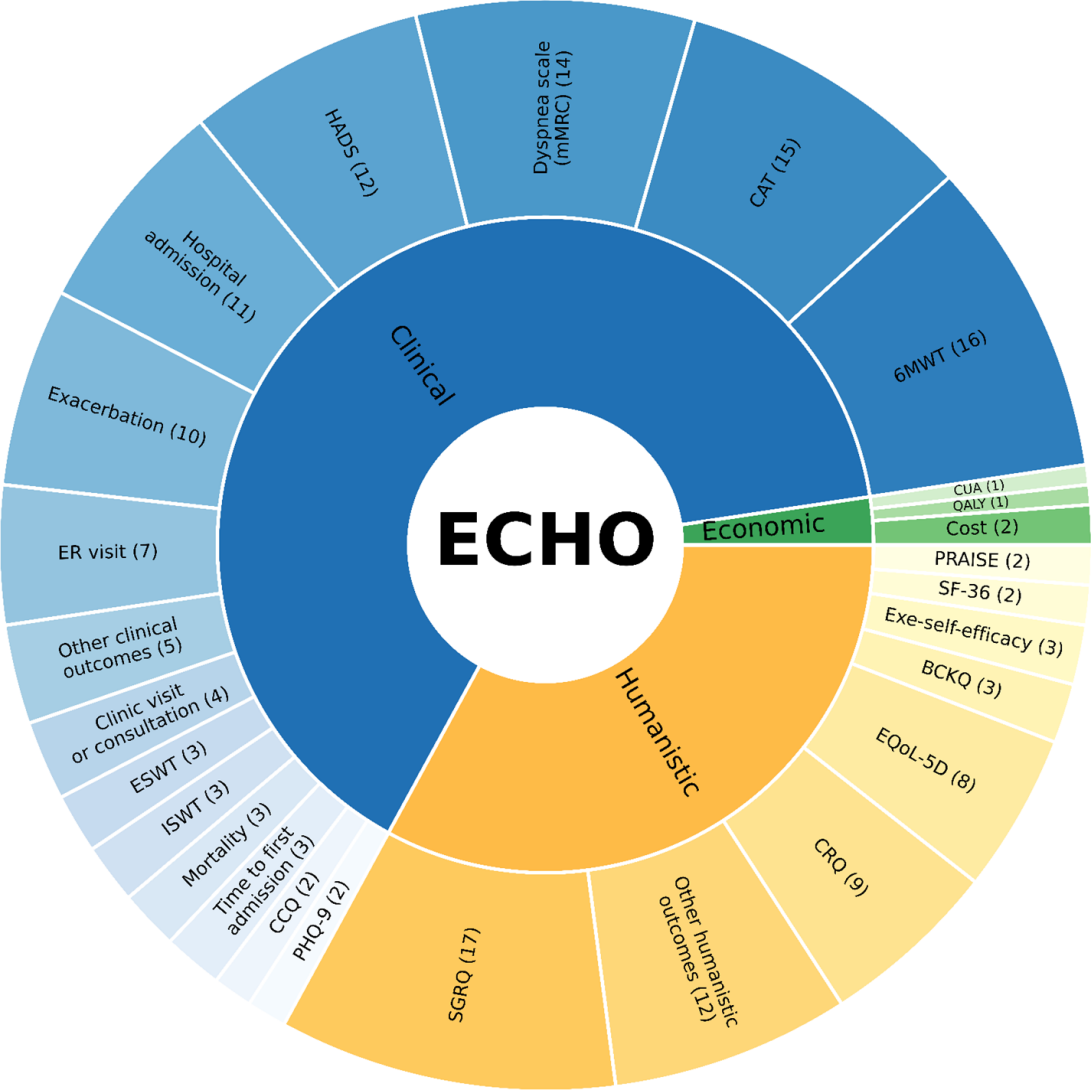
Analysis of outcomes of included studies based on the ECHO model. Other clinical outcomes include BDI-2 (Beck Depression Inventory-II), COTE (COPD-specific Comorbidity Test), number of exacerbation-free, STAI-6 (Brief State Trait Anxiety Inventory), and UCSD SOBQ (University of California, San Diego Shortness of Breath Questionnaire). Other humanistic outcomes include BMQ (Belief on Medication Question naire), BPAQ (Baecke Physical Activity Questionnaire), CAP-FISIO (a respiratory physiotherapy adherence self-report), Exa-Self-efficacy (Exacerbation-related self-efficacy), MARS (Medication Adherence Rating Scale), MLHRQ (Minnesota Living with Heart Failure Questionnaire), Morisky Green, PIH (Partners in Health, Self-Manage ment), SCBI (Self-Care Behavior Inventory), SEMCD (Self-Efficacy for Managing Chronic Disease 6-Item Scale), SF-12 (12-Item Short Form Health Survey), and Stanford SES (Stanford Self-efficacy Scale). BCKQ: Bristol COPD Knowledge Questionnaire; CAT: COPD Assessment Test; CCQ: Clinical COPD Questionnaire; CRQ: Chronic Respiratory Disease Questionnaire; CUA: cost-utility analysis; ESWT: endurance shuttle walking test; Exe-self efficacy: exercise self-efficacy; HADS: Hospital Anxiety and Depression Scale; ISWT: incremental shuttle walk test; PHQ-9: Patient Health Questionnaire scores for each of the 9 Diagnostic and Statistical Manual of Mental Disorders IV criteria; SF-36: 36-Item Short Form Health Survey.

Figure 3 presents the diversity of outcomes and instruments in studies based on the ECHO model. The majority of studies reported the clinical domain as the primary or secondary outcome, which included clinical outcomes (eg, hospital admission, clinic or ER visits, and mortality), symptom detection (eg, COPD Assessment Test, dyspnea scale, Hospital Anxiety and Depression Scale, and exacerbation), and others. All reported humanistic outcomes were derived from questionnaire responses or distinctive scales to assess various parameters (eg, self-efficacy, QoL, and adherence). Only 2 articles revealed economic outcomes. The numbers in the brackets illustrate the number of articles that used these instruments/outcomes, and detailed outcomes are reported in Tables S3-S5 in Multimedia Appendix 3 [38-64,66-73].

### Risk-of-Bias and Publication Bias Assessment

**Figure 4.**
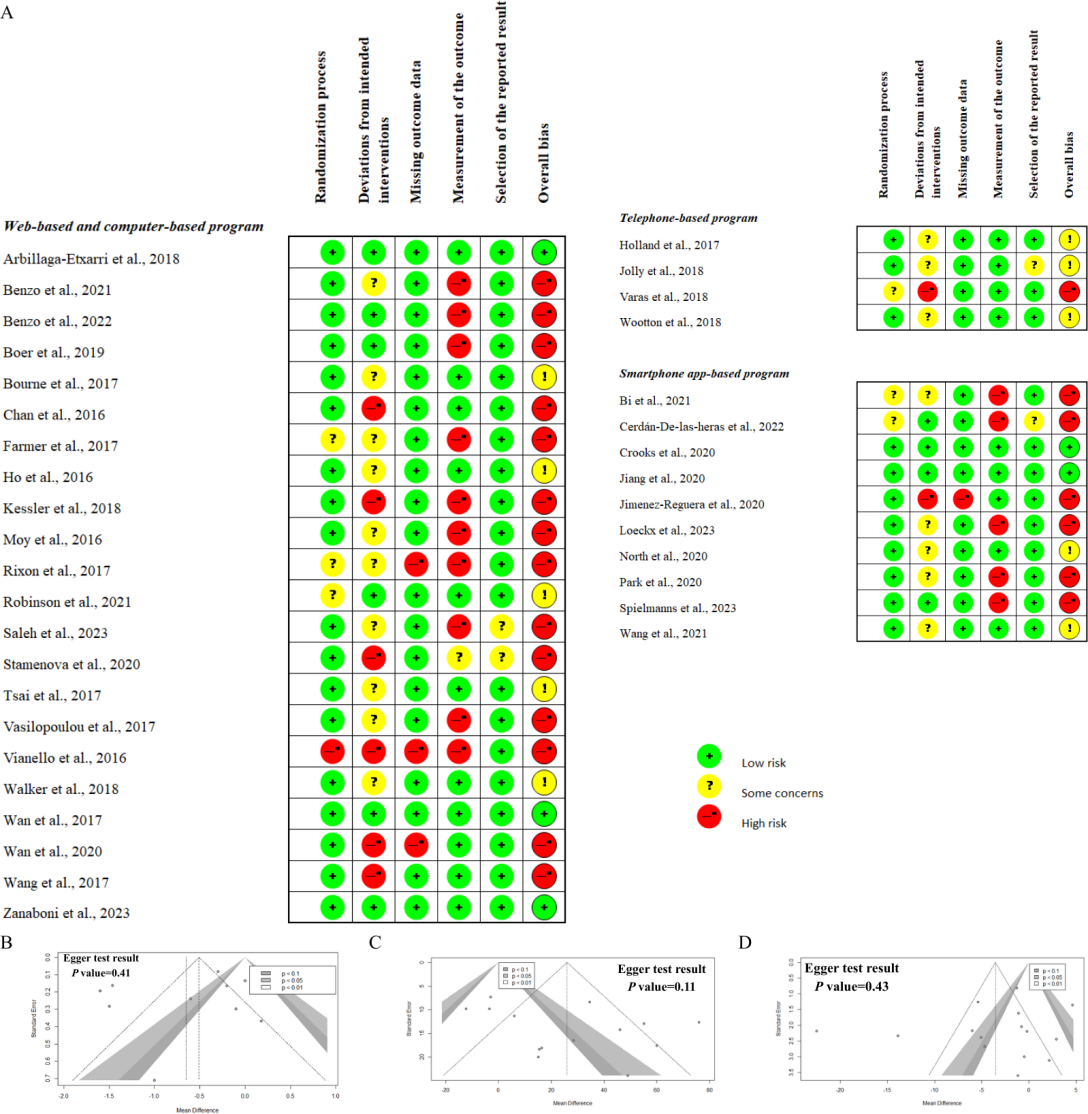
Risk-of-bias and publication bias results. (A) Risk-of-bias assessment. (B) Publication bias of studies reporting the modified Medical Research Council (mMRC) Dyspnea Scale. (C) Publication bias of studies reporting the 6-minute walking test (6MWT). (D) Publication bias of studies reporting St. George Respiratory Questionnaire (SGRQ) total scores [38-73].

Figure 4 demonstrates the potential risk-of-bias evaluation. A total of 5 studies were categorized as having low risk, 10 as having some concerns, and 21 studies as having a high risk of bias. The most common finding for a high risk of bias was in the measurement of the outcome and deviations from the domain of the intended interventions. This indicated the lack of blinding patients, caregivers, the people delivering the interventions, and the assessors involved. Publication bias is evident in a funnel plot, and the Egger test of studies included in the analysis of primary outcomes showed no significant evidence, supporting the absence of publication bias (see Figure 4B-D).

### Outcomes Measures

#### Primary Outcome

**Figure 5.**
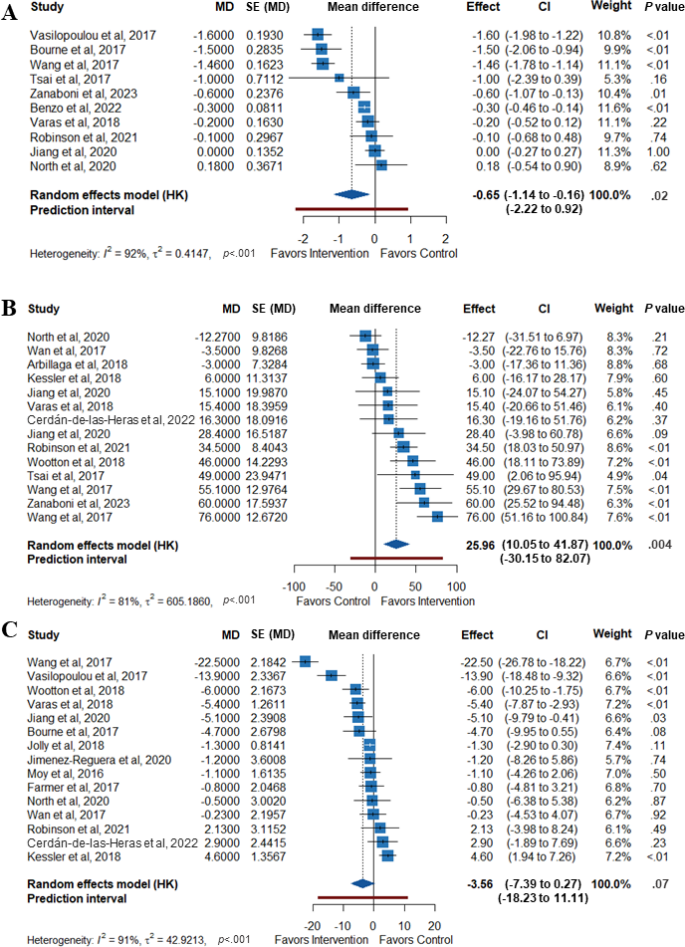
Forest plot of studies to observe the effectiveness of mobile health interventions on the (A) mMRC (modified Medical Research Council) Dyspnea Scale [39,42,48,51,55,59,61,64,71,72], (B) 6MWT (modified Medical Research Council) [38,40,42,43,46,48,49,53,55,59,61,66-68,72], and (C) the total scores of the SGRQ (St. George Respiratory Questionnaire) [39,40,42,43,46,48,49,53,55,59,61,66-68,72].

Figure 5 shows the results from the meta-analysis of dyspnea symptoms by the mMRC Dyspnea Scale, exercise capacity by the 6MWT, and QoL by the SGRQ total score. The pooled mean differences in improvement of the mMRC Dyspnea Scale and exercise capacity (6MWT) among patients receiving mHealth interventions compared to control groups were −0.65 (95% CI −1.14 to −0.16; *P*=.02) and 25.96 m (95% CI 10.05 m to 41.87 m; *P*<.01), respectively, indicating a statistically significant effect of the intervention. There was no statistical difference in the total SGRQ scores between the groups (mean difference −3.56, 95% CI −7.39 to 0.27; *P*=.07).

Heterogeneity (*I*^2^) exceeded 50% across the studies for the primary outcomes, implying high variability in effect sizes. A summary of health-related outcome findings from the studies reported as the mean (SD) in both groups is described in Table S6 in Multimedia Appendix 4 [38-64,66-73]. Possible heterogeneity among studies might have been influenced by different types of mHealth used in the self-management programs, patient severity levels, sample sizes, and follow-up durations. Sensitivity analysis was conducted using the leave-one-out method. There are no abnormal values discovered for the mMRC dyspnea scale and 6MWT score (see Figures S2-S4 in Multimedia Appendix 5 [38-64,66-73]). However, the SGRQ total score reveals varying results, suggesting that the overall effect estimate was sensitive to the inclusion of Cerdán-De-las-Heras et al [53] with a *P=*.049 and Kessler et al [49] with a *P=*.037. The results changed to statistically significant when these studies were eliminated. The revised forest plots, excluding this study, are provided in Figures S5-S7 in Multimedia Appendix 5 [38-64,66-73].

**Figure 6.**
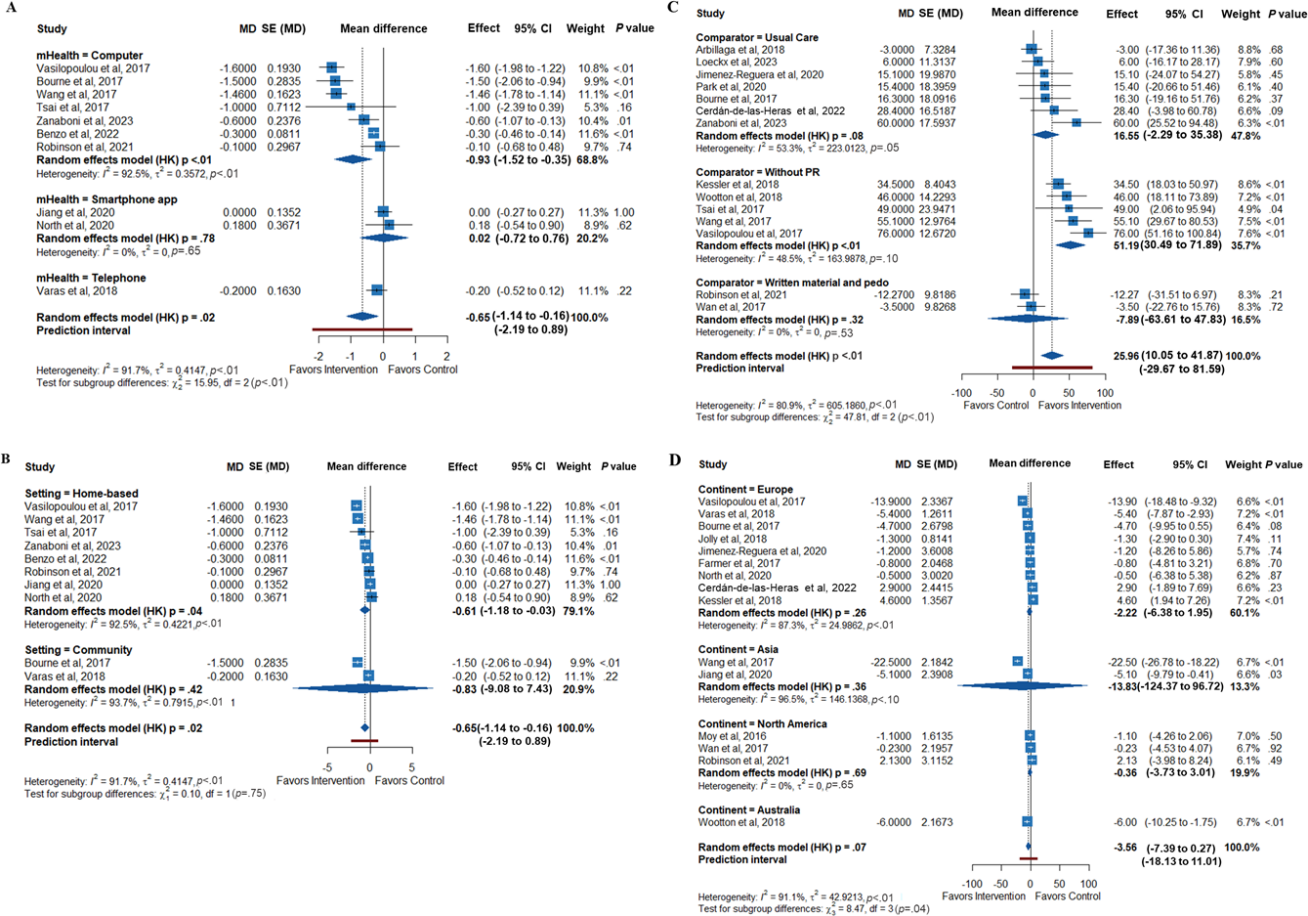
Forest plot of subgroup analysis (A) by the type of mobile health on mMRC (modified Medical Research Council) Dyspnea Scale [39,42,48,51,55,59,61,64,71,72], (B) by the setting on mMRC Dyspnea Scale [39,42,48,51,55,59,61,64,71,72], (C) by the comparator on 6MWT (6-minute walking test) [38,40,42,49,51,53,55,56,59,63,64,66,68,72], and (D) by the continent on SGRQ (St. George Respiratory Questionnaire) total score [39,40,42,43,46,48,49,53,55,59,61,66-68,72].

Subgroup studies indicated that mMRC might be linked to computer-based mHealth therapies, those without a PR comparator, and a home-based setting group. Meanwhile, 6MWT seemed to benefit from interventions in computer-based mHealth therapies, smaller sample sizes, shorter durations of intervention, studies conducted in Europe and Australia, home-based setting, without PR comparator, and studies with a lower male proportion. Additionally, a significant improvement in quality of life (SGRQ) was observed in the shorter-duration and community setting subgroup. All detailed statistical analysis results can be found in the Figures S8-S28 in Multimedia Appendix 5 [38-64,66-73]. The origins of heterogeneity were further investigated using meta-regression analysis. The 6MWT was the only outcome showing significant differences across comparator subgroups (Table 2 and Figure 6), whereas the mHealth type showed a borderline effect on the mMRC dyspnea scale. The complete results of the subgroup analysis can be found in Table S7 in Multimedia Appendix 5 [38-64,66-73].

**Table 2.** Summary of main findings and subgroup analyses on the effectiveness of mobile health interventions for modified Medical Research Council Dyspnea Scale, 6-minute walking test, and St. George Respiratory Questionnaire total score.

Subgroup	Number of studies	Mean difference (95% CI)		Main outcome, *P* value	Heterogeneity		Meta regression, *P* value	Effects model
					*I*^2^ (%)	*P* value		
mMRC^k^ Dyspnea Scale	10 [39,42,48,51,55,59,61,64,71,72]	−0.65 (−1.14 to −0.16)		.02^b^	92	<.001	/^c^	Random
Type of mHealth intervention								
Computer	7 [42,51,55,59,64,71,72]	−0.93 (−1.52 to −0.35)		.008^b^	93	<.001	.09^d^	Random
Smartphone app	2 [48,61]	0.02 (−0.72 to 0.76)		.78	0^e^	.65		Random
Telephone	1 [39]	−0.2 (−0.52 to 0.12)		.22	/	/	/	—^f^
Setting								
Home-based	8 [48,51,55,59,61,64,71,72]	−0.61 (−1.18 to −0.03)		.04^b^	92.5	<.001	.71	Random
Community	2 [39,42]	−0.83 (−9.08 to 7.43)		.42	93.7	<.001		Random
Hospital-based	/	/	/	/	/	/		—^f^
6MWT^g^ (meters)	14 [38,40,42,49,51,53,55,56,59,63,64,66,68,72]	25.96 (10.05 to 41.87)		.004	81	<.001	/	Random
Comparator								
Usual care	7 [38,40,42,51,53,56,63]	16.56 (−2.29 to 35.38)		.08	53	.05	<.001^h^	Random
Without PR^i^	5 [49,55,59,64,66]	51.19 (30.49 to 71.89)		.002^b^	49^e^	.10		Random
Written material and a pedometer	2 [68,72]	−7.89 (−63.61 to 47.83)		.32	0^e^	.53		Random
SGRQ^j^ total score	15 [39,40,42,43,46,48,49,53,55,59,61,66-68,72]	−3.56 (−7.39 to 0.27)		.07	91	<.001	/	Random
Continent								
Asia	2 [59,61]	−13.83 (−124.37 to 96.72)		.36	97	<.001	.05^d^	Random
Australia	1 [66]	−6.00 (−10.25 to −1.75)		.006	/	/		—
Europe	9 [39,40,42,43,46,48,49,53,55]	−2.22 (−6.38 to 1.95)		.26	87	<.001		Random
North America	3 [67,68,72]	−0.36 (−3.73 to 3.01)		.69	0^e^	.65		Random

a mMRC: modified Medical Research Council.

b The *P* value, along with the CI, indicates a statistically significant result.

c Indicates that meta-regression was not performed.

d The meta-regression results indicate borderline significance for both analyses: *P*=.09 suggests that the type of mHealth intervention may moderate its effectiveness on the mMRC Dyspnea Scale, while *P*=.05 indicates that geographic location (grouped by continent) may influence the intervention’s effectiveness on St. George Respiratory Questionnaire quality of life.

e Heterogeneity within this subgroup was low, with an *I*² value ≤50%

f Not available.

g 6MWT: 6-minute walking test.

h Meta-regression results indicate that the comparator type significantly moderated the effect of mHealth interventions on the 6MWT outcome (*P*<.001).

i PR: pulmonary rehabilitation.

j SGRQ: St. George Respiratory Questionnaire.

#### Secondary Outcomes

**Figure 7.**
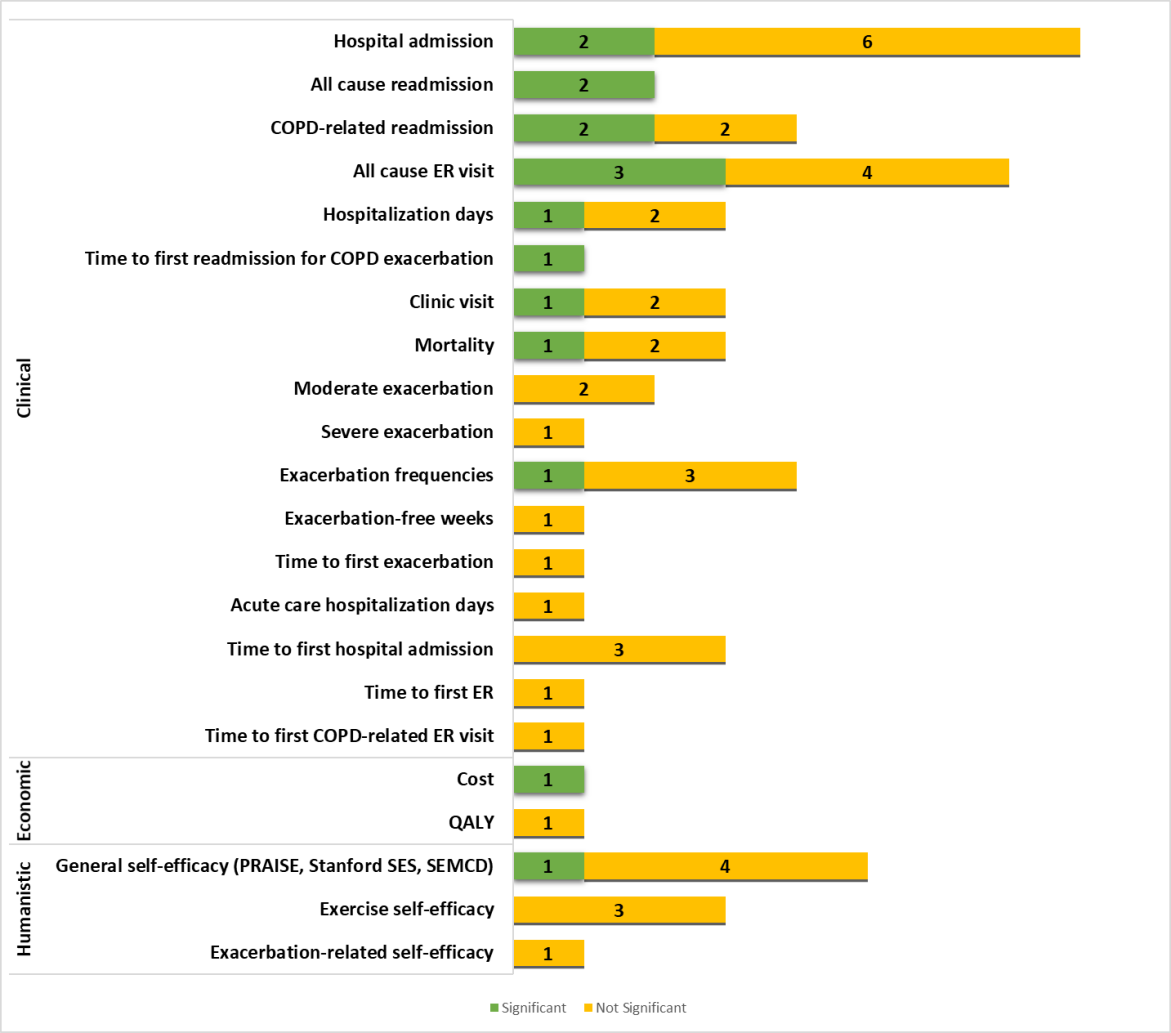
Effects of mobile health interventions on other economic, clinical, and humanistic outcomes. COPD: chronic obstructive pulmonary disease; ER: emergency room; PRAISE: Pulmonary Rehabilitation Adapted Index of Self-Efficacy; QALY: quality-adjusted life year; SEMCD: Self-Efficacy for Managing Chronic Disease 6-Item Scale; Stanford SES: Stanford Self-Efficacy Scale.

Figure 7 shows the secondary economic, clinical, and humanistic outcomes, which are detailed in Tables S3-S5 in Multimedia Appendix 3 [38-64,66-73].

##### Economic Secondary Outcomes

A total of 2 economic studies reported costs and QALYs [43,45]. Walker et al [45] reported that the mean cost per patient in the intervention group was lower than in the control group, except for the severe or very severe COPD subgroup. In a comparison of the intervention with the control group, there was a possible statistically significant decrease in the mean cost per patient (€3547 vs €4831, US $4118.4 VS US $5609.24; *P*=.01), but no statistically significant difference in QALYs (0.485 vs 0.491; *P*=.73) [45].

##### Clinical Secondary Outcomes

Figure 7 shows that 11 research articles from the clinical domain reported hospital admissions using various scale measures [43,45,46,49-52,55,58,63,73]. The majority of the assessment scales applied (7 outcomes) generated a significant reduction in the mHealth-treated group compared to the control group [45,51,52,55,58]. mHealth also prolonged the time to first readmission for COPD exacerbation [58]. A total of 3 studies reported mortality, and only 1 showed a significant decrease in the mortality rate [49]. Among 12 studies reporting exacerbation [38,39,41,43,45,47-49,54-56,73], only a single study showed a significant improvement in exacerbation frequencies for patients who used telerehabilitation compared to usual treatment [55].

##### Humanistic Secondary Outcomes

A total of 9 studies reported data relating to self-efficacy using the general self-efficacy index, including the PRAISE [57,64,65], Stanford Self-Efficacy Scale [46], Self-Efficacy for Managing Chronic Disease 6-Item Scale [63], exercise self-efficacy index [61,68,69], and exacerbation-related self-efficacy [41]. Only a single study reported a significant improvement in the PRAISE score in the mHealth treatment group compared to the control [64].

## Discussion

### Principal Findings

This systematic review included 36 RCTs from 5 databases evaluating mHealth interventions’ impact on COPD, including clinical, humanistic, and economic outcomes. It offers a thorough perspective by integrating studies from various geographical regions, clinical settings, forms of intervention, types of control groups, the health care provider involvement, and outcome metrics. mHealth interventions demonstrated promising results in supporting self-management among patients with COPD. They enhance symptom control and improve exercise capacity, which are key targets in PR. Improvements in other clinical domains were also observed, but their economic and humanistic impacts remain comparatively limited. However, the pooled analysis for QoL did not demonstrate statistically significant effects, although some individual study results showed potential. These findings highlight clinical benefits, including better access, early symptom detection, fewer hospitalizations, more sustained exercise, and rehabilitation effects, despite variations in delivery methods, study sizes, and mHealth tools.

This review offers a key strength by providing comprehensive data on the global distribution of research on mHealth interventions and highlighting inequalities in digital infrastructure for patients with COPD. Most of the studies were from Europe, the United States, Canada, and, to a lesser extent, Asia and Australia, indicating that the findings are broadly applicable to high-income health care settings, with limited representation from Africa and other regions of Asia [74-76]. While evidence demonstrated the emerging potential of digital health interventions, the primary challenge in resource-limited developing countries is obtaining sufficient funding for their implementation and long-term sustainability [77]. Furthermore, there are inequalities in digital infrastructure, a lack of technical expertise, undeveloped regulatory frameworks, and limited implementation capacity, encompassing technology ownership, privacy, and security concerns. These are significant obstacles to adopting digital health in less-developed countries [78,79]. Efforts to address these challenges should align with the WHO’s global strategy for digital health, which optimizes data use to achieve better well-being and sustainable development goals related to health [9].

This meta-analysis revealed a majority of positive outcomes observed in the clinical domain. The results appear to have clinical relevance when compared to the established minimal clinically important differences for the mMRC Dyspnea Scale (-0.5 to -1.0 points) and 6MWT (25-33 m) [80-83]. While previous studies reported contradictory results regarding dyspnea symptoms and exercise capacity [14,16,19,20,24], the findings from this meta-analysis demonstrated clear, statistically significant, and clinically meaningful improvements in both measures. Symptom reduction was identified as a primary treatment goal in the latest GOLD report, with PR recommended as a nonpharmacological intervention to enhance exercise capacity [8]. These encouraging findings may support the provision of mHealth-facilitated PR to increase patient access, capacity, uptake, and clinical effectiveness [84-87].

This review also raised the possibility of favorable critical clinical outcomes, including decreased hospital admission rates, prolonged times to first readmission, reduced mortality rates, and lower exacerbation frequencies, as evidenced by several studies [45,49,51,52,55,58]. At the same time, one of the included studies reported a potential reduction in health care costs [45]. This observation is in line with the vision outlined in the WHO global strategy on digital health, which emphasizes the potential of digitalization to enhance the efficiency and cost-effectiveness in the health sector, while supporting innovative business models in service delivery [9]. Inocencio et al [88] and Stecher et al [89] indicate that cost reductions result from mechanisms such as remote monitoring, timely feedback, therapy optimization, improved adherence, lower hospital admission costs, and exacerbation events. However, robust evidence on the clinical and economic impacts of mHealth care use remains scarce and of low quality, underscoring the need for more rigorous and comprehensive research. While the findings appear favorable, they must be viewed in light of the study design weaknesses, particularly the high risk of bias in most included trials.

The GOLD report recognizes self-management as a strategy to improve QoL, with technological advancements offering benefits to both patients and health care professionals. Individual studies included in the analysis demonstrated that patient adherence and the content of the intervention program influenced the effectiveness of the mHealth-based self-management program. These are noteworthy factors for maintaining short-term training advantages over time [39,55,59,66]. Building on earlier research by Shaw et al [18] and Janjua et al [90], which reported uncertain outcomes regarding self-efficacy, our review presents early evidence that PRAISE scores for self-efficacy may improve, and this deserves further investigation [64]. According to the study’s pooled analysis, the SGRQ total score did not show a significant effect of mHealth treatments for self-management in patients with COPD. Likewise, the result did not meet the minimal clinically important differences for the SGRQ total score, which is 4 units [91,92]. It is generally challenging to show a substantial improvement in SGRQ scores, as earlier reviews pointed out [14,19,24,93]. When considerable effects of QoL are seen, they are typically documented in studies that used a variety of questionnaires [94] and have brief observation periods (≤6 months) [14]. As a self-reported tool, the SGRQ score is often influenced by baseline group characteristics and patient engagement with the intervention [14,43,46,67,68].

The emergence of digital health technologies in clinical practice has demonstrated impacts across the ECHO model, as reflected in numerous studies [45,75,94]. The COPD mHealth technologies clearly improved clinical outcomes, including mMRC and 6 MWT. Current mHealth evaluations in COPD lack robust economic data and show limited humanistic effects. Their multidimensional impact highlights the need for comprehensive outcome studies in the future. This reinforces the relevance of the ECHO model for real-world evaluation, as it captures benefits that extend beyond symptom reduction, including health care use, patient experience, and broader system-level value. Consistent with WHO’s Global Strategy on Digital Health, using ECHO-based approaches can strengthen decisions on policy adoption, reimbursement, and scale-up of COPD mHealth programs [9].

The study’s findings should be interpreted with caution. The sensitivity analysis revealed that the pooled SGRQ result was sensitive to the inclusion of individual studies, particularly Cerdán-De-las-Heras et al [53] and Kessler et al [49]. This indicates limited robustness of the findings. However, the initial SGRQ forest plot revealed a borderline significant effect (*P*=.07), with several individual studies showing promising trends in favor of the intervention. Furthermore, meta-regression revealed that comparator type significantly moderated 6MWT outcomes (*P*<.001), indicating its influence on observed exercise capacity effects. Most studies exhibited a high risk of bias, small sample sizes (<100), and variability in outcome measures, resulting in high heterogeneity that limited the generalizability of efficacy findings. Additionally, the majority of interventions were delivered over a relatively short duration (<6 months), which may have affected the ability to observe sustained clinical outcomes. The inability to blind patients, caregivers, and service providers as a natural aspect of digital health research [50] led to a high risk of bias in most of the studies included. In addition, the risk-of-bias assessment relies on partly subjective tools that depend heavily on the evaluator’s judgment. The instruments used across studies were also heterogeneous (eg, 36-Item Short Form Health Survey and EuroQol-5D to measure quality of life), limiting comparability of outcomes between studies. The study’s limitations also include the absence of mHealth-specific reporting standards such as the WHO-recommended mHealth Evidence Reporting and Assessment checklist [95], as well as the lack of assessment of app quality [96] and its impact on user health outcomes. In the future, this represents an opportunity to conduct studies that adhere to more specific mHealth reporting and evaluation standards. Overall, there was a lack of studies evaluating the broader impacts of mHealth on COPD, including medication adherence and cost-effectiveness [97-99].

The safety considerations in the use of mHealth self-management interventions must also be addressed. Several potential adverse effects of mHealth interventions include the misinterpretation of self-reported data, challenges related to privacy and data security, and the risk of overreliance on technology, which may delay emergency interventions [100-103]. Furthermore, excessive dependence on technology could negatively impact mental health and unintentionally strain the patient-provider relationship by reducing human interactions [104]. Therefore, while mHealth offers promising benefits, it is crucial to address these psychological and personal aspects in the design of mHealth interventions, ensuring the support, rather than replacement, of holistic and balanced health care practices. In addition, future studies are encouraged to explore long-term interventions to better understand the sustainable impact of mHealth in patients with COPD.

### Conclusions

Findings of this review align with the GOLD 2025 recommendation, suggesting that mHealth interventions can serve as supplementary resources in clinical practice [8]. Their practical implementation necessitates comprehensive patient education, adherence to ethical guidelines, maintenance of confidentiality, and acquisition of the patient’s informed consent. Further high-quality, large-scale research is needed to develop accessible mHealth tools that offer virtual education and active feedback, use standardized outcome measures, and are tailored to diverse age groups, disease severities, and socioeconomic backgrounds. Carefully designed studies are required to comprehensively evaluate the efficacy of mHealth across economic, clinical, and humanistic domains, while also assessing its long-term safety.

## Supplementary material

10.2196/74967 Multimedia Appendix 1 Search strategy.

10.2196/74967 Multimedia Appendix 2 Characteristics of studies using mobile health interventions for patients with chronic obstructive pulmonary disease.

10.2196/74967 Multimedia Appendix 3 Summary of health-related outcomes instruments.

10.2196/74967 Multimedia Appendix 4 Summary of the main findings for health outcomes.

10.2196/74967 Multimedia Appendix 5 The results of sensitivity and subgroup analysis.

10.2196/74967 Checklist 1 PRISMA (Preferred Reporting Items for Systematic reviews and Meta-Analyses) 2020 checklist.

## Abbreviations

6MWT: 6-minute walking test
COPD: chronic obstructive pulmonary disease
ECHO: economic, clinical, and humanistic outcomes
EQ-5D: EuroQol-5 Dimensions
GOLD: Global Initiative for Chronic Obstructive Lung Disease
MeSH: Medical Subject Headings
mHealth: mobile health
mMRC: modified Medical Research Council
PR: pulmonary rehabilitation
PRAISE: Pulmonary Rehabilitation Adapted Index of Self-Efficacy
PRISMA: Preferred Reporting Items for Systematic Reviews and Meta-Analyses
PROSPERO: International Prospective Register of Systematic Reviews
QALY: quality-adjusted life year
QoL: quality of life
RCT: randomized controlled trial
SGRQ: St. George Respiratory Questionnaire
WHO: World Health Organization
