# Supplementary material for: Effectiveness of Mobile Health–Based Self-Management Programs on Health-Related Outcomes in Patients With Chronic Obstructive Pulmonary Disease: Systematic Review and Meta-Analysis
Source: JMIR Mhealth Uhealth. 2025 Dec 29;13:e74967. doi: 10.2196/74967 (PMC12747663; doi:10.2196/74967)
Supplement: Multimedia Appendix 1 [file mhealth-v13-e74967-s001.docx]

**Table S1.**

| PubMed | | |
| --- | --- | --- |
| **Search** | **Query** | **Items found**  **20241001** |
| #1  MeSH | (((pulmonary disease, chronic obstructive[MeSH Terms]) OR (lung diseases, obstructive[MeSH Terms])) OR (pulmonary emphysema[MeSH Terms])) OR (Bronchitis, Chronic[MeSH Terms])  Filters: from 2015/1/1 - 2024/9/30 | 67604 |
| #2  Title/  Abstract | (((((((((((((Pulmonary Disease, Chronic Obstructive[Title/Abstract]) OR (Lung Diseases, Obstructive[Title/Abstract])) OR (COPD[Title/Abstract])) OR (Chronic Obstructive Pulmonary Disease[Title/Abstract])) OR (COAD[Title/Abstract])) OR (Chronic Obstructive Airway Disease[Title/Abstract])) OR (Chronic Obstructive Lung Disease[Title/Abstract])) OR (Airflow Obstruction*, Chronic[Title/Abstract])) OR (Chronic Airflow Obstruction*[Title/Abstract])) OR (Obstructive Lung Disease*[Title/Abstract])) OR (Obstructive Pulmonary Disease*[Title/Abstract])) OR (Pulmonary Disease*, Obstructive[Title/Abstract])) OR (Chronic Bronchitis[Title/Abstract])) OR (Pulmonary Emphysema[Title/Abstract])  Filters: from 2015/1/1 - 2024/9/30 | 51523 |
| #3 | #1 OR #2  Filters: from 2015/1/1 - 2024/9/30 | 91078 |
| #4  MeSH | "self-management"[MeSH Terms] OR "self care"[MeSH Terms] OR "self-control"[MeSH Terms]  Filters: from 2015/1/1 - 2024/9/30 | 34185 |
| #5  All fields | (((((Self manag*[All fields]) OR (Self management[All fields])) OR ((Self care[All fields]) OR (Selfcare[All fields])) ) OR (Self-control[All fields])) OR (Self-monitor*[All fields]))  Filters: from 2015/1/1 - 2024/9/30 | 274820 |
| #6 | #4 OR #5  Filters: from 2015/1/1 - 2024/9/30 | 274820 |
| #7  MeSH | (((((((((Telemedicine[MeSH Terms]) OR (Mobile Applications[MeSH Terms])) OR (Wearable Electronic Devices[MeSH Terms])) OR (Electronic Mail[MeSH Terms])) OR (Telephone[MeSH Terms])) OR (Cell Phone[MeSH Terms])) OR (Text Messaging[MeSH Terms])) OR (Computers[MeSH Terms])) OR (Computers, Handheld[MeSH Terms])) OR (Smartphone[MeSH Terms])  Filters: from 2015/1/1 - 2024/9/30 | 78700 |
| #8  Title/Abstract | (((((((((((((((((((((((((((((((((((((((((((((((((((((((((((Tele[Title/Abstract]) OR (Mobile[Title/Abstract])) OR (Mobile Health[Title/Abstract])) OR (mHealth[Title/Abstract] OR m-Health[Title/Abstract])) OR (Telehealth[Title/Abstract] OR Tele-health[Title/Abstract])) OR (eHealth[Title/Abstract] OR e-Health[Title/Abstract])) OR (Digital Health[Title/Abstract])) OR (Telecoaching[Title/Abstract])) OR ((Telemedicine[Title/Abstract]) OR (Tele-medicine[Title/Abstract]))) OR (Physiologic* Monitoring[Title/Abstract])) OR ((Monitoring, Patient[Title/Abstract]) OR (Patient Monitoring[Title/Abstract]))) OR ((telemonitor*[Title/Abstract]) OR (tele-monitor*[Title/Abstract]))) OR (home monitor*[Title/Abstract])) OR (distant monitor*[Title/Abstract])) OR (remote monitor*[Title/Abstract])) OR (telehomecare[Title/Abstract])) OR (telecare[Title/Abstract])) OR ((tele-manage*[Title/Abstract]) OR (telemanage*[Title/Abstract]))) OR (internet[Title/Abstract])) OR (digital[Title/Abstract])) OR (online[Title/Abstract])) OR (Web[Title/Abstract])) OR (technolog*[Title/Abstract])) OR (electronic*[Title/Abstract])) OR ((web-based[Title/Abstract]) OR (webbased[Title/Abstract]))) OR (internet-mediated[Title/Abstract])) OR (Phone*[Title/Abstract])) OR (Telephone*[Title/Abstract])) OR (Cell phone*[Title/Abstract])) OR (Mobile phone*[Title/Abstract])) OR (Smartphone*[Title/Abstract])) OR (iPhone*[Title/Abstract])) OR (iPad*[Title/Abstract])) OR (Computer*[Title/Abstract])) OR (Handheld Computer*[Title/Abstract])) OR (Personal Digital Assistant*[Title/Abstract])) OR (PDA Computer*[Title/Abstract])) OR (Pocket PC*[Title/Abstract])) OR (Tablet Computer*[Title/Abstract])) OR (Tablet*[Title/Abstract])) OR (App[Title/Abstract] OR Apps[Title/Abstract])) OR (Application*[Title/Abstract])) OR (Mobile app*[Title/Abstract])) OR (Mobile application*[Title/Abstract])) OR (Mobile phone app*[Title/Abstract])) OR (Mobile phone application*[Title/Abstract])) OR (Smartphone app*[Title/Abstract])) OR (Smartphone application*[Title/Abstract])) OR (Mobile health app*[Title/Abstract])) OR (Mobile health application*[Title/Abstract])) OR (mHealth app*[Title/Abstract])) OR (mHealth application*[Title/Abstract])) OR (Wearable[Title/Abstract])) OR (Wearable Device*[Title/Abstract])) OR (Wearable Technolog*[Title/Abstract])) OR (Wearable Electronic Device*[Title/Abstract])) OR (Text*[Title/Abstract])) OR (Text-messag*[Title/Abstract])) OR (Short Message Service*[Title/Abstract])) OR (Email*[Title/Abstract])  Filters: from 2015/1/1 - 2024/9/30 | 2320793 |
| #9 | #7 OR #8  Filters: from 2015/1/1 - 2024/9/30 | 2330236 |
| #10 | #3 AND #6 AND #9  Filters: from 2015/1/1 - 2024/9/30 | 1251 |
| #11 | Randomized Controlled Trial[Publication Type]  Filters: from 2015/1/1 - 2024/9/30 | 229008 |
| #12 | controlled clinical trial[Publication Type]  Filters: from 2015/1/1 - 2024/9/30 | 235156 |
| #13 | randomized[Title/Abstract]  Filters: from 2015/1/1 - 2024/9/30 | 394848 |
| #14 | placebo[Title/Abstract]  Filters: from 2015/1/1 - 2024/9/30 | 97468 |
| #15 | clinical trials as topic [mesh:noexp]  Filters: from 2015/1/1 - 2024/9/30 | 31064 |
| #16 | randomly[Title/Abstract]  Filters: from 2015/1/1 - 2024/9/30 | 218773 |
| #17 | trial[Title]  Filters: from 2015/1/1 - 2024/9/30 | 186879 |
| #18 | #11 OR #12 OR #13 OR #14 OR #15 OR #16 OR #17  Filters: from 2015/1/1 - 2024/9/30 | 700416 |
| #19 | animals [mh] NOT humans [mh]  Filters: from 2015/1/1 - 2024/9/30 | 1134602 |
| #20 | #18 NOT #19  Filters: from 2015/1/1 - 2024/9/30 | 648604 |
| #21 | #10 AND #20  Filters: from 2015/1/1 - 2024/9/30 | 314 |

| **Ovid Medline** | | |
| --- | --- | --- |
| **Search** | **Query** | **Items found**  **20241001** |
| #1  MeSH | exp pulmonary disease, chronic obstructive/ or exp lung diseases, obstructive/ or exp pulmonary emphysema/ or exp Bronchitis, Chronic/  limit 1 to yr="2015 -Current" | 67707 |
| #2  Title/  Abstract | (Pulmonary Disease, Chronic Obstructive or Lung Diseases, Obstructive or COPD or Chronic Obstructive Pulmonary Disease or COAD or Chronic Obstructive Airway Disease or Chronic Obstructive Lung Disease or Airflow Obstruction*, Chronic or Chronic Airflow Obstruction* or Obstructive Lung Disease* or Obstructive Pulmonary Disease* or Pulmonary Disease*, Obstructive or Chronic Bronchitis or Pulmonary Emphysema).ti,ab.  limit 1 to yr="2015 -Current" | 49698 |
| #3 | 1 or 2 | 89849 |
| #4  MeSH | exp self-management/ or exp self care/ or exp self-control/  limit 1 to yr="2015 -Current" | 34291 |
| #5  All fields | (Self manag* or Self management or (Self care or Selfcare) or Self-control or Self-monitor*).af.  limit 1 to yr="2015 -Current" | 104825 |
| #6 | 4 or 5 | 112198 |
| #7  MeSH | exp Telemedicine/ or exp Mobile Applications/ or exp Wearable Electronic Devices/ or exp Electronic Mail/ or exp Telephone/ or exp Cell Phone/ or exp Text Messaging/ or exp Computers/ or exp Computers, Handheld/ or exp Smartphone/  limit 1 to yr="2015 -Current" | 78915 |
| #8  Title/Abstract | (Tele or Mobile or Mobile Health or (mHealth or m-Health) or (Telehealth or Tele-health) or (eHealth or e-Health) or Digital Health or Telecoaching or (Telemedicine or Tele-medicine) or Physiologic* Monitoring or (Monitoring, Patient or Patient Monitoring) or (telemonitor* or tele-monitor*) or home monitor* or distant monitor* or remote monitor* or telehomecare or telecare or (tele-manage* or telemanage*) or internet or digital or online or Web or technolog* or electronic* or (web-based or webbased) or internet-mediated or Phone* or Telephone* or Cell phone* or Mobile phone* or Smartphone* or iPhone* or iPad* or Computer* or Handheld Computer* or Personal Digital Assistant* or PDA Computer* or Pocket PC* or Tablet Computer* or Tablet* or (App or Apps) or Application* or Mobile app* or Mobile application* or Mobile phone app* or Mobile phone application* or Smartphone app* or Smartphone application* or Mobile health app* or Mobile health application* or mHealth app* or mHealth application* or Wearable or Wearable Device* or Wearable Technolog* or Wearable Electronic Device* or Text* or Text-messag* or Short Message Service* or Email*).ti,ab.  limit 1 to yr="2015 -Current" | 2282891 |
| #9 | 7 or 8 | 2293526 |
| #10 | 3 and 6 and 9 | 794 |
| #11 | randomized controlled trial.pt.  limit 1 to yr="2015 -Current" | 228068 |
| #12 | controlled clinical trial.pt.  limit 1 to yr="2015 -Current" | 6265 |
| #13 | randomized.ab.  limit 1 to yr="2015 -Current" | 355500 |
| #14 | placebo.ab.  limit 1 to yr="2015 -Current" | 9490 |
| #15 | clinical trials as topic.sh.  limit 1 to yr="2015 -Current" | 31121 |
| #16 | randomly.ab.  limit 1 to yr="2015 -Current" | 219207 |
| #17 | trial.ti.  limit 1 to yr="2015 -Current" | 187665 |
| #18 | 11 or 12 or 13 or 14 or 15 or 16 or 17 | 693009 |
| #19 | exp animals/ not humans.sh.  limit 1 to yr="2015 -Current" | 1136460 |
| #20 | 18 not 19 | 641242 |
| #21 | 10 and 20 | 213 |

| **Embase** | | |
| --- | --- | --- |
| **Search** | **Query** | **Items found**  **20241001** |
| #1  MeSH | ('chronic obstructive pulmonary disease'/exp OR 'obstructive lung diseases'/exp OR 'pulmonary emphysema'/exp OR 'chronic bronchitis'/exp OR 'chronic obstructive lung disease'/exp) AND [2015-2024]/py | 226328 |
| #2  Title/  Abstract | ('pulmonary disease, chronic obstructive':ti,ab OR 'lung diseases, obstructive':ti,ab OR copd:ti,ab OR 'chronic obstructive pulmonary disease':ti,ab OR coad:ti,ab OR 'chronic obstructive airway disease':ti,ab OR 'chronic obstructive lung disease':ti,ab OR 'airflow obstruction*, chronic':ti,ab OR 'chronic airflow obstruction*':ti,ab OR 'obstructive lung disease*':ti,ab OR 'obstructive pulmonary disease*':ti,ab OR 'pulmonary disease*, obstructive':ti,ab OR 'chronic bronchitis':ti,ab OR 'pulmonary emphysema':ti,ab) AND [2015-2024]/py | 90032 |
| #3 | #1 OR #2 | 237460 |
| #4  MeSH | ('self-management'/exp OR 'self care'/exp OR 'self-control'/exp) AND [2015-2024]/py | 70075 |
| #5  All fields | ('self manag*' OR 'self management' OR 'self care' OR 'selfcare' OR 'self-control' OR 'self-monitor*') AND [2015-2024]/py | 77711 |
| #6 | #4 OR #5 | 94665 |
| #7  MeSH | ('telemedicine'/exp OR 'mobile applications'/exp OR 'wearable electronic devices'/exp OR 'electronic mail'/exp OR 'telephone'/exp OR 'cell phone'/exp OR 'text messaging'/exp OR 'computers'/exp OR 'computers, handheld'/exp OR 'smartphone'/exp) AND [2015-2024]/py | 202960 |
| #8  [Title/Abstract] | (tele:ti,ab OR mobile:ti,ab OR 'mobile health':ti,ab OR mhealth:ti,ab OR 'm-health':ti,ab OR telehealth:ti,ab OR 'tele-health':ti,ab OR ehealth:ti,ab OR 'e-health':ti,ab OR 'digital health':ti,ab OR telecoaching:ti,ab OR telemedicine:ti,ab OR 'tele-medicine':ti,ab OR 'physiologic* monitoring':ti,ab OR 'monitoring, patient':ti,ab OR 'patient monitoring':ti,ab OR telemonitor*:ti,ab OR 'tele-monitor*':ti,ab OR 'home monitor*':ti,ab OR 'distant monitor*':ti,ab OR 'remote monitor*':ti,ab OR telehomecare:ti,ab OR telecare:ti,ab OR 'tele-manage*':ti,ab OR telemanage*:ti,ab OR internet:ti,ab OR digital:ti,ab OR online:ti,ab OR web:ti,ab OR technolog*:ti,ab OR electronic*:ti,ab OR 'web-based':ti,ab OR webbased:ti,ab OR 'internet-mediated':ti,ab OR phone*:ti,ab OR telephone*:ti,ab OR 'cell phone*':ti,ab OR 'mobile phone*':ti,ab OR smartphone*:ti,ab OR iphone*:ti,ab OR ipad*:ti,ab OR computer*:ti,ab OR 'handheld computer*':ti,ab OR 'personal digital assistant*':ti,ab OR 'pda computer*':ti,ab OR 'pocket pc*':ti,ab OR 'tablet computer*':ti,ab OR tablet*:ti,ab OR app:ti,ab OR apps:ti,ab OR application*:ti,ab OR 'mobile app*':ti,ab OR 'mobile application*':ti,ab OR 'mobile phone app*':ti,ab OR 'mobile phone application*':ti,ab OR 'smartphone app*':ti,ab OR 'smartphone application*':ti,ab OR 'mobile health app*':ti,ab OR 'mobile health application*':ti,ab OR 'mhealth app*':ti,ab OR 'mhealth application*':ti,ab OR wearable:ti,ab OR 'wearable device*':ti,ab OR 'wearable technolog*':ti,ab OR 'wearable electronic device*':ti,ab OR text*:ti,ab OR 'text-messag*':ti,ab OR 'short message service*':ti,ab OR email*:ti,ab) AND [2015-2024]/py | 2740707 |
| #9 | #7 OR #8 | 2781028 |
| #10 | #3 AND #6 AND #9 | 1606 |
| #11 | 'randomized controlled trial'/de AND [2015-2024]/py | 472332 |
| #12 | ('crossover procedure':de OR 'double-blind procedure':de OR 'randomized controlled trial':de OR 'single-blind procedure':de OR random*:de,ab,ti OR factorial*:de,ab,ti OR crossover*:de,ab,ti OR ((cross NEXT/1 over*):de,ab,ti) OR placebo*:de,ab,ti OR ((doubl* NEAR/1 blind*):de,ab,ti) OR ((singl* NEAR/1 blind*):de,ab,ti) OR assign*:de,ab,ti OR allocat*:de,ab,ti OR volunteer*:de,ab,ti) AND [2015-2024]/py | 1679219 |
| #13 | ('clinical trial'/de OR 'randomized controlled trial'/de OR 'randomization'/de OR 'single blind procedure'/de OR 'double blind procedure'/de OR 'crossover procedure'/de OR 'placebo'/de OR 'prospective study'/de OR ('randomi?ed controlled' NEXT/1 trial*) OR rct OR 'randomly allocated' OR 'allocated randomly' OR 'random allocation' OR (allocated NEAR/2 random) OR (single NEXT/1 blind*) OR (double NEXT/1 blind*) OR ((treble OR triple) NEAR/1 blind*) OR placebo*) AND [2015-2024]/py | 1578031 |
| #14 | #11 OR #12 OR #13 | 2350031 |
| #15 | #10 AND #14 | 578 |

| **CINAHL** | | |
| --- | --- | --- |
| **Search** | **Query** | **Items found**  **20241001** |
| #1  MeSH | (MH "Pulmonary Disease, Chronic Obstructive+") OR (MH "Lung Diseases, Obstructive+") OR (MH "Emphysema+") OR (MH "Bronchitis, Chronic")  **Setting limits**- Publication date: 20150101-20240931 | 24558 |
| #2  Title/  Abstract | TI ("Pulmonary Disease, Chronic Obstructive" OR "Lung Diseases, Obstructive" OR "COPD" OR "Chronic Obstructive Pulmonary Disease" OR "COAD" OR "Chronic Obstructive Airway Disease" OR "Chronic Obstructive Lung Disease" OR "Airflow Obstruction*, Chronic" OR "Chronic Airflow Obstruction*" OR "Obstructive Lung Disease*" OR "Obstructive Pulmonary Disease*" OR "Pulmonary Disease*, Obstructive" OR "Chronic Bronchitis" OR "Pulmonary Emphysema") OR AB ("Pulmonary Disease, Chronic Obstructive" OR "Lung Diseases, Obstructive" OR "COPD" OR "Chronic Obstructive Pulmonary Disease" OR "COAD" OR "Chronic Obstructive Airway Disease" OR "Chronic Obstructive Lung Disease" OR "Airflow Obstruction*, Chronic" OR "Chronic Airflow Obstruction*" OR "Obstructive Lung Disease*" OR "Obstructive Pulmonary Disease*" OR "Pulmonary Disease*, Obstructive" OR "Chronic Bronchitis" OR "Pulmonary Emphysema")  **Setting limits**- Publication date: 20150101-20240931 | 15772 |
| #3 | S1 OR S2 | 31107 |
| #4  MeSH | (MH "Self-Management") OR (MH "Self Care+") OR (MH "Self-Control (Iowa NOC)+")  **Setting limits**- Publication date: 20150101-20240931 | 29757 |
| #5  All fields | TX ("self manag*" OR "self-management" OR "self care" OR "self-control" OR "self-monitor*") OR AB ("self manag*" OR "self-management" OR "self care" OR "self-control" OR "self-monitor*")  **Setting limits**- Publication date: 20150101-20240931 | 50244 |
| #6 | S4 OR S5 | 53336 |
| #7  MeSH | (MH "Telemedicine+") OR **(MH "Telehealth")** OR **(MH "Telenursing")** OR (MH "Mobile Applications") OR (MH "Email") OR (MH "Telephone+") OR (MH "Cellular Phone+") OR (MH "Text Messaging+") OR (MH "Computers, Hand-Held+") OR (MH "Computers and Computerization+") OR (MH "Smartphone")  **Setting limits**- Publication date: 20150101-20240931 | 426135 |
| #8  [Title/Abstract] | TI (tele OR mobile OR "mobile health" OR mhealth OR "m-health" OR telehealth OR "tele-health" OR ehealth OR "e-health" OR "digital health" OR telecoaching OR telemedicine OR "tele-medicine" OR "physiologic* monitoring" OR "monitoring, patient" OR "patient monitoring" OR telemonitor* OR "tele-monitor*" OR "home monitor*" OR "distant monitor*" OR "remote monitor*" OR telehomecare OR telecare OR "tele-manage*" OR telemanage* OR internet OR digital OR online OR web OR technolog* OR electronic* OR "web-based" OR webbased OR "internet-mediated" OR phone* OR telephone* OR "cell phone*" OR "mobile phone*" OR smartphone* OR iphone* OR ipad* OR computer* OR "handheld computer*" OR "computer*, handheld" OR "personal digital assistant*" OR "pda computer*" OR "pocket pc*" OR "tablet computer*" OR tablet* OR app OR apps OR application* OR "mobile app*" OR "mobile application*" OR "mobile phone app*" OR "mobile phone application*" OR "smartphone app*" OR "smartphone application*" OR "mobile health app*" OR "mobile health application*" OR "mhealth app*" OR "mhealth application*" OR wearable OR "wearable device*" OR "wearable technolog*" OR "wearable electronic device*" OR text* OR "text-messag*" OR "short message service*" OR email*) OR AB (tele OR mobile OR "mobile health" OR mhealth OR "m-health" OR telehealth OR "tele-health" OR ehealth OR "e-health" OR "digital health" OR telecoaching OR telemedicine OR "tele-medicine" OR "physiologic* monitoring" OR "monitoring, patient" OR "patient monitoring" OR telemonitor* OR "tele-monitor*" OR "home monitor*" OR "distant monitor*" OR "remote monitor*" OR telehomecare OR telecare OR "tele-manage*" OR telemanage* OR internet OR digital OR online OR web OR technolog* OR electronic* OR "web-based" OR webbased OR "internet-mediated" OR phone* OR telephone* OR "cell phone*" OR "mobile phone*" OR smartphone* OR iphone* OR ipad* OR computer* OR "handheld computer*" OR "computer*, handheld" OR "personal digital assistant*" OR "pda computer*" OR "pocket pc*" OR "tablet computer*" OR tablet* OR app OR apps OR application* OR "mobile app*" OR "mobile application*" OR "mobile phone app*" OR "mobile phone application*" OR "smartphone app*" OR "smartphone application*" OR "mobile health app*" OR "mobile health application*" OR "mhealth app*" OR "mhealth application*" OR wearable OR "wearable device*" OR "wearable technolog*" OR "wearable electronic device*" OR text* OR "text-messag*" OR "short message service*" OR email*)  **Setting limits**- Publication date: 20150101-20240931 | 471984 |
| #9 | S7 OR S8 | 764113 |
| #10 | S3 AND S6 AND S9 | 513 |
| #11 | PT "Randomized Controlled Trial"  **Setting limits**- Publication date: 20150101-20240931 | 97245 |
| #12 | ( ( ( (MH "Random Assignment") or (MH "Random Sample+") or (MH "Crossover Design") or (MH "Clinical Trials+") or (MH "Comparative Studies") or (MH "Control (Research)+") or (MH "Control Group") or (MH "Factorial Design") or (MH "Quasi-Experimental Studies+") or (MH "Placebos") or (MH "Meta Analysis") or (MH "Sample Size") or (MH "Research, Nursing") or (MH "Research Question") or (MH "Research Methodology+") or (MH "Evaluation Research+") or (MH "Concurrent Prospective Studies") or (MH "Prospective Studies") or (MH "Nursing Practice, Research-Based") or (MH "Solomon Four-Group Design") or (MH "One-Shot Case Study") or (MH "Pretest-Posttest Design+") or (MH "Static Group Comparis on") or (MH "Study Design") or (MH "Clinical Research+") ) or ( clinical nursing research or random* or cross?over or placebo* or control* or factorial or sham* or meta?analy* or systematic review* or blind* or mask* or trial* ) ) )  **Setting limits**- Publication date: 20150101-20240931 | 1854788 |
| #13 | S11 OR S12 | 1854788 |
| #14 | S10 AND S13 | 426 |

| Web of Science | | |
| --- | --- | --- |
| **Search** | **Query** | **Items found**  **20241001** |
| #1 | TS=("chronic obstructive pulmonary disease" OR "pulmonary disease, chronic obstructive" OR "obstructive lung diseases" OR "lung diseases, obstructive" OR "chronic airflow obstruction*" OR "airflow obstruction*, chronic" OR "obstructive pulmonary disease*" OR "pulmonary disease*, obstructive" OR "pulmonary emphysema" OR "chronic bronchitis" OR "chronic obstructive lung disease" OR "chronic obstructive airway disease" OR "obstructive lung disease*" OR "COPD" OR "COAD")  *Timespan: 2015-01-01 to 2024-09-30 (Publication Date)* | 66512 |
| #2 | TS=("self-management" OR "self manag*" OR "self care" OR "selfcare" OR "self-control" OR "self-monitor*")  *Timespan: 2015-01-01 to 2024-09-30 (Publication Date)* | 55354 |
| #3 | TS=(tele OR mobile OR "mobile health" OR mhealth OR "m-health" OR telehealth OR "tele-health" OR ehealth OR "e-health" OR "digital health" OR telecoaching OR telemedicine OR "tele-medicine" OR "physiologic* monitoring" OR "monitoring, patient" OR "patient monitoring" OR telemonitor* OR "tele-monitor*" OR "home monitor*" OR "distant monitor*" OR "remote monitor*" OR telehomecare OR telecare OR "tele-manage*" OR telemanage* OR internet OR digital OR online OR web OR technolog* OR electronic* OR "web-based" OR webbased OR "internet-mediated" OR phone* OR telephone* OR "cell phone*" OR "mobile phone*" OR smartphone* OR iphone* OR ipad* OR computer* OR "handheld computer*" OR "computer*, handheld" OR "personal digital assistant*" OR "pda computer*" OR "pocket pc*" OR "tablet computer*" OR tablet* OR app OR apps OR application* OR "mobile app*" OR "mobile application*" OR "mobile phone app*" OR "mobile phone application*" OR "smartphone app*" OR "smartphone application*" OR "mobile health app*" OR "mobile health application*" OR "mhealth app*" OR "mhealth application*" OR wearable OR "wearable device*" OR "wearable technolog*" OR "wearable electronic device*" OR text* OR "text-messag*" OR "short message service*" OR email* OR "electronic mail*")  *Timespan: 2015-01-01 to 2024-09-30 (Publication Date)* | 4974113 |
| #4 | #1 AND #2 AND #3 | 528 |
| #5 | TS=("randomized controlled trial")  *Timespan: 2015-01-01 to 2024-09-30 (Publication Date)* | 123446 |
| #6 | TS=(clinical trial* OR research design OR comparative stud* OR evaluation stud* OR controlled trial* OR follow-up stud* OR prospective stud* OR random* OR placebo* OR single blind* OR double blind*)  *Timespan: 2015-01-01 to 2024-09-30 (Publication Date)* | 3283197 |
| #7 | #5 OR #6 | 3283197 |
| #8 | #4 AND #7 | 312 |

| **Cochrane Library** | | |
| --- | --- | --- |
| **Search** | **Query** | **Items found**  **20241001** |
| #1  MeSH | [mh "pulmonary disease, chronic obstructive"] or [mh "lung diseases, obstructive"] or [mh "pulmonary emphysema"] or [mh "Bronchitis, Chronic"]  with Cochrane Library publication date from Jan 2015 to Sep 2024 | 11553 |
| #2  Title/  Abstract | (Pulmonary Disease, Chronic Obstructive):ti,ab or (Lung Diseases, Obstructive):ti,ab or (COPD):ti,ab or (Chronic Obstructive Pulmonary Disease):ti,ab or (COAD):ti,ab or (Chronic Obstructive Airway Disease):ti,ab or (Chronic Obstructive Lung Disease):ti,ab or (Airflow Obstruction*, Chronic):ti,ab or (Chronic Airflow Obstruction*):ti,ab or (Obstructive Lung Disease*):ti,ab or (Obstructive Pulmonary Disease*):ti,ab or (Pulmonary Disease*, Obstructive):ti,ab or (Chronic Bronchitis):ti,ab or (Pulmonary Emphysema):ti,ab  with Cochrane Library publication date from Jan 2015 to Sep 2024 | 16935 |
| #3 | #1 or #2  with Cochrane Library publication date from Jan 2015 to Sep 2024 | 22968 |
| #4  MeSH | [mh "self-management"] or [mh "self care"] or [mh "self-control"]  with Cochrane Library publication date from Jan 2015 to Sep 2024 | 6025 |
| #5  All fields | Self manag* or Self management or Self care or Selfcare or Self-control or Self-monitor*  with Cochrane Library publication date from Jan 2015 to Sep 2024 | 61292 |
| #6 | #4 or #5  with Cochrane Library publication date from Jan 2015 to Sep 2024 | 61792 |
| #7  MeSH | [mh "Telemedicine"] or [mh "Mobile Applications"] or [mh "Wearable Electronic Devices"] or [mh "Electronic Mail"] or [mh "Telephone"] or [mh "Cell Phone"] or [mh "Text Messaging"] or [mh "Computers"] or [mh "Computers, Handheld"] or [mh "Smartphone"]  with Cochrane Library publication date from Jan 2015 to Sep 2024 | 10388 |
| #8  Title/Abstract | (Tele):ti,ab or (Mobile):ti,ab or (Mobile Health):ti,ab or (mHealth or m-Health):ti,ab or (Telehealth or Tele-health):ti,ab or (eHealth or e-Health):ti,ab or (Digital Health):ti,ab or (Telecoaching):ti,ab or (Telemedicine or Tele-medicine):ti,ab or (Physiologic* Monitoring):ti,ab or (Monitoring, Patient or Patient Monitoring):ti,ab or (telemonitor* or tele-monitor*):ti,ab or (home monitor*):ti,ab or (distant monitor*):ti,ab or (remote monitor*):ti,ab or (telehomecare):ti,ab or (telecare):ti,ab or (tele-manage* or telemanage*):ti,ab or (internet):ti,ab or (digital):ti,ab or (online):ti,ab or (Web):ti,ab or (technolog*):ti,ab or (electronic*):ti,ab or (web-based or webbased):ti,ab or (internet-mediated):ti,ab or (Phone*):ti,ab or (Telephone*):ti,ab or (Cell phone*):ti,ab or (Mobile phone*):ti,ab or (Smartphone*):ti,ab or (iPhone*):ti,ab or (iPad*):ti,ab or (Computer*):ti,ab or (Handheld Computer*):ti,ab or (Personal Digital Assistant*):ti,ab or (PDA Computer*):ti,ab or (Pocket PC*):ti,ab or (Tablet Computer*):ti,ab or (Tablet*):ti,ab or (App or Apps):ti,ab or (Application*):ti,ab or (Mobile app*):ti,ab or (Mobile application*):ti,ab or (Mobile phone app*):ti,ab or (Mobile phone application*):ti,ab or (Smartphone app*):ti,ab or (Smartphone application*):ti,ab or (Mobile health app*):ti,ab or (Mobile health application*):ti,ab or (mHealth app*):ti,ab or (mHealth application*):ti,ab or (Wearable):ti,ab or (Wearable Device*):ti,ab or (Wearable Technolog*):ti,ab or (Wearable Electronic Device*):ti,ab or (Text*):ti,ab or (Text-messag*):ti,ab or (Short Message Service*):ti,ab or (Email*):ti,ab  with Cochrane Library publication date from Jan 2015 to Sep 2024 | 270161 |
| #9 | #7 or #8  with Cochrane Library publication date from Jan 2015 to Sep 2024 | 270974 |
| #10 | #3 and #6 and #9  with Cochrane Library publication date from Jan 2015 to Sep 2024 | 895 |
| #11 | (Randomized Controlled Trial):pt  with Cochrane Library publication date from Jan 2015 to Sep 2024 | 897663 |
| #12 | #10 and #11  with Cochrane Library publication date from Jan 2015 to Sep 2024 | 691 |

| **Scopus** | | |
| --- | --- | --- |
| **Search** | **Query** | **Items found**  **20241001** |
| #1 | TITLE-ABS("chronic obstructive pulmonary disease" OR "pulmonary disease, chronic obstructive" OR "obstructive lung diseases" OR "lung diseases, obstructive" OR "chronic airflow obstruction*" OR "airflow obstruction*, chronic" OR "obstructive pulmonary disease*" OR "pulmonary disease*, obstructive" OR "pulmonary emphysema" OR "chronic bronchitis" OR "chronic obstructive lung disease" OR "chronic obstructive airway disease" OR "obstructive lung disease*" OR "COPD" OR "COAD")  Filter Published from 2015 to Present | 57461 |
| #2 | ALL("self-management" OR "self manag*" OR "self care" OR "selfcare" OR "self-control" OR "self-monitor*")  Filter Published from 2015 to 2024 | 324555 |
| #3 | TITLE-ABS(tele OR mobile OR "mobile health" OR mhealth OR "m-health" OR telehealth OR "tele-health" OR ehealth OR "e-health" OR "digital health" OR telecoaching OR telemedicine OR "tele-medicine" OR "physiologic* monitoring" OR "monitoring, patient" OR "patient monitoring" OR telemonitor* OR "tele-monitor*" OR "home monitor*" OR "distant monitor*" OR "remote monitor*" OR telehomecare OR telecare OR "tele-manage*" OR telemanage* OR internet OR digital OR online OR web OR technolog* OR electronic* OR "web-based" OR webbased OR "internet-mediated" OR phone* OR telephone* OR "cell phone*" OR "mobile phone*" OR smartphone* OR iphone* OR ipad* OR computer* OR "handheld computer*" OR "computer*, handheld" OR "personal digital assistant*" OR "pda computer*" OR "pocket pc*" OR "tablet computer*" OR tablet* OR app OR apps OR application* OR "mobile app*" OR "mobile application*" OR "mobile phone app*" OR "mobile phone application*" OR "smartphone app*" OR "smartphone application*" OR "mobile health app*" OR "mobile health application*" OR "mhealth app*" OR "mhealth application*" OR wearable OR "wearable device*" OR "wearable technolog*" OR "wearable electronic device*" OR text* OR "text-messag*" OR "short message service*" OR email* OR "electronic mail*")  Filter Published from 2015 to 2024 | 8907893 |
| #4 | #1 AND #2 AND #3 | 1306 |
| #5 | TITLE-ABS-KEY ("randomized controlled trial")  Filter Published from 2015 to 2024 | 574183 |
| #6 | TITLE-ABS-KEY((clinic* w/1 trial*) OR (randomi* w/1 control*) OR (randomi* w/2 trial*) OR (random* w/1 assign*) OR (random* w/1 allocat*) OR (control* w/1 clinic*) OR (control* w/1 trial) OR placebo* OR (Quantitat* w/1 Stud*) OR (control* w/1 stud*) OR (randomi* w/1 stud*) OR (singl* w/1 blind*) or (singl* w/1 mask*) OR (doubl* w/1 blind*) OR (doubl* w/1 mask*) OR (tripl* w/1 blind*) OR (tripl* w/1 mask*) OR (trebl* w/1 blind*) OR (trebl* w/1 mask*)) AND NOT (SRCTYPE(b) OR SRCTYPE(k) OR SRCTYPE(p) OR SRCTYPE(r) OR SRCTYPE(d) OR DOCTYPE(ab) OR DOCTYPE(bk) OR DOCTYPE(ch) OR DOCTYPE(bz) OR DOCTYPE(cr) OR DOCTYPE(ed) OR DOCTYPE(er) OR DOCTYPE(le) OR DOCTYPE(no) OR DOCTYPE(pr) OR DOCTYPE(rp) OR DOCTYPE(re) OR DOCTYPE(sh))  Filter Published from 2015 to 2024 | 9789578 |
| #7 | ( INDEXTERMS ( "clinical trials" OR "clinical trials as a topic" OR "randomized controlled trial" OR "Randomized Controlled Trials as Topic" OR "controlled clinical trial" OR "Controlled Clinical Trials" OR "random allocation" OR "Double-Blind Method" OR "Single-Blind Method" OR "Cross-Over Studies" OR "Placebos" OR "multicenter study" OR "double blind procedure" OR "single blind procedure" OR "crossover procedure" OR "clinical trial" OR "controlled study" OR "randomization" OR "placebo" ) ) OR ( TITLE-ABS-KEY ( ( "clinical trials" OR "clinical trials as a topic" OR "randomized controlled trial" OR "Randomized Controlled Trials as Topic" OR "controlled clinical trial" OR "Controlled Clinical Trials as Topic" OR "random allocation" OR "randomly allocated" OR "allocated randomly" OR "Double-Blind Method" OR "Single-Blind Method" OR "Cross-Over Studies" OR "Placebos" OR "cross-over trial" OR "single blind" OR "double blind" OR "factorial design" OR "factorial trial" ) ) ) OR ( TITLE-ABS ( clinical trial* OR trial* OR rct* OR random* OR blind* ) )  Filter Published from 2015 to 2024 | 4575191 |
| #8 | #5 OR #6 OR #7 | 10322202 |
| #9 | #4 AND #8 | 602 |

This document is a supplementary appendix to a full article published in the Journal of Medical Internet Research (J Med Internet Res). For complete copyright and citation details, please refer to the main manuscript.
