# Supplementary material for: Effectiveness of Mobile Health–Based Self-Management Programs on Health-Related Outcomes in Patients With Chronic Obstructive Pulmonary Disease: Systematic Review and Meta-Analysis
Source: JMIR Mhealth Uhealth. 2025 Dec 29;13:e74967. doi: 10.2196/74967 (PMC12747663; doi:10.2196/74967)
Supplement: Multimedia Appendix 2 [file mhealth-v13-e74967-s002.docx]

**Table S2**

| Number | Author (year), country | Setting | Comparator | | Age, year, mean (SD^a^) | | | Sex, male, n (%) | | | |  |
| --- | --- | --- | --- | --- | --- | --- | --- | --- | --- | --- | --- | --- |
|  |  |  |  |  | IG^b^ | | CG^c^ | IG^b^ | | CG^c^ | |  |
| ***Web-based and computer-based programs*** | | | |  | |  | | |  | |  | |
| 1 | Arbillaga-Etxarri [38] (2018), Spain | Community-based exercise training program | Usual care | | 68.0 (9.0) | | 69.0 (8.0) | 114 (86.0) | | 130 (88.0) | |  |
| 2 | Benzo [41] (2021), USA | Home-based program with health coaching | Regular PR^d^ program | | 69.4 (8.1) | | 68.5 (9.1) | 34 (47) | | 37 (50) | |  |
| 3 | Benzo [42] (2022), USA | Home-based program with health coaching | Regular PR^d^ program | | 69.3 (9.5) | | 68.7 (9.5) | 87 (46.3) | | 76 (40.6 | |  |
| 4 | Boer [43] (2019), Netherlands | Home-based telemonitoring | Usual care without PR^d^. | | 69.3 (8.8) | | 65.9 (8.9) | 25 (58.1) | | 29 (65.9) | |  |
| 5 | Bourne [44] (2017), UK | Community-based pulmonary rehabilitation | Face-to-face PR^d^ | | 69.1 (7.9) | | 71.4 (8.6) | 41 (64.1) | | 18 (69.2) | |  |
| 6 | Chan [49] (2016), Taiwan | Hospital-based breathing technique program | Face-to-face PR^d^ | | 73.4 (11.0) | | 69.4 (12.0) | 33 (91.7) | | 26 (74.3) | |  |
| 7 | Farmer [50] (2017), UK | Home-based telemonitoring | Usual care. | | 69.8 (9.1) | | 69.8 (10.6) | 68 (61.8) | | 34 (60.7) | |  |
| 8 | Ho [55] (2016), Taiwan | Home-based telemonitoring | Usual care without PR^d^ | | 81.4 (7.8) | | 79.9 (9.6) | 43 (81.1) | | 38 (71.7) | |  |
| 9 | Kessler [57] (2018), France | Home-based disease management | Usual care without PR^d^ | | 67.3 (8.9) | | 66.6 (9.6) | 109 (69.4) | | 113 (69.8) | |  |
| 10 | Moy [58] (2016), USA | Home-based exercise and self-monitoring | Pedometer only. | | 67.0 (8.6) | | 66.4 (9.2) | 146 (94.8) | | 77 (92.0) | |  |
| 11 | Rixon [70] (2017), UK | Home-based telehealth services | Usual care without PR^d^ | | 71.5 (9.3) | | 72.2 (9.2) | 188 (56.0) | | 138 (57.0) | |  |
| 12 | Robinson [71] (2021), USA | Home-based self-management | Written material and a pedometer | | 69.2 (7.2) | | 70.4 (7.3) | 70 (93) | | 72 (92) | |  |
| 13 | Saleh [72] (2023), Norway | Home-based telemedicine | Standard Practice without PR^d^ | | 69.0 (8.8) | | 68.1 (8.6) | 15 (26.3) | | 26 (45.6) | |  |
| 14 | Stamenova [73] (2020), Canada | Home-based remote monitoring | Standard care without PR^d^ | | 72.0 (9.5) | | 72.8 (9.2) | 23 (56.0) | | 21 (52.0) | |  |
| 15 | Tsai (2017) [64], Australia | Home-based telerehabilitation | Usual care without PR^d^. | | 73.0 (8.0) | | 75.0 (9.0) | 12 (62.2) | | 6 (35.3) | |  |
| 16 | Vasilopoulou [55] (2017), Greece | Home-based telerehabilitation program | Usual Care without PR^d^ | | 66.9 (9.6) | | 64.0 (8.0) | 44 (93.6) | | 37 (74.0) | |  |
| 17 | Vianello [52] (2016), Italy | Home-based telemonitoring | Usual care without PR^d^ | | 76.0 (6.5) | | 76.5 (6.2) | 164 (71.3) | | 76 (73.1) | |  |
| 18 | Walker [45] (2018), UK | Home-based telemonitoring | Usual care without PR^d^ | | 71.0 (9.8) | | 71.0 (10.7) | 101 (65.6) | | 105 (66.5) | |  |
| 19 | Wan [68] (2017), USA | Home-based physical activity monitoring | Written materials and a pedometer. | | 68.4 (8.7) | | 68.8 (7.9) | 56 (98.3) | | 51 (98.1) | |  |
| 20 | Wan [69] (2020), USA | Home-based physical activity monitoring | Written materials and a pedometer. | | 68.4 (8.7) | | 68.7 (7.9) | 56 (98.3) | | 51 (98.1) | |  |
| 21 | Wang [59] (2017), China | Home-based coaching program | Routine care without PR^d^. | | 69.3 (7.8) | | 71.9 (8.1) | 21 (38.0) | | 36 (55.0) | |  |
| 22 | Zanaboni [51] (2023), Norway, Denmark, and Australia | Home-based supervised in-person training session | Standard care | | 64.9 (7.1) | | 63.5 (8.0) | 23 (57.5) | | 23 (57.5) | |  |
| 23 | Holland (2017), Australia | Home-based pulmonary rehabilitation | Center-based PR^d^. | | 69.0 (13.0) | | 69.0 (10.0) | 48 (60.0) | | 51 (59.3) | |  |
| 24 | Jolly [46] (2018), UK | Home-based telemonitoring | Usual care without PR^d^. | | 70.7 (8.8) | | 70.2 (7.8) | 183 (63.3) | | 183 (63.5) | |  |
| 25 | Varas [39] (2018), Spain | Community-based exercise training program | Conventional program and pedometer. | | 69.5 (7.4) | | 64.8 (9.1) | 18 (85.7) | | 13 (68.4) | |  |
| 26 | Wootton [66] (2018), Australia | Home-based walking training program | Usual care without PR^d^ | | 70.0 (7.0) | | 69.0 (9.0) | 25 (51.0) | | 30 (65.0) | |  |
| 27 | Bi [60] (2021), China | Home-based instant communication platform education | Usual Care | | 69.4 (7.4) | | 68.9 (5.3) | 65 (65.0) | | 68 (68.0) | |  |
| 28 | Cerdán-De-las-heras [53] (2022), Denmark | Home-based Tele-rehabilitation | Standard rehabilitation | | 67.4 (10.2) | | 72.5 (7.4) | 16 (51.6) | | 15 (48.4) | |  |
| 29 | Crooks [47] (2020), UK | Home-based self-management | Usual care without PR^d^ | | 65.9 (7.3) | | 66.4 (7.0) | 11 (37.9) | | 20 (64.5) | |  |
| 30 | Jiang [61] (2020), China | Home-based pulmonary rehabilitation | Face-to-face PR^d^ | | 70.9 (6.4) | | 71.8 (7.6) | 44 (83.0) | | 43 (81.0) | |  |
| 31 | Jimenez-Reguera [40] (2020), Spain | Home-based telemonitoring | Conventional PR^d^ | | 68.1 (6.6) | | 68.1 (7.0) | 9 (52.9) | | 13 (68.4) | |  |
| 32 | Loeckx [56] (2023), Belgium | Hospital-based pulmonary rehabilitation program | Usual Care | | 62.0 (7.0) | | 66.0 (8.0) | 22 (61%) | | 20 (54%) | |  |
| 33 | North [48] (2020), UK | Home-based self-management | Usual care without PR^d^ | | 65.1 (6.3) | | 68.1 (7.4) | 13 (65.0) | | 11 (52.0) | |  |
| 34 | Park [63] (2020), South Korea | Home-based education, exercise, self-monitoring, and social support | Conventional PR^d^ and calls. | | 70.5 (9.4) | | 65.1 (11.1) | 19 (86.4) | | 14 (70.0) | |  |
| 35 | Spielmanns [54]ti (2023), Germany and Switzerland | Home-based physical exercise training | Standard care | | 66.1 (6.8) | | 62.7 (8.2) | 17 (51.5) | | 17 (50.0) | |  |
| 36 | Wang [62] (2021), China | Home-based self-management | Routine care without PR^d^ | | 63.2 (7.5) | | 64.4 (7.0) | 26 (66.7) | | 29 (74.4) | |  |

^a^SD: standard deviation.

^b^IG: intervention group.

^c^CG: control group.

^d^PR: pulmonary rehabilitation.

This document is a supplementary appendix to a full article published in the Journal of Medical Internet Research (J Med Internet Res). For complete copyright and citation details, please refer to the main manuscript

**Figure S1**


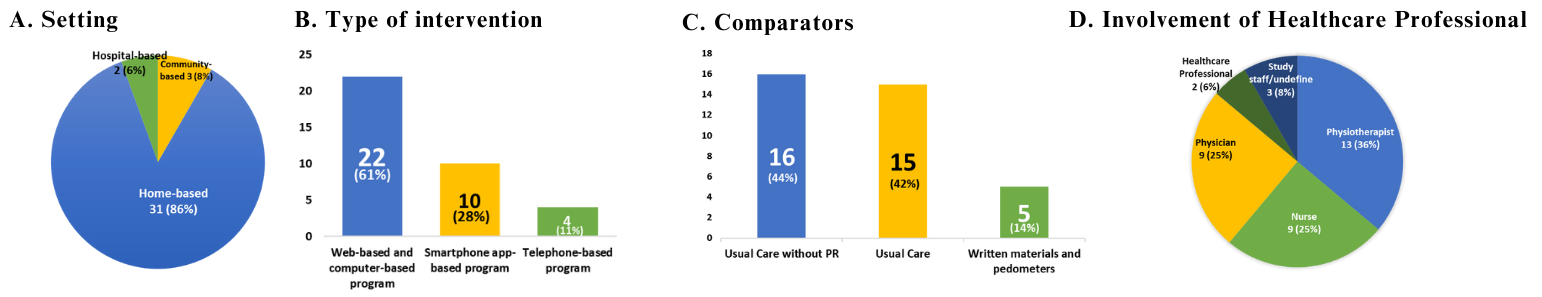


REFERENCES

38. Arbillaga-Etxarri A, Gimeno-Santos E, Barberan-Garcia A, et al. Long-term efficacy and effectiveness of a behavioural and community-based exercise intervention (urban training) to increase physical activity in patients with COPD: a randomised controlled trial. Eur Respir J. Oct 2018;52(4):1800063. [doi: 10.1183/13993003.00063-2018] [Medline: 30166322]

70. Benzo RP, Ridgeway J, Hoult JP, et al. Feasibility of a health coaching and home-based rehabilitation intervention with remote monitoring for COPD. Respir Care. Jun 2021;66(6):960-971. [doi: 10.4187/respcare.08580] [Medline: 33906954]

71. Benzo R, Hoult J, McEvoy C, et al. Promoting chronic obstructive pulmonary disease wellness through remote monitoring and health coaching: a clinical trial. Ann Am Thorac Soc. Nov 2022;19(11):1808-1817. [doi: 10.1513/AnnalsATS.202203-214OC] [Medline: 35914215]

41. Boer L, Bischoff E, van der Heijden M, et al. A smart mobile health tool versus a paper action plan to support self-management of chronic obstructive pulmonary disease exacerbations: randomized controlled trial. JMIR Mhealth Uhealth. Oct 9, 2019;7(10):e14408. [doi: 10.2196/14408] [Medline: 31599729]

42. Bourne S, DeVos R, North M, et al. Online versus face-to-face pulmonary rehabilitation for patients with chronic obstructive pulmonary disease: randomised controlled trial. BMJ Open. Jul 17, 2017;7(7):e014580. [doi: 10.1136/bmjopen-2016-014580] [Medline: 28716786]

57. Chan HY, Dai YT, Hou IC. Evaluation of a tablet-based instruction of breathing technique in patients with COPD. Int J Med Inform. Oct 2016;94(263-70):263-270. [doi: 10.1016/j.ijmedinf.2016.06.018] [Medline: 27573335]\

43. Farmer A, Williams V, Velardo C, et al. Self-management support using a digital health system compared with usual care for chronic obstructive pulmonary disease: randomized controlled trial. J Med Internet Res. May 3, 2017;19(5):e144. [doi: 10.2196/jmir.7116] [Medline: 28468749]

58. Ho TW, Huang CT, Chiu HC, et al. Effectiveness of telemonitoring in patients with chronic obstructive pulmonary disease in taiwan-a randomized controlled trial. Sci Rep. Mar 31, 2016;6(23797):23797. [doi: 10.1038/srep23797] [Medline: 27029815]

49. Kessler R, Casan-Clara P, Koehler D, et al. COMET: a multicomponent home-based disease-management programme versus routine care in severe COPD. Eur Respir J. Jan 2018;51(1):29326333. [doi: 10.1183/13993003.01612-2017] [Medline: 29326333]

67. Moy ML, Martinez CH, Kadri R, et al. Long-term effects of an internet-mediated pedometer-based walking program for chronic obstructive pulmonary disease: randomized controlled trial. J Med Internet Res. Aug 8, 2016;18(8):e215. [doi: 10.2196/jmir.5622] [Medline: 27502583]

44. Rixon L, Hirani SP, Cartwright M, et al. A RCT of telehealth for COPD patient’s quality of life: the whole system demonstrator evaluation. Clin Respir J. Jul 2017;11(4):459-469. [doi: 10.1111/crj.12359] [Medline: 26260325]

72. Robinson SA, Cooper JA Jr, Goldstein RL, et al. A randomised trial of a web-based physical activity self-management intervention in COPD. ERJ Open Res. Jul 2021;7(3):00158-2021. [doi: 10.1183/23120541.00158-2021] [Medline: 34476247]

50. Saleh S, Skeie S, Grundt H. Re-admission and quality of life among patients with chronic obstructive pulmonary disease after telemedicine video nursing consultation - a randomized study. Multidiscip Respir Med. Jan 17, 2023;18(1):918. [doi: 10.4081/mrm.2023.918] [Medline: 37753200]

73. Stamenova V, Liang K, Yang R, et al. Technology-enabled self-management of chronic obstructive pulmonary disease with or without asynchronous remote monitoring: randomized controlled trial. J Med Internet Res. Jul 30, 2020;22(7):e18598. [doi: 10.2196/18598] [Medline: 32729843]

55. Vasilopoulou M, Papaioannou AI, Kaltsakas G, et al. Home-based maintenance tele-rehabilitation reduces the risk for acute exacerbations of COPD, hospitalisations and emergency department visits. Eur Respir J. May 2017;49(5):1602129. [doi: 10.1183/13993003.02129-2016] [Medline: 28546268]

52. Vianello A, Fusello M, Gubian L, et al. Home telemonitoring for patients with acute exacerbation of chronic obstructive pulmonary disease: a randomized controlled trial. BMC Pulm Med. Nov 22, 2016;16(1):157. [doi: 10.1186/s12890-016-0321-2] [Medline: 27876029]

45. Walker PP, Pompilio PP, Zanaboni P, et al. Telemonitoring in Chronic Obstructive Pulmonary Disease (CHROMED). A Randomized Clinical Trial. Am J Respir Crit Care Med. Sep 1, 2018;198(5):620-628. [doi: 10.1164/rccm.201712-2404OC] [Medline: 29557669]

68. Wan ES, Kantorowski A, Homsy D, et al. Promoting physical activity in COPD: insights from a randomized trial of a web-based intervention and pedometer use. Respir Med. Sep 2017;130(102-10):102-110. [doi: 10.1016/j.rmed.2017.07.057] [Medline: 29206627]

69. Wan ES, Kantorowski A, Polak M, et al. Long-term effects of web-based pedometer-mediated intervention on COPD exacerbations. Respir Med. Feb 2020;162:105878. [doi: 10.1016/j.rmed.2020.105878] [Medline: 32056676]

59. Wang L, He L, Tao Y, et al. Evaluating a web-based coaching program using electronic health records for patients with chronic obstructive pulmonary disease in China: randomized controlled trial. J Med Internet Res. Jul 21, 2017;19(7):e264. [doi: 10.2196/jmir.6743] [Medline: 28733270]

51. Zanaboni P, Dinesen B, Hoaas H, et al. Long-term telerehabilitation or unsupervised training at home for patients with chronic obstructive pulmonary disease: a randomized controlled trial. Am J Respir Crit Care Med. Apr 1, 2023;207(7):865-875. [doi: 10.1164/rccm.202204-0643OC] [Medline: 36480957]

46. Jolly K, Sidhu MS, Hewitt CA, et al. Self management of patients with mild COPD in primary care: randomised controlled trial. BMJ. Jun 13, 2018;361:k2241. [doi: 10.1136/bmj.k2241] [Medline: 29899047]

39. Varas AB, Córdoba S, Rodríguez-Andonaegui I, Rueda MR, García-Juez S, Vilaró J. Effectiveness of a community-based exercise training programme to increase physical activity level in patients with chronic obstructive pulmonary disease: a randomized controlled trial. Physiother Res Int. Oct 2018;23(4):e1740. [doi: 10.1002/pri.1740] [Medline: 30168228]

66. Wootton SL, McKeough Z, Ng CLW, et al. Effect on health-related quality of life of ongoing feedback during a 12-month maintenance walking programme in patients with COPD: a randomized controlled trial. Respirology. Jan 2018;23(1):60-67. [doi: 10.1111/resp.13128] [Medline: 28758320]

60. Bi J, Yang W, Hao P, et al. WeChat as a platform for baduanjin intervention in patients with stable chronic obstructive pulmonary disease in China: retrospective randomized controlled trial. JMIR Mhealth Uhealth. Feb 2, 2021;9(2):e23548. [doi: 10.2196/23548] [Medline: 33528369]

53. Cerdán-de-las-Heras J, Balbino F, Løkke A, Catalán-Matamoros D, Hilberg O, Bendstrup E. Effect of a new tele-rehabilitation program versus standard rehabilitation in patients with chronic obstructive pulmonary disease. JCM. 2022;11(1):11. [doi: 10.3390/jcm11010011]

47. Crooks MG, Elkes J, Storrar W, et al. Evidence generation for the clinical impact of myCOPD in patients with mild, moderate and newly diagnosed COPD: a randomised controlled trial. ERJ Open Res. Oct 2020;6(4):1-10. [doi: 10.1183/23120541.00460-2020] [Medline: 33263052]

61. Jiang Y, Liu F, Guo J, et al. Evaluating an intervention program using WeChat for patients with chronic obstructive pulmonary disease: randomized controlled trial. J Med Internet Res. Apr 21, 2020;22(4):e17089. [doi: 10.2196/17089] [Medline: 32314971]

40. Jiménez-Reguera B, Maroto López E, Fitch S, et al. Development and preliminary evaluation of the effects of an mHealth web-based platform (HappyAir) on adherence to a maintenance program after pulmonary rehabilitation in patients with chronic obstructive pulmonary disease: randomized controlled trial. JMIR Mhealth Uhealth. Jul 31, 2020;8(7):e18465. [doi: 10.2196/18465] [Medline: 32513646]

56. Loeckx M, Rodrigues FM, Blondeel A, et al. Sustaining training effects through physical activity coaching (STEP): a randomized controlled trial. Int J Behav Nutr Phys Act. Oct 10, 2023;20(1):121. [doi: 10.1186/s12966-023-01519-w] [Medline: 37814266]

48. North M, Bourne S, Green B, et al. A randomised controlled feasibility trial of E-health application supported care vs usual care after exacerbation of COPD: the RESCUE trial. NPJ Digit Med. 2020;3(1):145. [doi: 10.1038/s41746-020-00347-7] [Medline: 33145441]

63. Park SK, Bang CH, Lee SH. Evaluating the effect of a smartphone app-based self-management program for people with COPD: a randomized controlled trial. Appl Nurs Res. Apr 2020;52:151231. [doi: 10.1016/j.apnr.2020.151231] [Medline: 31955942]

54. Spielmanns M, Gloeckl R, Jarosch I, et al. Using a smartphone application maintains physical activity following pulmonary rehabilitation in patients with COPD: a randomised controlled trial. Thorax. May 2023;78(5):442-450. [doi: 10.1136/thoraxjnl-2021-218338] [Medline: 35450945]

62. Wang L, Guo Y, Wang M, Zhao Y. A mobile health application to support self-management in patients with chronic obstructive pulmonary disease: a randomised controlled trial. Clin Rehabil. Jan 2021;35(1):90-101. [doi: 10.1177/0269215520946931]ss
