# Supplementary material for: Effectiveness of Mobile Health–Based Self-Management Programs on Health-Related Outcomes in Patients With Chronic Obstructive Pulmonary Disease: Systematic Review and Meta-Analysis
Source: JMIR Mhealth Uhealth. 2025 Dec 29;13:e74967. doi: 10.2196/74967 (PMC12747663; doi:10.2196/74967)
Supplement: Multimedia Appendix 3 [file mhealth-v13-e74967-s003.docx]

**Table S3.** Summary of economic outcomes.

| Study (year) | Outcomes | | |
| --- | --- | --- | --- |
|  | ^a^CUA | Cost | ^b^QALY |
| ***Web-based and computer-based program*** | | | |
| Farmer (2017). |  | + |  |
| Walker (2018). | + | + | + |

^a^CUA : Cost-Utility Analysis

^b^QALY : Quality-adjusted Life Years

**Table S4.** Summary of clinical outcomes.

| Study (year) | Outcomes | | | | | | | | | | | | | | | | | | | |
| --- | --- | --- | --- | --- | --- | --- | --- | --- | --- | --- | --- | --- | --- | --- | --- | --- | --- | --- | --- | --- |
|  | ^a^6MWT | ^b^BDI-2 | ^c^CAT | ^d^CCQ | Clinic Visit/Consultation | ^e^COTE | ^f^Dyspnea scale (MRC) | ^g^ER Visit | ^h^ESWT | Exacerbation | ^i^HADS | Hospital Admission | ^j^ISWT | Mortality/Death | Number of Exacerbation-free | ^k^PHQ-9 | ^l^STAI-6 | Time to First Admission | ^m^UCSD-SOBQ | |
| ***Web-based and computer-based program*** | | | | | | | | | | | | | | | | | | | |  |
| Arbillaga (2018). | + |  | + | + |  |  |  |  |  | + | + |  |  |  |  |  |  |  |  | |
| Benzo (2021). |  |  |  |  |  |  |  |  |  |  |  |  |  |  |  |  |  |  |  | |
| Benzo (2022). |  |  |  |  |  |  | + |  |  |  |  |  |  |  |  | + |  |  |  | |
| Boer (2019). |  |  |  | + |  |  |  |  |  | + |  |  |  |  |  |  |  |  |  | |
| Bourne (2017). | + |  | + |  |  |  | + |  |  |  | + |  |  |  |  |  |  |  |  | |
| Chan (2016). |  |  | + |  |  |  |  |  |  |  |  |  |  |  |  |  |  |  |  | |
| Farmer (2017). |  |  |  |  |  |  |  |  |  |  |  |  |  | + | + |  |  |  |  | |
| Ho  (2016). |  |  |  |  |  |  |  | + |  |  |  | + |  |  |  |  |  | + |  | |
| Kessler (2018). | + |  |  |  |  |  |  |  |  | + | + | + |  | + |  |  |  |  |  | |
| Moy (2016). |  |  |  |  |  |  |  |  |  |  |  |  |  |  |  |  |  |  |  | |
| Rixon (2017). |  |  |  |  |  |  |  |  |  |  |  |  |  |  |  |  | + |  |  | |
| Robinson (2021). | + | + |  |  |  |  | + |  |  |  |  |  |  |  |  |  |  |  |  | |
| Saleh (2023). |  |  | + |  |  |  |  |  |  |  | + | + |  |  |  |  |  |  |  | |
| Stamenova (2020). |  | + |  |  | + |  |  | + |  | + |  | + |  |  |  |  |  |  |  | |
| Tsai (2017). | + |  | + |  |  |  | + |  | + |  | + |  | + |  |  |  |  |  |  | |
| Vasilopoulou (2017) | + |  | + |  |  |  | + | + |  | + |  | + |  |  |  |  |  |  |  | |
| Vianello (2016). |  |  |  |  | + |  |  | + |  |  | + | + |  | + |  |  |  |  |  | |
| Walker (2018). |  |  | + |  |  |  |  |  |  | + |  | + |  |  |  | + |  | + |  | |
| Wan (2017). | + | + |  |  |  |  | + |  |  |  |  |  |  |  |  |  |  |  |  | |
| Wan (2020). | + |  |  |  |  |  |  |  |  |  |  |  |  |  |  |  |  |  |  | |
| Wang (2017). | + |  |  |  |  |  | + |  |  |  |  |  |  |  |  |  |  |  |  | |
| Zanaboni (2023). | + |  | + |  |  |  | + | + |  |  | + | + |  |  |  |  |  | + |  | |
| ***Telephone-based program*** | | | | | | | | | | | | | | | | | | | |  |
| Holland (2017). | + |  |  |  |  | + | + |  |  |  | + |  |  |  |  |  |  |  |  | |
| Jolly (2018). |  |  |  |  | + |  | + | + |  |  | + | + |  |  |  |  |  |  |  | |
| Varas (2018). | + |  |  |  |  |  | + |  | + | + |  |  | + |  |  |  |  |  |  | |
| Wootton (2018). | + |  |  |  |  |  |  |  | + |  | + |  | + |  |  |  |  |  |  | |
| ***Smartphone app-based program*** | | | | | | | | | | | | | | | | | | | |  |
| Bi (2021). |  |  | + |  |  |  |  |  |  |  |  |  |  |  |  |  |  |  |  | |
| Cerdán-De-las-heras (2022). | + |  |  |  |  |  |  |  |  |  |  |  |  |  |  |  |  |  |  | |
| Crooks (2020). |  |  | + |  |  |  |  |  |  | + |  |  |  |  |  |  |  |  |  | |
| Jiang (2020). |  |  | + |  |  |  | + |  |  |  |  |  |  |  |  |  |  |  |  | |
| Jimenez-Reguera (2020). | + |  | + |  |  |  |  |  |  |  |  |  |  |  |  |  |  |  |  | |
| Loeckx (2023) | + |  |  |  |  |  |  |  |  |  |  |  |  |  |  |  |  |  |  | |
| North (2020). |  |  | + |  |  |  | + |  |  | + | + |  |  |  |  |  |  |  |  | |
| Park (2020). | + |  |  |  | + |  |  | + |  |  |  | + |  |  |  |  |  |  | + | |
| Spielmanns (2023). |  |  | + |  |  |  |  |  |  | + | + |  |  |  |  |  |  |  |  | |
| Wang (2021). |  |  | + |  |  |  |  |  |  |  |  |  |  |  |  |  |  |  |  | |

^a^6MWT : 6-Minutes Walking Test

^b^BDI-2 : Beck Depression Inventory-II

^c^CAT : COPD Assessment Test

^d^CCQ : Clinical COPD Questionnaire (CCQ)

^e^COTE : COPD-spesific Comorbidity Test

^f^Dyspnea scale (MRC) : Medical Research Council

^g^ER : Emergency Room

^h^ESWT : Endurance Shuttle Walking Test

^i^HADS : Hospital Anxiety and Depression Scale

^j^ISWT : Incremental Shuttle Walk Test

^k^PHQ-9 : Patient Health Questionnaire scores each of the 9 DSM-IV criteria

^l^STAI-6 : Brief State Trait Anxiety Inventory

^m^UCSD-SOBQ : University of California, San Diego Shortness of Breath Questionnaire

**Table S5.** Summary of humanistic outcome instruments.

| Study (year) | Questionnaires | | | | | | | | | | | | | | | | | | |
| --- | --- | --- | --- | --- | --- | --- | --- | --- | --- | --- | --- | --- | --- | --- | --- | --- | --- | --- | --- |
|  | ^a^BCKQ | ^b^BMQ | ^c^BPAQ | ^d^CAP-FISIO | ^e^CRQ | ^f^EQoL-5D | ^g^Exa self-efficacy | ^h^Ex-Self-Efficacy | ^i^MARS | ^j^MLHRQ | Morisky Green | ^k^PIH | ^l^PRAISE | ^m^SCBI | ^n^SEMCD | ^o^SF-12 | ^p^SF-36 | ^q^SGRQ | ^r^Stanford SES |
| ***Web-based and computer-based program*** | | | | | | | | | | | | | | | | | | | |
| Arbillaga (2018). |  |  |  |  |  |  |  |  |  |  |  |  |  |  |  |  |  |  |  |
| Benzo (2022). |  |  |  |  | + |  |  |  |  |  |  |  |  |  |  |  |  |  |  |
| Benzo (2021). |  |  |  |  | + |  |  |  |  |  |  |  |  |  |  |  |  |  |  |
| Boer (2019). |  |  |  |  |  | + | + |  |  |  |  |  |  |  |  |  |  |  |  |
| Bourne (2017). |  |  |  |  |  |  |  |  |  |  |  |  |  |  |  |  |  | + |  |
| Chan (2016). |  |  |  |  |  |  |  |  |  |  |  |  |  |  |  |  |  |  |  |
| Farmer (2017). |  | + |  |  |  | + |  |  | + |  |  |  |  |  |  |  |  | + |  |
| Kessler (2018). |  |  |  |  |  |  |  |  |  |  |  |  |  |  |  |  |  | + |  |
| Moy (2016). |  |  |  |  |  |  |  |  |  |  |  |  |  |  |  |  |  | + |  |
| Rixon (2017). |  |  |  |  | + | + |  |  |  |  |  |  |  |  |  | + |  |  |  |
| Robinson (2021). | + |  |  |  |  |  |  |  |  |  |  |  |  |  |  |  |  | + |  |
| Saleh (2023). |  |  |  |  |  |  |  |  |  |  |  |  |  |  |  |  |  |  |  |
| Stamenova (2020). | + |  |  |  |  |  |  |  |  |  |  | + |  |  |  |  |  | + |  |
| Teylan (2019). |  |  |  |  |  |  |  |  |  |  |  |  |  |  |  |  |  |  |  |
| Tsai (2017). |  |  |  |  | + |  |  |  |  |  |  |  | + |  |  |  |  |  |  |
| Vasilopoulou (2017) |  |  |  |  |  |  |  |  |  |  |  |  |  |  |  |  |  | + |  |
| Vianello (2016). |  |  |  |  |  |  |  |  |  |  |  |  |  |  |  |  | + |  |  |
| Walker (2018). |  |  |  |  |  | + |  |  |  | + |  |  |  |  |  |  |  |  |  |
| Wan (2017). | + |  |  |  |  |  |  | + |  |  |  |  |  |  |  |  |  | + |  |
| Wan (2020). |  |  |  |  |  |  |  | + |  |  |  |  |  |  |  |  |  | + |  |
| Wang (2017). |  |  |  |  |  |  |  |  |  |  |  |  |  |  |  |  |  | + |  |
| Zanaboni (2023). |  |  |  |  |  | + |  |  |  |  |  |  |  |  |  |  |  |  |  |
| ***Telephone-based program*** | | | | | | | | | | | | | | | | | | | |
| Holland (2017). |  |  |  |  | + |  |  |  |  |  |  |  | + |  |  |  |  |  |  |
| Jolly (2018). |  |  |  |  |  | + |  |  |  |  |  |  |  |  |  |  |  | + | + |
| Varas (2018). |  |  | + |  |  |  |  |  |  |  |  |  |  |  |  |  |  | + |  |
| Wootton (2018). |  |  |  |  | + |  |  |  |  |  |  |  |  |  |  |  |  | + |  |
| ***Smartphone app-based program*** | | | | | | | | | | | | | | | | | | | |
| Bi (2021). |  |  |  |  |  |  |  |  |  |  |  |  |  |  |  |  |  |  |  |
| Cerdán-De-las-heras (2022). |  |  |  |  |  |  |  |  |  |  |  |  |  |  |  |  |  | + |  |
| Crooks (2020). |  |  |  |  |  | + |  |  |  |  |  |  |  |  |  |  |  |  |  |
| Jiang (2020). |  |  |  |  |  |  |  | + |  |  |  |  |  |  |  |  |  | + |  |
| Jimenez-Reguera (2020). |  |  |  | + |  | + |  |  |  |  | + |  |  |  |  |  |  | + |  |
| Loeckx (2023) |  |  |  |  | + |  |  |  |  |  |  |  |  |  |  |  |  |  |  |
| North (2020). |  |  |  |  | + |  |  |  |  |  |  |  |  |  |  |  |  | + |  |
| Park (2020). |  |  |  |  |  |  |  | + |  |  |  |  |  | + | + |  | + |  |  |
| Spielmanns (2023). |  |  |  |  | + |  |  |  |  |  |  |  |  |  |  |  |  |  |  |
| Wang (2021). |  |  |  |  |  |  |  |  |  |  |  |  |  |  |  |  |  |  |  |

^a^BCKQ : Bristol COPD Knowledge Questionnaire

^b^BMQ : Belief on Medication Questionnaire

^c^BPAQ : Baecke Physical Activity Questionnaire

^d^CAP-FISIO : A respiratory physiotherapy adherence self-report

^e^CRQ : Chronic Respiratory Disease Questionnaire

^f^EQoL-5D : Europe Quality of life-5 Dimension

^g^Exa self-efficacy : Exacerbation-related self-efficacy

^h^Ex-Self-Efficacy : Exercise-Self-Efficcacy

^i^MARS : Medication Adherence Rating Scale

^j^MLHRQ : Minnesota Living with Heart Failure Questionnaire

^k^PIH : Partners in Health (Self-Management)

^l^PRAISE : Pulmonary Rehabilitation Adapted Index of Self-Efficacy

^m^SCBI : Self-Care Behavior Inventory

^n^SEMCD : Self-Efficacy for Managing Chronic Disease 6-Item Scale

^o^SF-12 : 12-Item Short Form Health Survey

^p^SF-36 : 36-Item Short Form Health Survey

^q^SGRQ : St. George Respiratory Questionnaire

^r^Stanford SES : Stanford Self-efficacy Scale

This document is a supplementary appendix to a full article published in the Journal of Medical Internet Research (J Med Internet Res). For complete copyright and citation details, please refer to the main manuscript XXX

REFERENCES

38. Arbillaga-Etxarri A, Gimeno-Santos E, Barberan-Garcia A, et al. Long-term efficacy and effectiveness of a behavioural and community-based exercise intervention (urban training) to increase physical activity in patients with COPD: a randomised controlled trial. Eur Respir J. Oct 2018;52(4):1800063. [doi: 10.1183/13993003.00063-2018] [Medline: 30166322]

70. Benzo RP, Ridgeway J, Hoult JP, et al. Feasibility of a health coaching and home-based rehabilitation intervention with remote monitoring for COPD. Respir Care. Jun 2021;66(6):960-971. [doi: 10.4187/respcare.08580] [Medline: 33906954]

71. Benzo R, Hoult J, McEvoy C, et al. Promoting chronic obstructive pulmonary disease wellness through remote monitoring and health coaching: a clinical trial. Ann Am Thorac Soc. Nov 2022;19(11):1808-1817. [doi: 10.1513/AnnalsATS.202203-214OC] [Medline: 35914215]

41. Boer L, Bischoff E, van der Heijden M, et al. A smart mobile health tool versus a paper action plan to support self-management of chronic obstructive pulmonary disease exacerbations: randomized controlled trial. JMIR Mhealth Uhealth. Oct 9, 2019;7(10):e14408. [doi: 10.2196/14408] [Medline: 31599729]

42. Bourne S, DeVos R, North M, et al. Online versus face-to-face pulmonary rehabilitation for patients with chronic obstructive pulmonary disease: randomised controlled trial. BMJ Open. Jul 17, 2017;7(7):e014580. [doi: 10.1136/bmjopen-2016-014580] [Medline: 28716786]

57. Chan HY, Dai YT, Hou IC. Evaluation of a tablet-based instruction of breathing technique in patients with COPD. Int J Med Inform. Oct 2016;94(263-70):263-270. [doi: 10.1016/j.ijmedinf.2016.06.018] [Medline: 27573335]\

43. Farmer A, Williams V, Velardo C, et al. Self-management support using a digital health system compared with usual care for chronic obstructive pulmonary disease: randomized controlled trial. J Med Internet Res. May 3, 2017;19(5):e144. [doi: 10.2196/jmir.7116] [Medline: 28468749]

58. Ho TW, Huang CT, Chiu HC, et al. Effectiveness of telemonitoring in patients with chronic obstructive pulmonary disease in taiwan-a randomized controlled trial. Sci Rep. Mar 31, 2016;6(23797):23797. [doi: 10.1038/srep23797] [Medline: 27029815]

49. Kessler R, Casan-Clara P, Koehler D, et al. COMET: a multicomponent home-based disease-management programme versus routine care in severe COPD. Eur Respir J. Jan 2018;51(1):29326333. [doi: 10.1183/13993003.01612-2017] [Medline: 29326333]

67. Moy ML, Martinez CH, Kadri R, et al. Long-term effects of an internet-mediated pedometer-based walking program for chronic obstructive pulmonary disease: randomized controlled trial. J Med Internet Res. Aug 8, 2016;18(8):e215. [doi: 10.2196/jmir.5622] [Medline: 27502583]

44. Rixon L, Hirani SP, Cartwright M, et al. A RCT of telehealth for COPD patient’s quality of life: the whole system demonstrator evaluation. Clin Respir J. Jul 2017;11(4):459-469. [doi: 10.1111/crj.12359] [Medline: 26260325]

72. Robinson SA, Cooper JA Jr, Goldstein RL, et al. A randomised trial of a web-based physical activity self-management intervention in COPD. ERJ Open Res. Jul 2021;7(3):00158-2021. [doi: 10.1183/23120541.00158-2021] [Medline: 34476247]

50. Saleh S, Skeie S, Grundt H. Re-admission and quality of life among patients with chronic obstructive pulmonary disease after telemedicine video nursing consultation - a randomized study. Multidiscip Respir Med. Jan 17, 2023;18(1):918. [doi: 10.4081/mrm.2023.918] [Medline: 37753200]

73. Stamenova V, Liang K, Yang R, et al. Technology-enabled self-management of chronic obstructive pulmonary disease with or without asynchronous remote monitoring: randomized controlled trial. J Med Internet Res. Jul 30, 2020;22(7):e18598. [doi: 10.2196/18598] [Medline: 32729843]

55. Vasilopoulou M, Papaioannou AI, Kaltsakas G, et al. Home-based maintenance tele-rehabilitation reduces the risk for acute exacerbations of COPD, hospitalisations and emergency department visits. Eur Respir J. May 2017;49(5):1602129. [doi: 10.1183/13993003.02129-2016] [Medline: 28546268]

52. Vianello A, Fusello M, Gubian L, et al. Home telemonitoring for patients with acute exacerbation of chronic obstructive pulmonary disease: a randomized controlled trial. BMC Pulm Med. Nov 22, 2016;16(1):157. [doi: 10.1186/s12890-016-0321-2] [Medline: 27876029]

45. Walker PP, Pompilio PP, Zanaboni P, et al. Telemonitoring in Chronic Obstructive Pulmonary Disease (CHROMED). A Randomized Clinical Trial. Am J Respir Crit Care Med. Sep 1, 2018;198(5):620-628. [doi: 10.1164/rccm.201712-2404OC] [Medline: 29557669]

68. Wan ES, Kantorowski A, Homsy D, et al. Promoting physical activity in COPD: insights from a randomized trial of a web-based intervention and pedometer use. Respir Med. Sep 2017;130(102-10):102-110. [doi: 10.1016/j.rmed.2017.07.057] [Medline: 29206627]

69. Wan ES, Kantorowski A, Polak M, et al. Long-term effects of web-based pedometer-mediated intervention on COPD exacerbations. Respir Med. Feb 2020;162:105878. [doi: 10.1016/j.rmed.2020.105878] [Medline: 32056676]

59. Wang L, He L, Tao Y, et al. Evaluating a web-based coaching program using electronic health records for patients with chronic obstructive pulmonary disease in China: randomized controlled trial. J Med Internet Res. Jul 21, 2017;19(7):e264. [doi: 10.2196/jmir.6743] [Medline: 28733270]

51. Zanaboni P, Dinesen B, Hoaas H, et al. Long-term telerehabilitation or unsupervised training at home for patients with chronic obstructive pulmonary disease: a randomized controlled trial. Am J Respir Crit Care Med. Apr 1, 2023;207(7):865-875. [doi: 10.1164/rccm.202204-0643OC] [Medline: 36480957]

46. Jolly K, Sidhu MS, Hewitt CA, et al. Self management of patients with mild COPD in primary care: randomised controlled trial. BMJ. Jun 13, 2018;361:k2241. [doi: 10.1136/bmj.k2241] [Medline: 29899047]

39. Varas AB, Córdoba S, Rodríguez-Andonaegui I, Rueda MR, García-Juez S, Vilaró J. Effectiveness of a community-based exercise training programme to increase physical activity level in patients with chronic obstructive pulmonary disease: a randomized controlled trial. Physiother Res Int. Oct 2018;23(4):e1740. [doi: 10.1002/pri.1740] [Medline: 30168228]

66. Wootton SL, McKeough Z, Ng CLW, et al. Effect on health-related quality of life of ongoing feedback during a 12-month maintenance walking programme in patients with COPD: a randomized controlled trial. Respirology. Jan 2018;23(1):60-67. [doi: 10.1111/resp.13128] [Medline: 28758320]

60. Bi J, Yang W, Hao P, et al. WeChat as a platform for baduanjin intervention in patients with stable chronic obstructive pulmonary disease in China: retrospective randomized controlled trial. JMIR Mhealth Uhealth. Feb 2, 2021;9(2):e23548. [doi: 10.2196/23548] [Medline: 33528369]

53. Cerdán-de-las-Heras J, Balbino F, Løkke A, Catalán-Matamoros D, Hilberg O, Bendstrup E. Effect of a new tele-rehabilitation program versus standard rehabilitation in patients with chronic obstructive pulmonary disease. JCM. 2022;11(1):11. [doi: 10.3390/jcm11010011]

47. Crooks MG, Elkes J, Storrar W, et al. Evidence generation for the clinical impact of myCOPD in patients with mild, moderate and newly diagnosed COPD: a randomised controlled trial. ERJ Open Res. Oct 2020;6(4):1-10. [doi: 10.1183/23120541.00460-2020] [Medline: 33263052]

61. Jiang Y, Liu F, Guo J, et al. Evaluating an intervention program using WeChat for patients with chronic obstructive pulmonary disease: randomized controlled trial. J Med Internet Res. Apr 21, 2020;22(4):e17089. [doi: 10.2196/17089] [Medline: 32314971]

40. Jiménez-Reguera B, Maroto López E, Fitch S, et al. Development and preliminary evaluation of the effects of an mHealth web-based platform (HappyAir) on adherence to a maintenance program after pulmonary rehabilitation in patients with chronic obstructive pulmonary disease: randomized controlled trial. JMIR Mhealth Uhealth. Jul 31, 2020;8(7):e18465. [doi: 10.2196/18465] [Medline: 32513646]

56. Loeckx M, Rodrigues FM, Blondeel A, et al. Sustaining training effects through physical activity coaching (STEP): a randomized controlled trial. Int J Behav Nutr Phys Act. Oct 10, 2023;20(1):121. [doi: 10.1186/s12966-023-01519-w] [Medline: 37814266]

48. North M, Bourne S, Green B, et al. A randomised controlled feasibility trial of E-health application supported care vs usual care after exacerbation of COPD: the RESCUE trial. NPJ Digit Med. 2020;3(1):145. [doi: 10.1038/s41746-020-00347-7] [Medline: 33145441]

63. Park SK, Bang CH, Lee SH. Evaluating the effect of a smartphone app-based self-management program for people with COPD: a randomized controlled trial. Appl Nurs Res. Apr 2020;52:151231. [doi: 10.1016/j.apnr.2020.151231] [Medline: 31955942]

54. Spielmanns M, Gloeckl R, Jarosch I, et al. Using a smartphone application maintains physical activity following pulmonary rehabilitation in patients with COPD: a randomised controlled trial. Thorax. May 2023;78(5):442-450. [doi: 10.1136/thoraxjnl-2021-218338] [Medline: 35450945]

62. Wang L, Guo Y, Wang M, Zhao Y. A mobile health application to support self-management in patients with chronic obstructive pulmonary disease: a randomised controlled trial. Clin Rehabil. Jan 2021;35(1):90-101. [doi: 10.1177/0269215520946931]ss
