# Supplementary material for: Effectiveness of Mobile Health–Based Self-Management Programs on Health-Related Outcomes in Patients With Chronic Obstructive Pulmonary Disease: Systematic Review and Meta-Analysis
Source: JMIR Mhealth Uhealth. 2025 Dec 29;13:e74967. doi: 10.2196/74967 (PMC12747663; doi:10.2196/74967)
Supplement: Multimedia Appendix 4 [file mhealth-v13-e74967-s004.docx]

**Table S6**

| Author (year) | Before intervention | | | | After intervention | | | |
| --- | --- | --- | --- | --- | --- | --- | --- | --- |
|  | IG^a^ | | CG^b^ | | IG | | CG | |
|  | n | Mean (SD^c^) | n | Mean (SD) | n | Mean (SD) | n | Mean (SD) |
| ***Dyspnea: mMRC*^d^** | | | | | | | | |
| Benzo (2022) | 188 | 2.7 (0.8) | 187 | 2.6 (0.9) | 188 | 2.5 (1.0) | 187 | 2.7 (0.8) |
| Bourne (2017) | 64 | 2.0 (2.0) | 26 | 1.0 (1.0) | 64 | 1.0 (1.0) | 26 | 1.5 (1.0) |
| Jiang (2020) | 53 | 2.8 (0.7) | 53 | 2.8 (0.7) | 53 | 2.4 (0.8) | 53 | 2.4 (0.7) |
| North (2020) | 20 | 2.9 (1.3) | 21 | 3.1 (1.1) | 20 | 2.8 (1.4) | 21 | 2.8 (1.1) |
| Robinson (2021) | 75 | 2.0 (1.2) | 78 | 2.2 (1.2) | 75 | 1.9 (1.8) | 78 | 2.1 (1.9) |
| Tsai (2017) [34] | 20 | 4.0 (2.0) | 17 | 4.0 (2.0) | 19 | 3.0 (2.0) | 17 | 4.0 (2.0) |
| Varas (2018) [9] | 21 | 1.4 (0.8) | 19 | 1.5 (0.7) | 17 | 1.2 (0.4) | 16 | 1.5 (0.6) |
| Vasilopoulou (2017) | 47 | 2.3 (1.0) | 50 | 2.2 (1.1) | 47 | 1.6 (1.0) | 50 | 3.1 (0.8) |
| Wang(2017) | 55 | 2.3 (1.0) | 65 | 2.5 (0.9) | 55 | 1.1 (1.0) | 65 | 2.9 (0.8) |
| Zanaboni (2023) | 40 | 2.1 (1.0) | 40 | 1.9 (1.1) | 40 | 1.9 (1.2) | 40 | 2.3 (1.1) |
| ***Physical activity: 6MWT*^e^** | | | | | | | | |
| Arbillaga (2017) | 132 | 499.0 (95.0) | 148 | 501.0 (83.0) | 132 | 488.0 (106.0) | 148 | 493.0 (90.0) |
| Bourne (2017) | 64 | 388.7 (104.4) | 26 | 416.5 (118.3) | 64 | 433.6 (102.9) | 26 | 445.1 (124.9) |
| Cerdán-De-las-heras (2022) | 27 | 385.6 (86.9) | 27 | 366.6 (97.8) | 18 | 434.6 (84.5) | 17 | 387.2 (100.9) |
| Jimenez-Reguera (2020) | 17 | 336.2 (76.7) | 19 | 333.8 (69.2) | 17 | 357.4 (112.5) | 19 | 339.9 (75.9) |
| Kessler (2018) | 157 | 278.0 (115.0) | 162 | 292.0 (121.0) | 157 | 300.5 (96.6) | 162 | 280.0 (120.4) |
| Loeckx (2023) | 36 | 512.0 (79.0) | 37 | 499.0 (72.0) | 36 | 492.0 (-) | 37 | 473.0 (-) |
| Park (2020) | 22 | 378.3 (97.0) | 20 | 398.1 (78.7) | 22 | 433.2 (107.2) | 20 | 437.6 (83.6) |
| Robinson (2021) | 75 | 360.8 (92.0) | 78 | 357.2 (103.5) | 75 | 385.9 (83.2) | 78 | 394.6 (85.0) |
| Tsai (2017) | 19 | 363.0 (66.0) | 17 | 383.0 (93.0) | 19 | 403.0 (82.0) | 17 | 374.0 (136.0) |
| Vasilopoulou (2017) | 47 | 389.1 (91.3) | 50 | 384.8 (80.2) | 47 | 420.2 (74.9) | 50 | 339.9 (110.1) |
| Wan (2017) | 57 | 382.5 (89.4) | 52 | 394.2 (71.8) | 57 | 381.6 (55.8) | 52 | 396.8 (46.7) |
| Wang (2017) | 55 | 267.4 (121.1) | 65 | 224.1 (102.3) | 55 | 297.3 (113.2) | 65 | 198.9 (98.8) |
| Wootton (2018) | 49 | 458.0 (87.0) | 46 | 467.0 (80.0) | 35 | 469.0 (98.0) | 27 | 432.0 (121.0) |
| Zanaboni (2023) | 40 | 367 (125) | 40 | 384 (111) | 40 | 400 (142) | 40 | 357 (102) |
| ***QoL*^f^*: SGRQ*^g^** | | | | | | | | |
| Bourne (2017) | 64 | 42.4 (18.6) | 26 | 37.7 (17.2) | 64 | 39.3 (18.5) | 26 | 39.3 (18.5) |
| Cerdán-De-las-heras (2022) | 27 | 55.6 (13.5) | 27 | 60.6 (14.1) | 18 | 51.9 (10.6) | 14 | 54.0 (16.3) |
| Farmer (2017) | 110 | 56.4 (19.7) | 56 | 55.5 (16.2) | 93 | 56.9 (19.5) | 48 | 56.8 (20.9) |
| Jiang (2020) | 53 | 50.2 (21.0) | 53 | 49.6 (17.5) | 53 | 39.7 (20.9) | 53 | 44.2 (19.9) |
| Jimenez-Reguera (2020 | 17 | 53.2 (12.1) | 19 | 53.1 (14.8) | 17 | 45.7 (19.8) | 19 | 46.8 (14.0) |
| Jolly (2018) | 277 | 27.8 (14.6) | 272 | 29.5 (14.5) | 217 | 27.9 (15.7) | 256 | 30.9 (17.0) |
| Kessler (2018) | 157 | 52.2 (19.7) | 162 | 57.4 (18.3) | 157 | 55.9 (20.2) | 162 | 56.5 (19.9) |
| Moy (2016) | 154 | 45.6 (15.4) | 84 | 46.8 (15.6) | 154 | 43.1 (12.3) | 84 | 45.4 (12.6) |
| North (2020) | 20 | 66.4 (16.6) | 21 | 68.1 (13.7) | 20 | 61.9 (14.9) | 21 | 64.1 (15.9) |
| Robinson (2021) | 75 | 40.0 (15.3) | 78 | 38.0 (17.8) | 75 | 27.0 (31.1) | 78 | 22.9 (28.4) |
| Varas (2018) | 21 | 41.2 (13.2) | 19 | 44.6 (15.6) | 17 | 36.8 (3.6) | 16 | 45.6 (4.3) |
| Vasilopoulou (2017) | 47 | 46.2 (19.7) | 50 | 44.1 (16.6) | 47 | 38.4 (20.5) | 50 | 50.2 (17.7) |
| Wan (2017) | 57 | 34.9 (16.8) | 52 | 31.9 (15.9) | 57 | 34.0 (9.9) | 52 | 31.2 (12.7) |
| Wang (2017) | 55 | 47.2 (18.7) | 65 | 51.2 (18.5) | 55 | 31.4 (20.5) | 65 | 57.9 (21.4) |
| Wootton (2018) | 49 | 46.0 (18.0) | 46 | 47.0 (16.0) | 38 | 40.0 (17.0) | 33 | 47.0 (17.0) |
|  |  |  |  |  |  |  |  |  |

^a^IG: intervention group; ^b^CG: control group; ^c^SD: standard deviation; ^d^mMRC: modified scale of the Medical Research Council; ^e^6MWT: 6-Minutes Walking Test; ^f^QoL: quality of life; ^g^SGRQ: Saint George Respiratory Questionnaire

This document is a supplementary appendix to a full article published in the Journal of Medical Internet Research (J Med Internet Res). For complete copyright and citation details, please refer to the main manuscript.

REFERENCES

38. Arbillaga-Etxarri A, Gimeno-Santos E, Barberan-Garcia A, et al. Long-term efficacy and effectiveness of a behavioural and community-based exercise intervention (urban training) to increase physical activity in patients with COPD: a randomised controlled trial. Eur Respir J. Oct 2018;52(4):1800063. [doi: 10.1183/13993003.00063-2018] [Medline: 30166322]

70. Benzo RP, Ridgeway J, Hoult JP, et al. Feasibility of a health coaching and home-based rehabilitation intervention with remote monitoring for COPD. Respir Care. Jun 2021;66(6):960-971. [doi: 10.4187/respcare.08580] [Medline: 33906954]

71. Benzo R, Hoult J, McEvoy C, et al. Promoting chronic obstructive pulmonary disease wellness through remote monitoring and health coaching: a clinical trial. Ann Am Thorac Soc. Nov 2022;19(11):1808-1817. [doi: 10.1513/AnnalsATS.202203-214OC] [Medline: 35914215]

41. Boer L, Bischoff E, van der Heijden M, et al. A smart mobile health tool versus a paper action plan to support self-management of chronic obstructive pulmonary disease exacerbations: randomized controlled trial. JMIR Mhealth Uhealth. Oct 9, 2019;7(10):e14408. [doi: 10.2196/14408] [Medline: 31599729]

42. Bourne S, DeVos R, North M, et al. Online versus face-to-face pulmonary rehabilitation for patients with chronic obstructive pulmonary disease: randomised controlled trial. BMJ Open. Jul 17, 2017;7(7):e014580. [doi: 10.1136/bmjopen-2016-014580] [Medline: 28716786]

57. Chan HY, Dai YT, Hou IC. Evaluation of a tablet-based instruction of breathing technique in patients with COPD. Int J Med Inform. Oct 2016;94(263-70):263-270. [doi: 10.1016/j.ijmedinf.2016.06.018] [Medline: 27573335]\

43. Farmer A, Williams V, Velardo C, et al. Self-management support using a digital health system compared with usual care for chronic obstructive pulmonary disease: randomized controlled trial. J Med Internet Res. May 3, 2017;19(5):e144. [doi: 10.2196/jmir.7116] [Medline: 28468749]

58. Ho TW, Huang CT, Chiu HC, et al. Effectiveness of telemonitoring in patients with chronic obstructive pulmonary disease in taiwan-a randomized controlled trial. Sci Rep. Mar 31, 2016;6(23797):23797. [doi: 10.1038/srep23797] [Medline: 27029815]

49. Kessler R, Casan-Clara P, Koehler D, et al. COMET: a multicomponent home-based disease-management programme versus routine care in severe COPD. Eur Respir J. Jan 2018;51(1):29326333. [doi: 10.1183/13993003.01612-2017] [Medline: 29326333]

67. Moy ML, Martinez CH, Kadri R, et al. Long-term effects of an internet-mediated pedometer-based walking program for chronic obstructive pulmonary disease: randomized controlled trial. J Med Internet Res. Aug 8, 2016;18(8):e215. [doi: 10.2196/jmir.5622] [Medline: 27502583]

44. Rixon L, Hirani SP, Cartwright M, et al. A RCT of telehealth for COPD patient’s quality of life: the whole system demonstrator evaluation. Clin Respir J. Jul 2017;11(4):459-469. [doi: 10.1111/crj.12359] [Medline: 26260325]

72. Robinson SA, Cooper JA Jr, Goldstein RL, et al. A randomised trial of a web-based physical activity self-management intervention in COPD. ERJ Open Res. Jul 2021;7(3):00158-2021. [doi: 10.1183/23120541.00158-2021] [Medline: 34476247]

50. Saleh S, Skeie S, Grundt H. Re-admission and quality of life among patients with chronic obstructive pulmonary disease after telemedicine video nursing consultation - a randomized study. Multidiscip Respir Med. Jan 17, 2023;18(1):918. [doi: 10.4081/mrm.2023.918] [Medline: 37753200]

73. Stamenova V, Liang K, Yang R, et al. Technology-enabled self-management of chronic obstructive pulmonary disease with or without asynchronous remote monitoring: randomized controlled trial. J Med Internet Res. Jul 30, 2020;22(7):e18598. [doi: 10.2196/18598] [Medline: 32729843]

55. Vasilopoulou M, Papaioannou AI, Kaltsakas G, et al. Home-based maintenance tele-rehabilitation reduces the risk for acute exacerbations of COPD, hospitalisations and emergency department visits. Eur Respir J. May 2017;49(5):1602129. [doi: 10.1183/13993003.02129-2016] [Medline: 28546268]

52. Vianello A, Fusello M, Gubian L, et al. Home telemonitoring for patients with acute exacerbation of chronic obstructive pulmonary disease: a randomized controlled trial. BMC Pulm Med. Nov 22, 2016;16(1):157. [doi: 10.1186/s12890-016-0321-2] [Medline: 27876029]

45. Walker PP, Pompilio PP, Zanaboni P, et al. Telemonitoring in Chronic Obstructive Pulmonary Disease (CHROMED). A Randomized Clinical Trial. Am J Respir Crit Care Med. Sep 1, 2018;198(5):620-628. [doi: 10.1164/rccm.201712-2404OC] [Medline: 29557669]

68. Wan ES, Kantorowski A, Homsy D, et al. Promoting physical activity in COPD: insights from a randomized trial of a web-based intervention and pedometer use. Respir Med. Sep 2017;130(102-10):102-110. [doi: 10.1016/j.rmed.2017.07.057] [Medline: 29206627]

69. Wan ES, Kantorowski A, Polak M, et al. Long-term effects of web-based pedometer-mediated intervention on COPD exacerbations. Respir Med. Feb 2020;162:105878. [doi: 10.1016/j.rmed.2020.105878] [Medline: 32056676]

59. Wang L, He L, Tao Y, et al. Evaluating a web-based coaching program using electronic health records for patients with chronic obstructive pulmonary disease in China: randomized controlled trial. J Med Internet Res. Jul 21, 2017;19(7):e264. [doi: 10.2196/jmir.6743] [Medline: 28733270]

51. Zanaboni P, Dinesen B, Hoaas H, et al. Long-term telerehabilitation or unsupervised training at home for patients with chronic obstructive pulmonary disease: a randomized controlled trial. Am J Respir Crit Care Med. Apr 1, 2023;207(7):865-875. [doi: 10.1164/rccm.202204-0643OC] [Medline: 36480957]

46. Jolly K, Sidhu MS, Hewitt CA, et al. Self management of patients with mild COPD in primary care: randomised controlled trial. BMJ. Jun 13, 2018;361:k2241. [doi: 10.1136/bmj.k2241] [Medline: 29899047]

39. Varas AB, Córdoba S, Rodríguez-Andonaegui I, Rueda MR, García-Juez S, Vilaró J. Effectiveness of a community-based exercise training programme to increase physical activity level in patients with chronic obstructive pulmonary disease: a randomized controlled trial. Physiother Res Int. Oct 2018;23(4):e1740. [doi: 10.1002/pri.1740] [Medline: 30168228]

66. Wootton SL, McKeough Z, Ng CLW, et al. Effect on health-related quality of life of ongoing feedback during a 12-month maintenance walking programme in patients with COPD: a randomized controlled trial. Respirology. Jan 2018;23(1):60-67. [doi: 10.1111/resp.13128] [Medline: 28758320]

60. Bi J, Yang W, Hao P, et al. WeChat as a platform for baduanjin intervention in patients with stable chronic obstructive pulmonary disease in China: retrospective randomized controlled trial. JMIR Mhealth Uhealth. Feb 2, 2021;9(2):e23548. [doi: 10.2196/23548] [Medline: 33528369]

53. Cerdán-de-las-Heras J, Balbino F, Løkke A, Catalán-Matamoros D, Hilberg O, Bendstrup E. Effect of a new tele-rehabilitation program versus standard rehabilitation in patients with chronic obstructive pulmonary disease. JCM. 2022;11(1):11. [doi: 10.3390/jcm11010011]

47. Crooks MG, Elkes J, Storrar W, et al. Evidence generation for the clinical impact of myCOPD in patients with mild, moderate and newly diagnosed COPD: a randomised controlled trial. ERJ Open Res. Oct 2020;6(4):1-10. [doi: 10.1183/23120541.00460-2020] [Medline: 33263052]

61. Jiang Y, Liu F, Guo J, et al. Evaluating an intervention program using WeChat for patients with chronic obstructive pulmonary disease: randomized controlled trial. J Med Internet Res. Apr 21, 2020;22(4):e17089. [doi: 10.2196/17089] [Medline: 32314971]

40. Jiménez-Reguera B, Maroto López E, Fitch S, et al. Development and preliminary evaluation of the effects of an mHealth web-based platform (HappyAir) on adherence to a maintenance program after pulmonary rehabilitation in patients with chronic obstructive pulmonary disease: randomized controlled trial. JMIR Mhealth Uhealth. Jul 31, 2020;8(7):e18465. [doi: 10.2196/18465] [Medline: 32513646]

56. Loeckx M, Rodrigues FM, Blondeel A, et al. Sustaining training effects through physical activity coaching (STEP): a randomized controlled trial. Int J Behav Nutr Phys Act. Oct 10, 2023;20(1):121. [doi: 10.1186/s12966-023-01519-w] [Medline: 37814266]

48. North M, Bourne S, Green B, et al. A randomised controlled feasibility trial of E-health application supported care vs usual care after exacerbation of COPD: the RESCUE trial. NPJ Digit Med. 2020;3(1):145. [doi: 10.1038/s41746-020-00347-7] [Medline: 33145441]

63. Park SK, Bang CH, Lee SH. Evaluating the effect of a smartphone app-based self-management program for people with COPD: a randomized controlled trial. Appl Nurs Res. Apr 2020;52:151231. [doi: 10.1016/j.apnr.2020.151231] [Medline: 31955942]

54. Spielmanns M, Gloeckl R, Jarosch I, et al. Using a smartphone application maintains physical activity following pulmonary rehabilitation in patients with COPD: a randomised controlled trial. Thorax. May 2023;78(5):442-450. [doi: 10.1136/thoraxjnl-2021-218338] [Medline: 35450945]

62. Wang L, Guo Y, Wang M, Zhao Y. A mobile health application to support self-management in patients with chronic obstructive pulmonary disease: a randomised controlled trial. Clin Rehabil. Jan 2021;35(1):90-101. [doi: 10.1177/0269215520946931]ss
