# Supplementary material for: Effectiveness of Mobile Health–Based Self-Management Programs on Health-Related Outcomes in Patients With Chronic Obstructive Pulmonary Disease: Systematic Review and Meta-Analysis
Source: JMIR Mhealth Uhealth. 2025 Dec 29;13:e74967. doi: 10.2196/74967 (PMC12747663; doi:10.2196/74967)
Supplement: Multimedia Appendix 5 [file mhealth-v13-e74967-s005.docx]

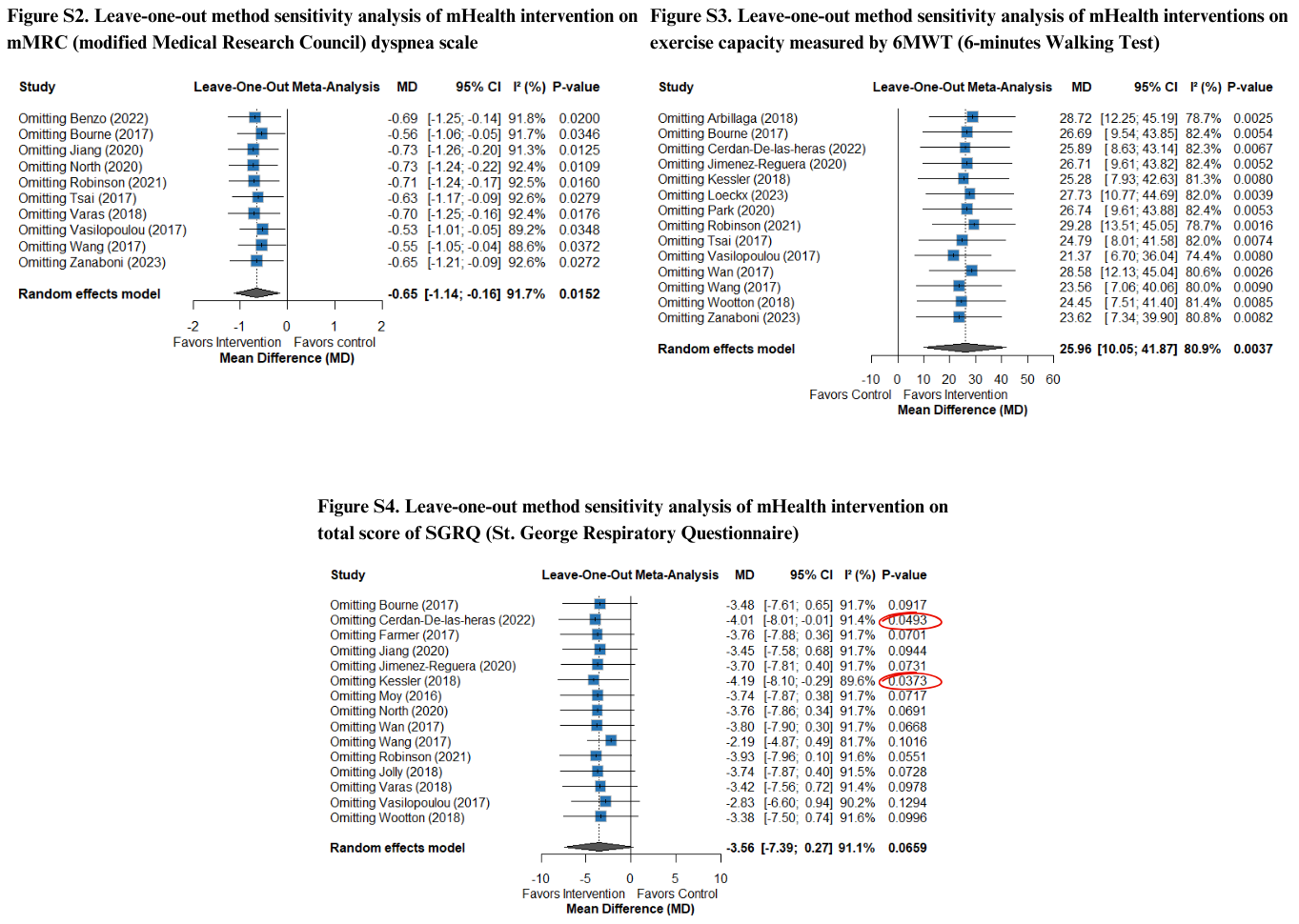


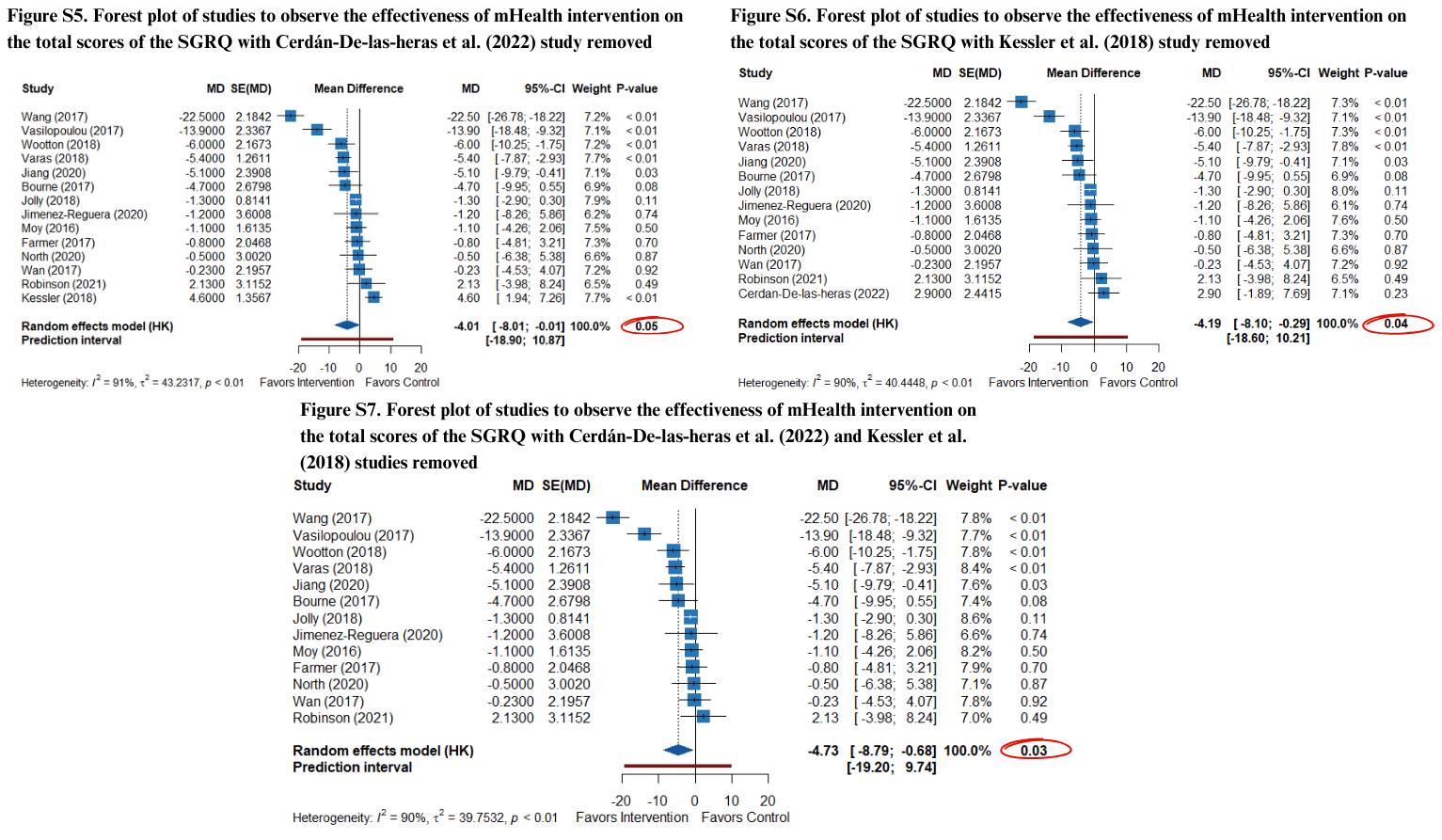

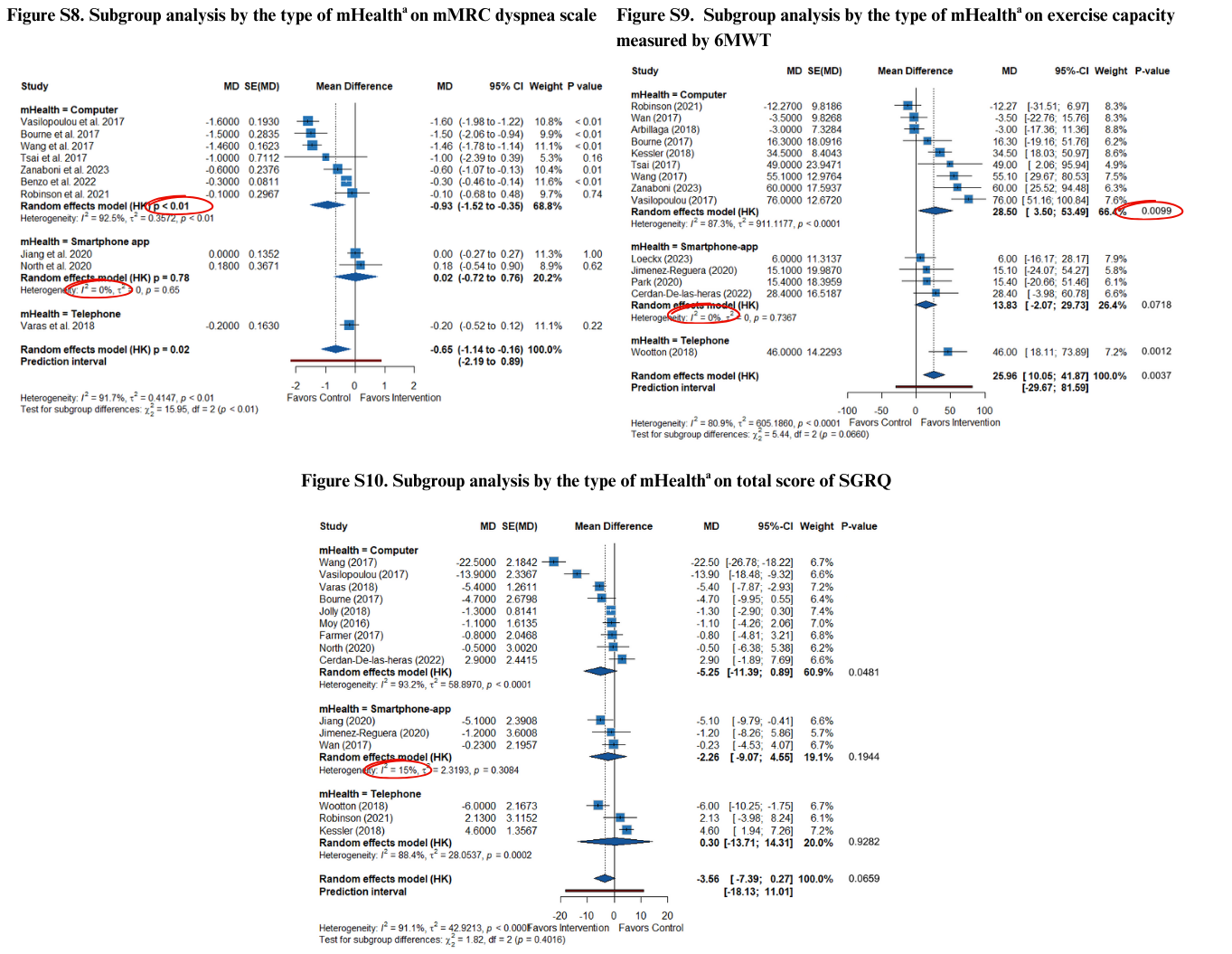


^a^Subgroup classifications of the type of mHealth are: computer, smartphone-app (application), and telephone.
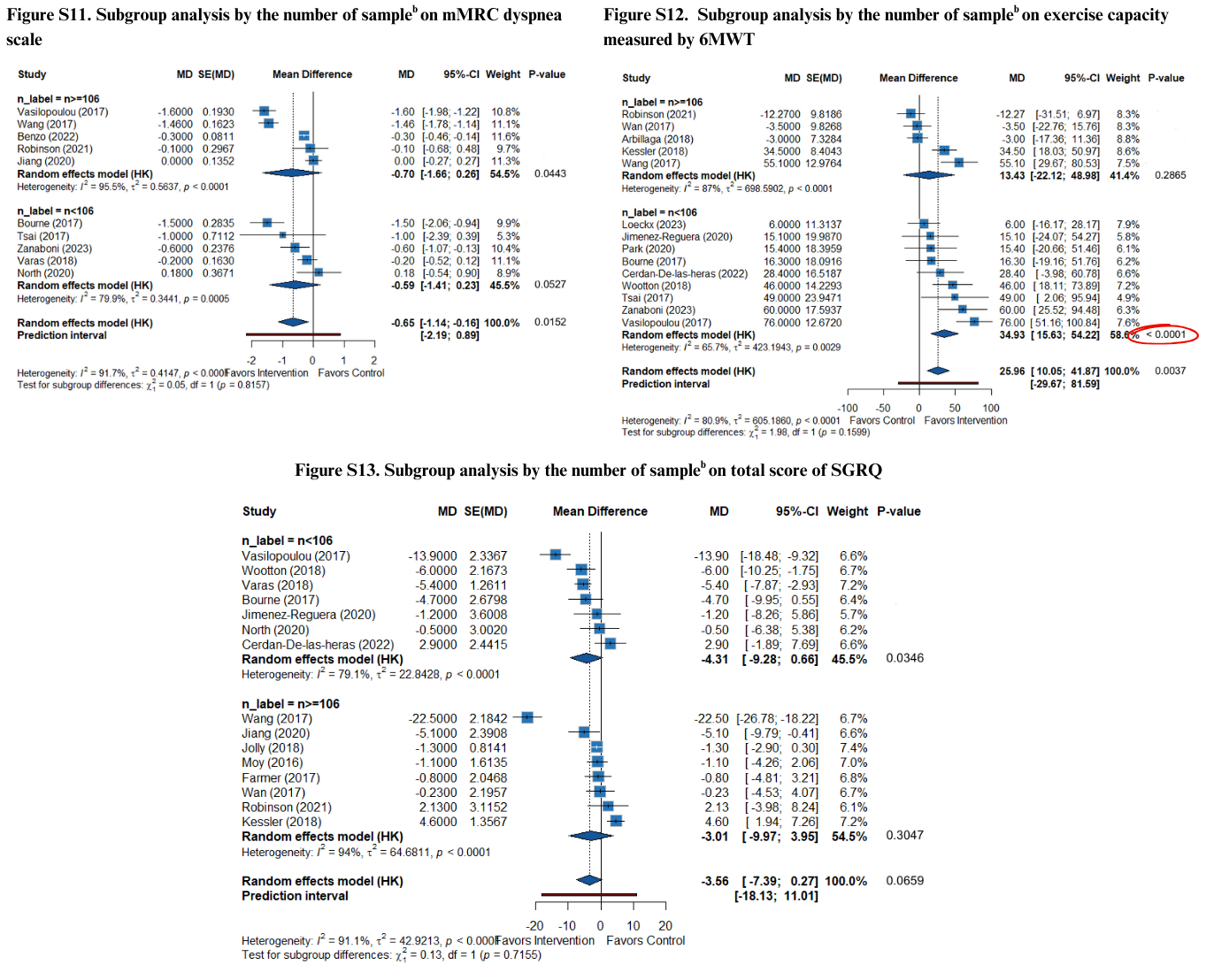


^b^Subgroup classifications of the number of sample are: n<106 and n≥106 (the median value of the total sample size for each study).


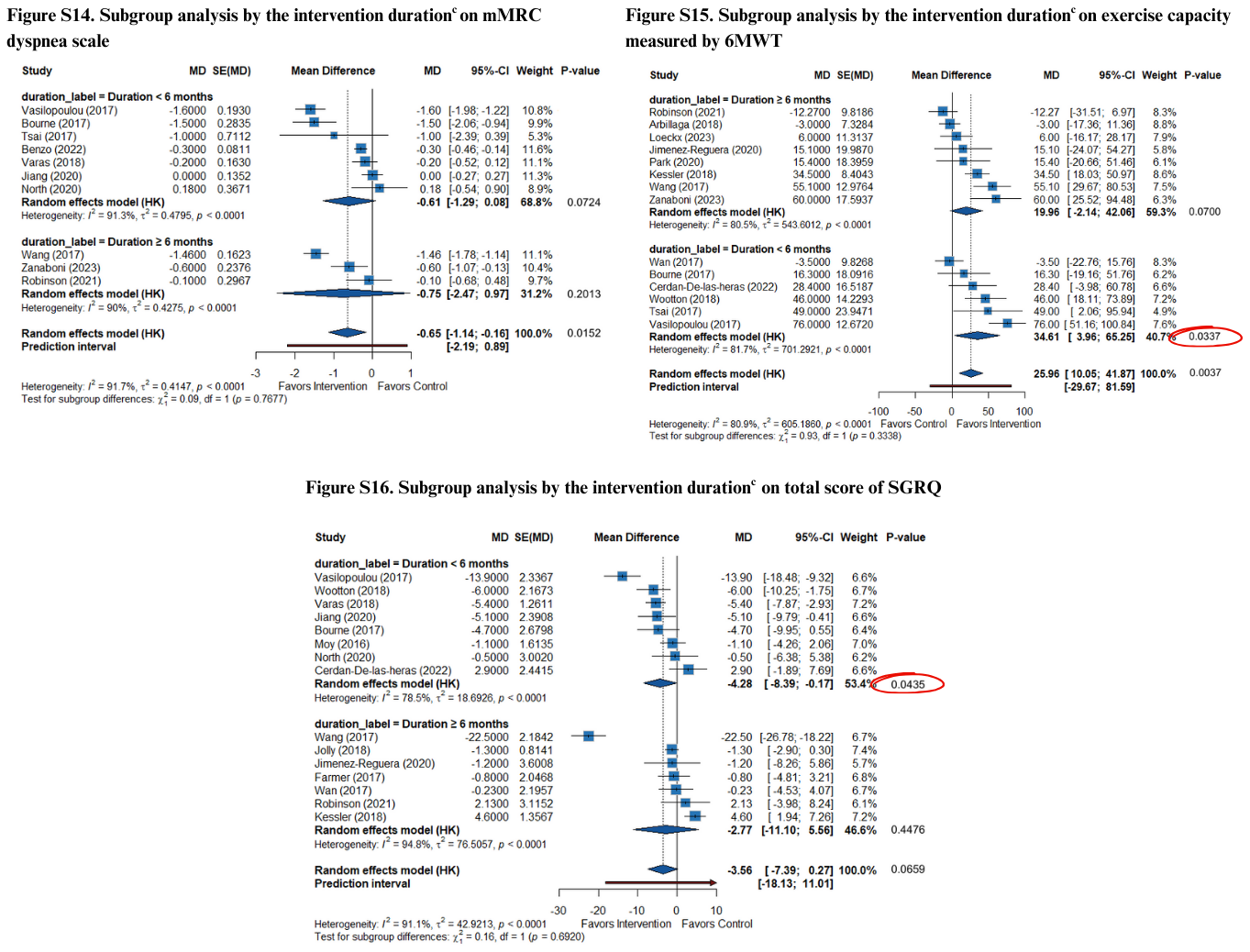


^c^Subgroup classifications of the intervention duration are: <6 months and ≥6 months (short and long period of intervention).


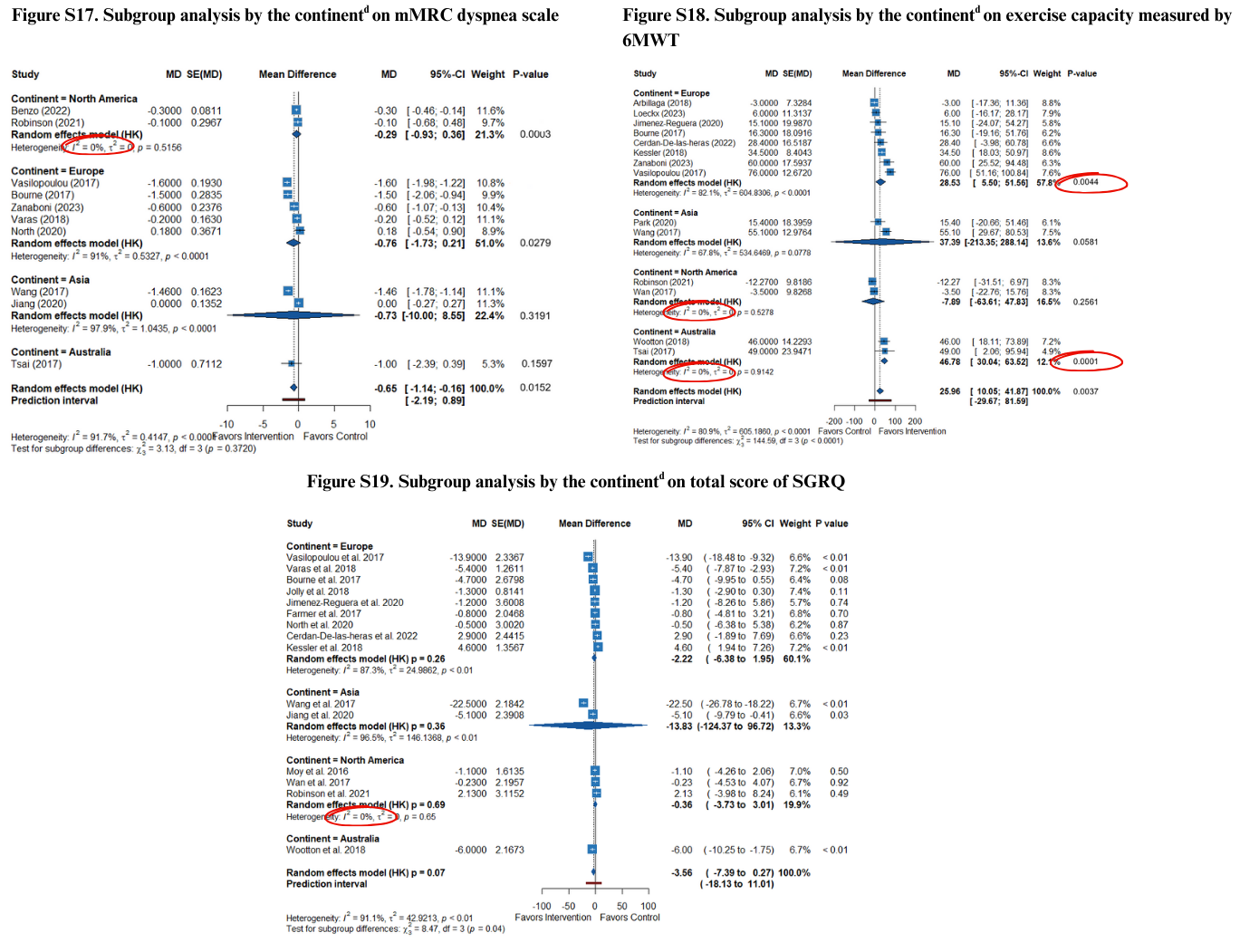


^d^Subgroup classifications of the continent are: Asia, Australia, Europe and North America


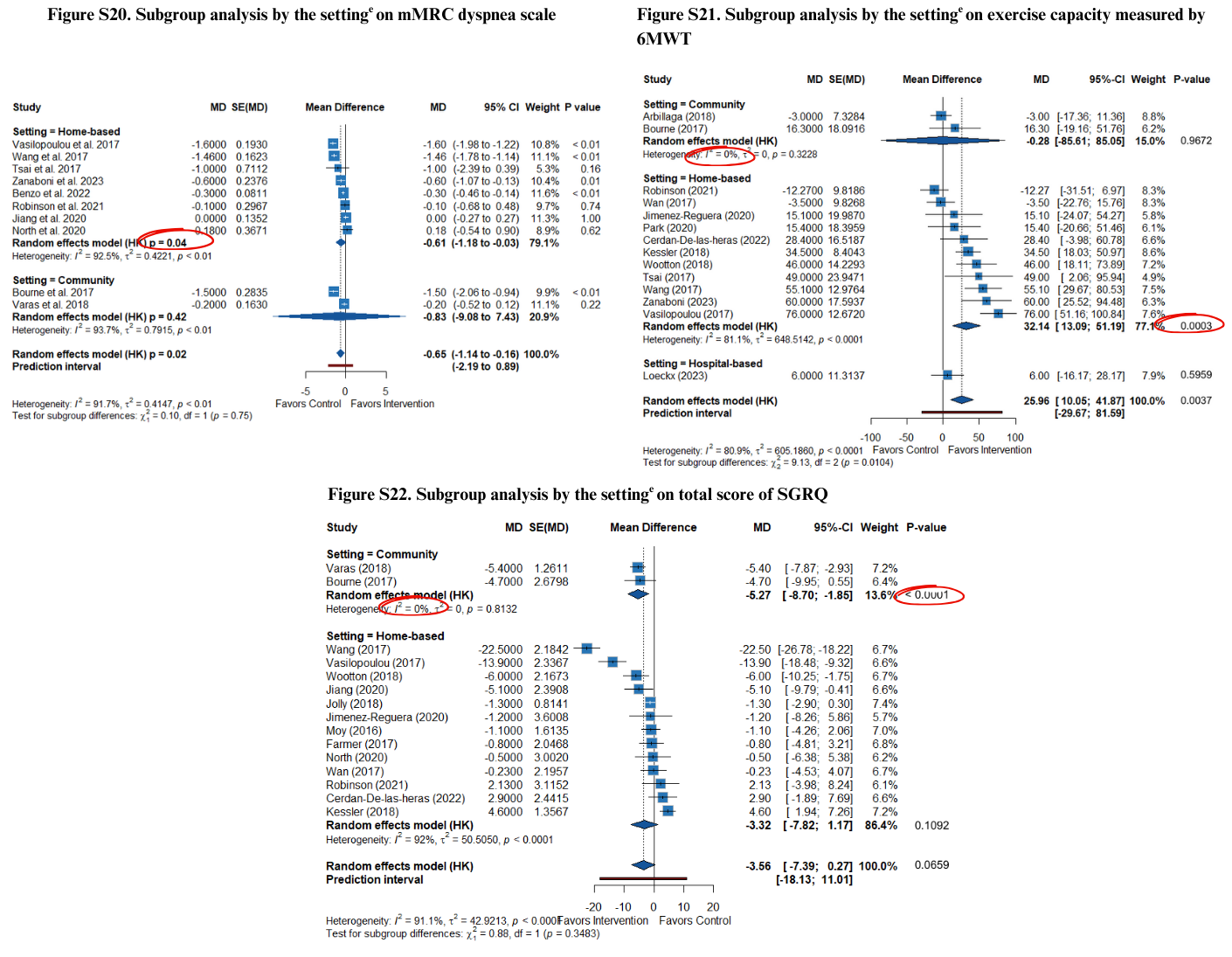


^e^Subgroup classifications of the setting are: community, home-based and hospital-based

^
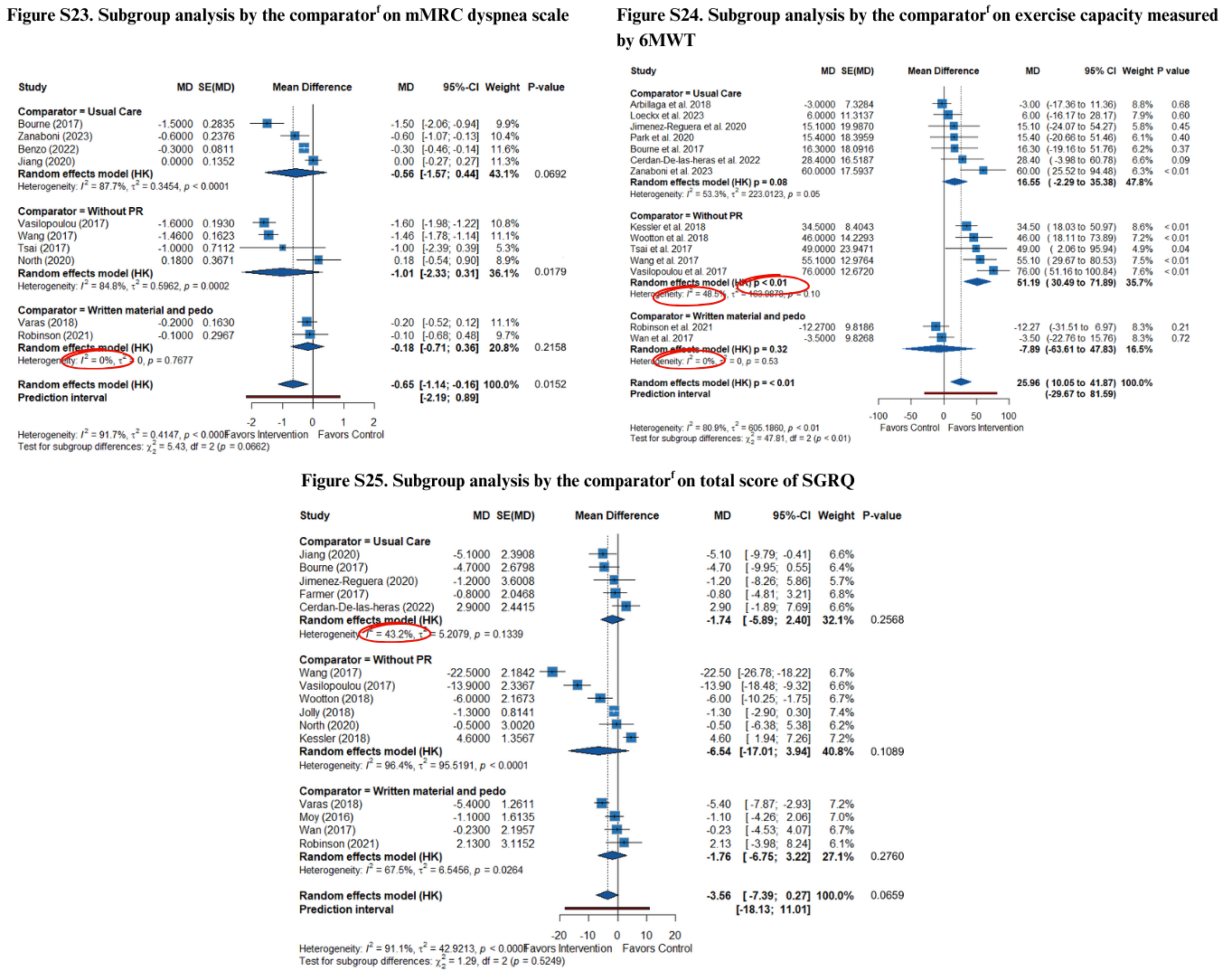
^

^f^Subgroup classifications of the comparator are: “usual care”, “without PR (Pulmonary Rehabilitation)” and “written material and pedometer”

^
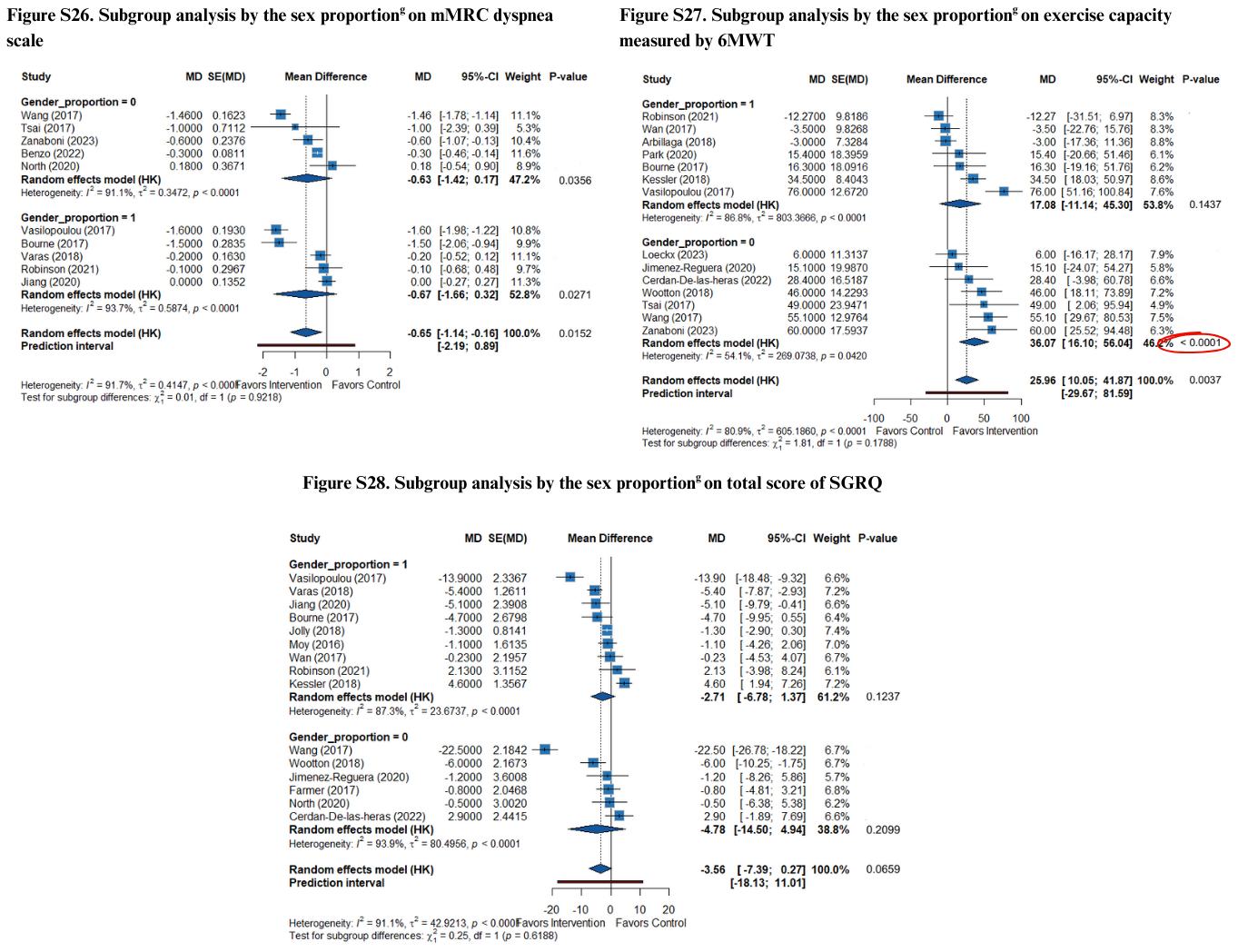
^

^g^Subgroup classifications of the sex proportion are: male percentage <62.75% (0) and male percentage ≥62.75% (1) (62.75% represents the median proportion of male participants in each of included studies).**Table S7.** Summary Finding of Subgroup analysis of the effectiveness of mHealth intervention on mMRC dyspnea score, 6MWT and QoL (total score of SGRQ)

| Subgroup | Number of Studies | Mean Difference | 95% CI | *P-value* of the main outcome | Heterogeneity Test results I^2^ (%) | *P-value* of the heterogeneity | Meta Regression (p) | Effects Model |
| --- | --- | --- | --- | --- | --- | --- | --- | --- |
| ***mMRC dyspnea score*** | | | | | | | | |
| *Type of mHealth intervention* | | | | | | | | |
| Computer | 7 | -0.93 | -1.52-(-0.35) | <.01^a^ | 93% | <.01 | **.09^c^** | Random effects model |
| Smartphone app | 2 | 0.02 | -0.72-0.76 | .78 | 0%^b^ | .65 |  | Random effects model |
| Telephone | 1 | -0.2 | -0.52-0.12 | .22 | / | / |  | / |
| *The total number of samples* | | | | | | | | |
| n<106 | 5 | -0.59 | -1.41-0.23 | .05 | 79.9% | <.01 | .83 | Random effects model |
| n≥106 | 5 | -0.70 | -1.66-0.26 | .04 | 95.5% | <.01 |  | Random effects model |
| *Duration of Intervention* | | | | | | | | |
| <6 months | 7 | -0.61 | -1.29-0.08 | .07 | 91.3% | <.01 | .78 | Random effects model |
| ≥6 months | 3 | -0.75 | -2.47-0.97 | .20 | 90% | <.01 |  | Random effects model |
| *Continent* |  |  |  |  |  |  |  |  |
| Asia | 2 | -0.73 | -10.00-8.55 | .32 | 97.9% | <.01 | .82 | Random effects model |
| Australia | 1 | -1.00 | -2.39-0.39 | .16 | / | / |  |  |
| Europe | 5 | -0.76 | -1.73-0.21 | .03 | 91% | <.01 |  | Random effects model |
| North America | 2 | -0.29 | -0.93-0.36 | <.01 | 0%^b^ | .516 |  | Random effects model |
| *Comparator* |  |  |  |  |  |  |  |  |
| Usual Care | 4 | -0.58 | -1.57-0.44 | .07 | 87.7% | <.01 | .30 | Random effects model |
| Without PR | 4 | -1.01 | -2.33-0.31 | .01 | 84.8% | <.01 |  | Random effects model |
| Written material and pedometer | 2 | -0.18 | -0.71-0.36 | .22 | 0%^b^ | .77 |  | Random effects model |
| *Setting* |  |  |  |  |  |  |  |  |
| Home-based | 8 | -0.61 | -1.18-(-0.03) | .04^a^ | 92.5% | <.01 | .71 | Random effects model |
| Community | 2 | -0.83 | -9.08-7.43 | .42 | 93.7% | <.01 |  | Random effects model |
| Hospital-based | / | / | / | / | / | / |  |  |
| *Sex proportion* |  |  |  |  |  |  |  |  |
| Male percentage<62.75% | 5 | -0.63 | -1.42-0.17 | .04 | 91.1% | <.01 | .92 | Random effects model |
| Male percentage≥62.75% | 5 | -0.67 | -1.66-0.32 | .03 | 93.7% | <.01 |  | Random effects model |
| ***Exercise capacity/6MWT (meters)*** | | | | | | | | |
| *Type of mHealth intervention* | | | | | | | | |
| Computer | 9 | 28.50 | 3.50-53.49 | <.01^a^ | 87% | <.01 | .66 | Random effects model |
| Smartphone app | 4 | 13.83 | -2.07-29.73 | .37 | 0%^b^ | .74 |  | Random effects model |
| Telephone | 1 | 46 | 18.11-73.89 | / | / | / |  |  |
| *The total number of sample* | | | | | | | | |
| n<106 | 9 | 34.96 | 15.63-54.22 | <.01^a^ | 66% | <.01 | .14 | Random effects model |
| n≥106 | 5 | 13.43 | -22.12-48.98 | .29 | 87% | <.01 |  | Random effects model |
| *Duration of Intervention* | | | | | | | | |
| <6 months | 6 | 34.61 | 3.96-65.25 | .03^a^ | 82% | <.01 | .35 | Random effects model |
| ≥6 months | 8 | 19.96 | -2.14-42.06 | .07 | 80.5% | <.01 |  | Random effects model |
| *Continent* |  |  |  |  |  |  |  |  |
| Asia | 2 | 37.39 | -213.35-288.14 | .06 | 67.8% | .08 | .13 | Random effects model |
| Australia | 2 | 46.78 | 30.04-63.52 | <.01^a^ | 0%^b^ | .91 |  | Random effects model |
| Europe | 8 | 28.53 | 5.50-51.55 | <.01^a^ | 82% | <.01 |  | Random effects model |
| North America | 2 | -7.89 | -63.61-47.83 | .26 | 0%^b^ | .53 |  | Random effects model |
| *Comparator* |  |  |  |  |  |  |  |  |
| Usual Care | 7 | 16.56 | -2.29-35.38 | .08 | 53% | .05 | **<.01^d^** | Random effects model |
| Without PR | 5 | 51.19 | 30.49-71.89 | <.01^a^ | 49%^b^ | .10 |  | Random effects model |
| Written material and pedometer | 2 | -7.89 | -63.61-47.83 | .32 | 0%^b^ | .53 |  | Random effects model |
| *Setting* |  |  |  |  |  |  |  |  |
| Home-based | 11 | 32.14 | 13.09-51.19 | <.01^a^ | 81% | <.01 | .33 | Random effects model |
| Community | 2 | -0.28 | -85.61-85.05 | .97 | 0%^b^ | .32 |  | Random effects model |
| Hospital-based | 1 | 6.00 | -16.17-28.17 | / | / | / |  |  |
| *Sex proportion* |  |  |  |  |  |  |  |  |
| Male percentage<62.75% | 7 | 36.07 | 16.10-56.04 | <.01^a^ | 54% | .04 | .19 | Random effects model |
| Male percentage≥62.75% | 7 | 17.06 | -11.14-45.3 | .14 | 87% | <.01 |  | Random effects model |
| ***Quality of Life (total score of SGRQ)*** | | | | | | | | |
| *Type of mHealth intervention* | | | | | | | | |
| Computer | 9 | -5.25 | -11.39-0.89 | .08 | 93% | <.01 | .47 | Random effects model |
| Smartphone app | 3 | -2.26 | -9.07-4.55 | .11 | 15%^b^ | .31 |  | Random effects model |
| Telephone | 3 | 0.30 | -13.71-14.31 | .50 | 88% | <.01 |  | Random effects model |
| *The total number of sample* | | | | | | | | |
| n<106 | 7 | -4.31 | -9.28-0.66 | .04 | 79.1% | <.01 | .74 | Random effects model |
| n≥106 | 8 | -3.01 | -9.97-3.95 | .31 | 94% | <.01 |  | Random effects model |
| *Duration of Intervention* | | | | | | | | |
| <6 months | 8 | -4.28 | -8.39-(-0.17) | .04^a^ | 78.5% | <.01 | .69 | Random effects model |
| ≥6 months | 7 | -2.77 | -11.10-5.56 | .45 | 94.8% | <.01 |  | Random effects model |
| *Continent* |  |  |  |  |  |  |  |  |
| Asia | 2 | -13.83 | -124.37-96.72 | <.01 | 97% | <.01 | **.05^c^** | Random effects model |
| Australia | 1 | -6.0 | -10.25-(-1.75) | <.01^a^ | / | / |  |  |
| Europe | 9 | -2.22 | -6.38-1.95 | .26 | 87% | <.01 |  | Random effects model |
| North America | 3 | -0.36 | -3.73-3.01 | .69 | 0%^b^ | .65 |  | Random effects model |
| *Comparator* |  |  |  |  |  |  |  |  |
| Usual Care | 5 | -1.74 | -5.89-2.40 | .26 | 43% | .13 | .41 | Random effects model |
| Without PR | 6 | -6.54 | -17.01-3.94 | .011 | 96% | <.01 |  | Random effects model |
| Written material and pedometer | 4 | -1.76 | -6.75-3.22 | .28 | 68% | .03 |  | Random effects model |
| *Setting* |  |  |  |  |  |  |  |  |
| Home-based | 13 | -3.32 | -7.82-1.17 | .11 | 92% | <.01 | .75 | Random effects model |
| Community | 2 | -5.27 | -8.70-(-1.85) | <.01^a^ | 0%^b^ | .81 |  | Random effects model |
| Hospital-based | / | / | / | / | / | / |  |  |
| *Sex proportion* |  |  |  |  |  |  |  |  |
| Male percentage<62.75% | 6 | -4.78 | -14.50-4.94 | .21 | 94% | <.01 | .58 | Random effects model |
| Male percentage≥62.75% | 9 | -2.71 | -6.78-1.37 | .12 | 87% | <.01 |  | Random effects model |

^a^The p-value, along with the confidence interval, indicates a statistically significant result.

^b^Heterogeneity within this subgroup was low, with an I² value ≤50%

^c^The meta-regression results indicate borderline significance for both analyses: p = .085 suggests that the type of mHealth intervention may moderate its effectiveness on the mMRC dyspnea scale, while p = .053 indicates that geographic location (grouped by continent) may influence the intervention’s effectiveness on SGRQ quality of life.

^d^Meta-regression results indicate that the comparator type significantly moderated the effect of mHealth interventions on the 6MWT outcome (p <.001).

This document is a supplementary appendix to a full article published in the Journal of Medical Internet Research (J Med Internet Res). For complete copyright and citation details, please refer to the main manuscript XXX.

REFERENCES

38. Arbillaga-Etxarri A, Gimeno-Santos E, Barberan-Garcia A, et al. Long-term efficacy and effectiveness of a behavioural and community-based exercise intervention (urban training) to increase physical activity in patients with COPD: a randomised controlled trial. Eur Respir J. Oct 2018;52(4):1800063. [doi: 10.1183/13993003.00063-2018] [Medline: 30166322]

70. Benzo RP, Ridgeway J, Hoult JP, et al. Feasibility of a health coaching and home-based rehabilitation intervention with remote monitoring for COPD. Respir Care. Jun 2021;66(6):960-971. [doi: 10.4187/respcare.08580] [Medline: 33906954]

71. Benzo R, Hoult J, McEvoy C, et al. Promoting chronic obstructive pulmonary disease wellness through remote monitoring and health coaching: a clinical trial. Ann Am Thorac Soc. Nov 2022;19(11):1808-1817. [doi: 10.1513/AnnalsATS.202203-214OC] [Medline: 35914215]

41. Boer L, Bischoff E, van der Heijden M, et al. A smart mobile health tool versus a paper action plan to support self-management of chronic obstructive pulmonary disease exacerbations: randomized controlled trial. JMIR Mhealth Uhealth. Oct 9, 2019;7(10):e14408. [doi: 10.2196/14408] [Medline: 31599729]

42. Bourne S, DeVos R, North M, et al. Online versus face-to-face pulmonary rehabilitation for patients with chronic obstructive pulmonary disease: randomised controlled trial. BMJ Open. Jul 17, 2017;7(7):e014580. [doi: 10.1136/bmjopen-2016-014580] [Medline: 28716786]

57. Chan HY, Dai YT, Hou IC. Evaluation of a tablet-based instruction of breathing technique in patients with COPD. Int J Med Inform. Oct 2016;94(263-70):263-270. [doi: 10.1016/j.ijmedinf.2016.06.018] [Medline: 27573335]\

43. Farmer A, Williams V, Velardo C, et al. Self-management support using a digital health system compared with usual care for chronic obstructive pulmonary disease: randomized controlled trial. J Med Internet Res. May 3, 2017;19(5):e144. [doi: 10.2196/jmir.7116] [Medline: 28468749]

58. Ho TW, Huang CT, Chiu HC, et al. Effectiveness of telemonitoring in patients with chronic obstructive pulmonary disease in taiwan-a randomized controlled trial. Sci Rep. Mar 31, 2016;6(23797):23797. [doi: 10.1038/srep23797] [Medline: 27029815]

49. Kessler R, Casan-Clara P, Koehler D, et al. COMET: a multicomponent home-based disease-management programme versus routine care in severe COPD. Eur Respir J. Jan 2018;51(1):29326333. [doi: 10.1183/13993003.01612-2017] [Medline: 29326333]

67. Moy ML, Martinez CH, Kadri R, et al. Long-term effects of an internet-mediated pedometer-based walking program for chronic obstructive pulmonary disease: randomized controlled trial. J Med Internet Res. Aug 8, 2016;18(8):e215. [doi: 10.2196/jmir.5622] [Medline: 27502583]

44. Rixon L, Hirani SP, Cartwright M, et al. A RCT of telehealth for COPD patient’s quality of life: the whole system demonstrator evaluation. Clin Respir J. Jul 2017;11(4):459-469. [doi: 10.1111/crj.12359] [Medline: 26260325]

72. Robinson SA, Cooper JA Jr, Goldstein RL, et al. A randomised trial of a web-based physical activity self-management intervention in COPD. ERJ Open Res. Jul 2021;7(3):00158-2021. [doi: 10.1183/23120541.00158-2021] [Medline: 34476247]

50. Saleh S, Skeie S, Grundt H. Re-admission and quality of life among patients with chronic obstructive pulmonary disease after telemedicine video nursing consultation - a randomized study. Multidiscip Respir Med. Jan 17, 2023;18(1):918. [doi: 10.4081/mrm.2023.918] [Medline: 37753200]

73. Stamenova V, Liang K, Yang R, et al. Technology-enabled self-management of chronic obstructive pulmonary disease with or without asynchronous remote monitoring: randomized controlled trial. J Med Internet Res. Jul 30, 2020;22(7):e18598. [doi: 10.2196/18598] [Medline: 32729843]

55. Vasilopoulou M, Papaioannou AI, Kaltsakas G, et al. Home-based maintenance tele-rehabilitation reduces the risk for acute exacerbations of COPD, hospitalisations and emergency department visits. Eur Respir J. May 2017;49(5):1602129. [doi: 10.1183/13993003.02129-2016] [Medline: 28546268]

52. Vianello A, Fusello M, Gubian L, et al. Home telemonitoring for patients with acute exacerbation of chronic obstructive pulmonary disease: a randomized controlled trial. BMC Pulm Med. Nov 22, 2016;16(1):157. [doi: 10.1186/s12890-016-0321-2] [Medline: 27876029]

45. Walker PP, Pompilio PP, Zanaboni P, et al. Telemonitoring in Chronic Obstructive Pulmonary Disease (CHROMED). A Randomized Clinical Trial. Am J Respir Crit Care Med. Sep 1, 2018;198(5):620-628. [doi: 10.1164/rccm.201712-2404OC] [Medline: 29557669]

68. Wan ES, Kantorowski A, Homsy D, et al. Promoting physical activity in COPD: insights from a randomized trial of a web-based intervention and pedometer use. Respir Med. Sep 2017;130(102-10):102-110. [doi: 10.1016/j.rmed.2017.07.057] [Medline: 29206627]

69. Wan ES, Kantorowski A, Polak M, et al. Long-term effects of web-based pedometer-mediated intervention on COPD exacerbations. Respir Med. Feb 2020;162:105878. [doi: 10.1016/j.rmed.2020.105878] [Medline: 32056676]

59. Wang L, He L, Tao Y, et al. Evaluating a web-based coaching program using electronic health records for patients with chronic obstructive pulmonary disease in China: randomized controlled trial. J Med Internet Res. Jul 21, 2017;19(7):e264. [doi: 10.2196/jmir.6743] [Medline: 28733270]

51. Zanaboni P, Dinesen B, Hoaas H, et al. Long-term telerehabilitation or unsupervised training at home for patients with chronic obstructive pulmonary disease: a randomized controlled trial. Am J Respir Crit Care Med. Apr 1, 2023;207(7):865-875. [doi: 10.1164/rccm.202204-0643OC] [Medline: 36480957]

46. Jolly K, Sidhu MS, Hewitt CA, et al. Self management of patients with mild COPD in primary care: randomised controlled trial. BMJ. Jun 13, 2018;361:k2241. [doi: 10.1136/bmj.k2241] [Medline: 29899047]

39. Varas AB, Córdoba S, Rodríguez-Andonaegui I, Rueda MR, García-Juez S, Vilaró J. Effectiveness of a community-based exercise training programme to increase physical activity level in patients with chronic obstructive pulmonary disease: a randomized controlled trial. Physiother Res Int. Oct 2018;23(4):e1740. [doi: 10.1002/pri.1740] [Medline: 30168228]

66. Wootton SL, McKeough Z, Ng CLW, et al. Effect on health-related quality of life of ongoing feedback during a 12-month maintenance walking programme in patients with COPD: a randomized controlled trial. Respirology. Jan 2018;23(1):60-67. [doi: 10.1111/resp.13128] [Medline: 28758320]

60. Bi J, Yang W, Hao P, et al. WeChat as a platform for baduanjin intervention in patients with stable chronic obstructive pulmonary disease in China: retrospective randomized controlled trial. JMIR Mhealth Uhealth. Feb 2, 2021;9(2):e23548. [doi: 10.2196/23548] [Medline: 33528369]

53. Cerdán-de-las-Heras J, Balbino F, Løkke A, Catalán-Matamoros D, Hilberg O, Bendstrup E. Effect of a new tele-rehabilitation program versus standard rehabilitation in patients with chronic obstructive pulmonary disease. JCM. 2022;11(1):11. [doi: 10.3390/jcm11010011]

47. Crooks MG, Elkes J, Storrar W, et al. Evidence generation for the clinical impact of myCOPD in patients with mild, moderate and newly diagnosed COPD: a randomised controlled trial. ERJ Open Res. Oct 2020;6(4):1-10. [doi: 10.1183/23120541.00460-2020] [Medline: 33263052]

61. Jiang Y, Liu F, Guo J, et al. Evaluating an intervention program using WeChat for patients with chronic obstructive pulmonary disease: randomized controlled trial. J Med Internet Res. Apr 21, 2020;22(4):e17089. [doi: 10.2196/17089] [Medline: 32314971]

40. Jiménez-Reguera B, Maroto López E, Fitch S, et al. Development and preliminary evaluation of the effects of an mHealth web-based platform (HappyAir) on adherence to a maintenance program after pulmonary rehabilitation in patients with chronic obstructive pulmonary disease: randomized controlled trial. JMIR Mhealth Uhealth. Jul 31, 2020;8(7):e18465. [doi: 10.2196/18465] [Medline: 32513646]

56. Loeckx M, Rodrigues FM, Blondeel A, et al. Sustaining training effects through physical activity coaching (STEP): a randomized controlled trial. Int J Behav Nutr Phys Act. Oct 10, 2023;20(1):121. [doi: 10.1186/s12966-023-01519-w] [Medline: 37814266]

48. North M, Bourne S, Green B, et al. A randomised controlled feasibility trial of E-health application supported care vs usual care after exacerbation of COPD: the RESCUE trial. NPJ Digit Med. 2020;3(1):145. [doi: 10.1038/s41746-020-00347-7] [Medline: 33145441]

63. Park SK, Bang CH, Lee SH. Evaluating the effect of a smartphone app-based self-management program for people with COPD: a randomized controlled trial. Appl Nurs Res. Apr 2020;52:151231. [doi: 10.1016/j.apnr.2020.151231] [Medline: 31955942]

54. Spielmanns M, Gloeckl R, Jarosch I, et al. Using a smartphone application maintains physical activity following pulmonary rehabilitation in patients with COPD: a randomised controlled trial. Thorax. May 2023;78(5):442-450. [doi: 10.1136/thoraxjnl-2021-218338] [Medline: 35450945]

62. Wang L, Guo Y, Wang M, Zhao Y. A mobile health application to support self-management in patients with chronic obstructive pulmonary disease: a randomised controlled trial. Clin Rehabil. Jan 2021;35(1):90-101. [doi: 10.1177/0269215520946931]ss
